# Supplementary material for: Multiple UBX proteins reduce the ubiquitin threshold of the mammalian p97-UFD1-NPL4 unfoldase
Source: eLife. 2022 Aug 3;11:e76763. doi: 10.7554/eLife.76763 (PMC9377798; doi:10.7554/eLife.76763)

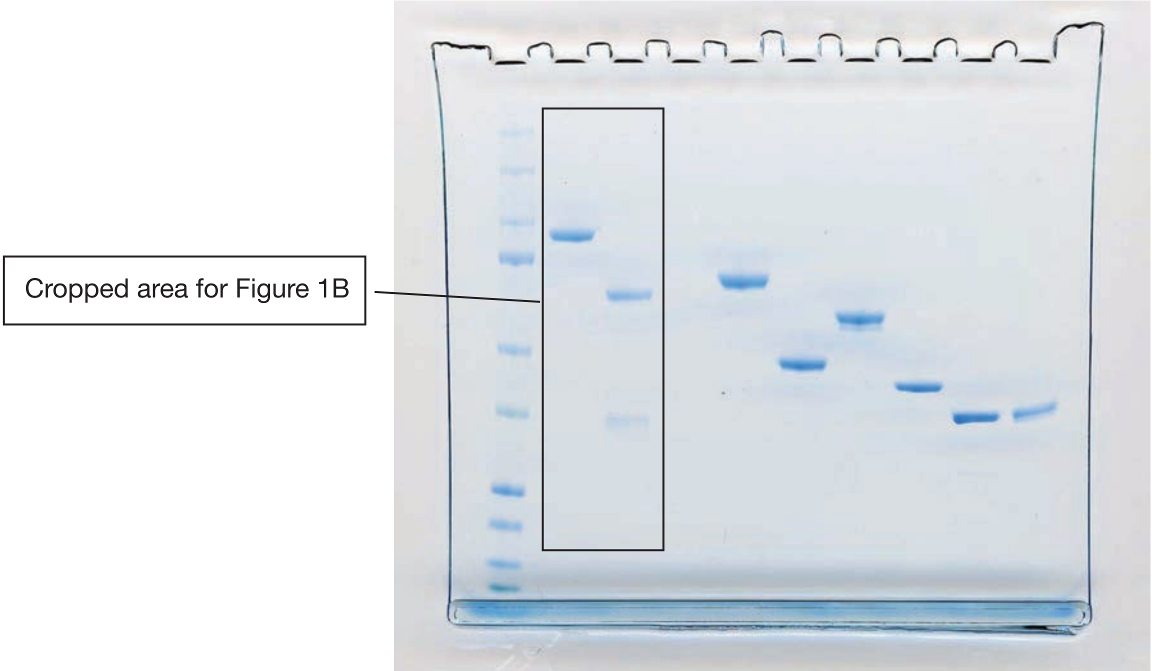
Cropped area for Figure 1B

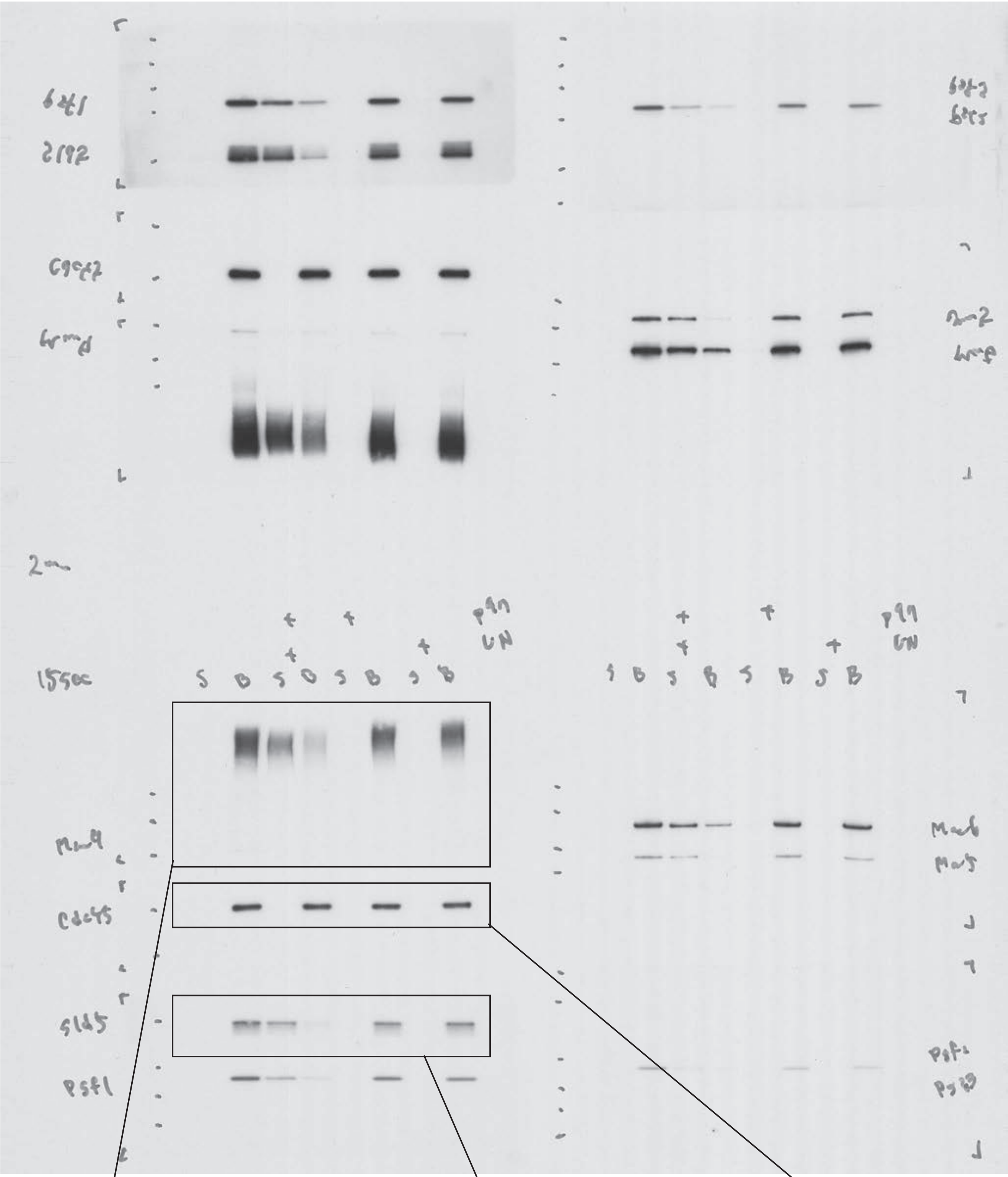

Cropped area for Figure 1C
Mcm7

Cropped area for Figure 1C
Sld5

Cropped area for Figure 1C
Cdc45

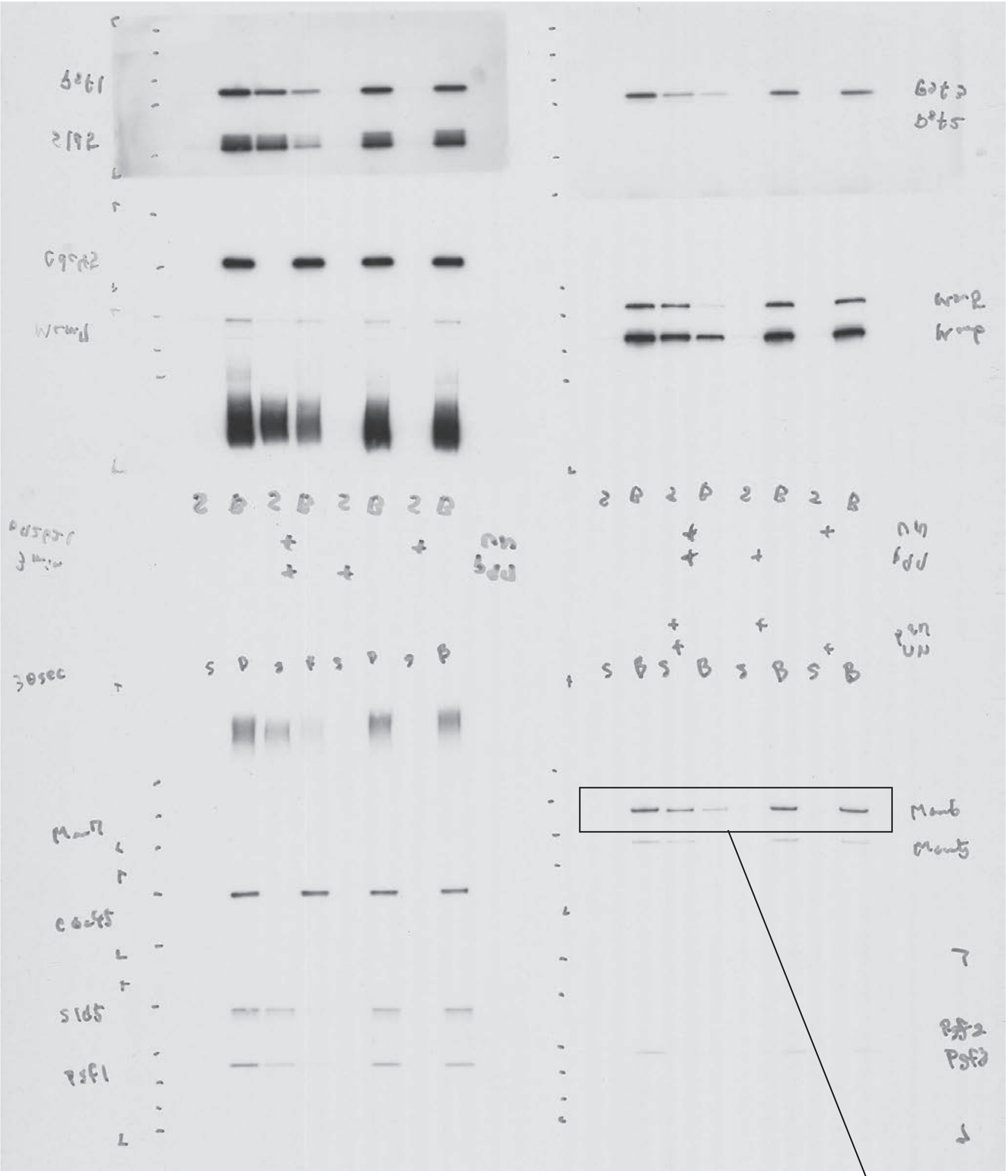

Cropped area for Figure 1C
Mcm6

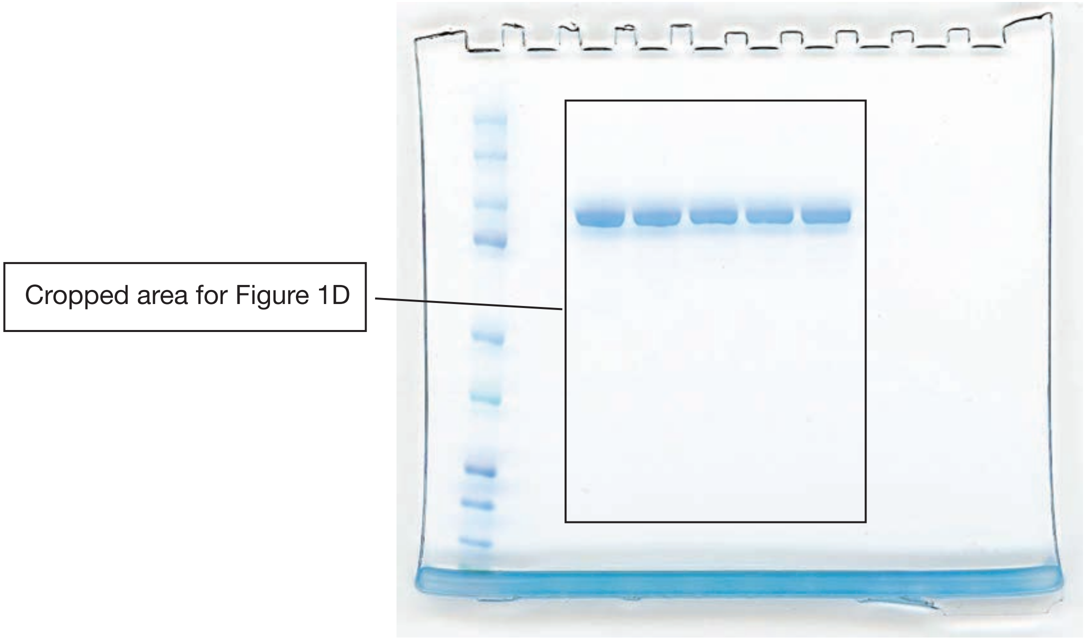
Cropped area for Figure 1D

Cropped area for Figure 1E
Sld5

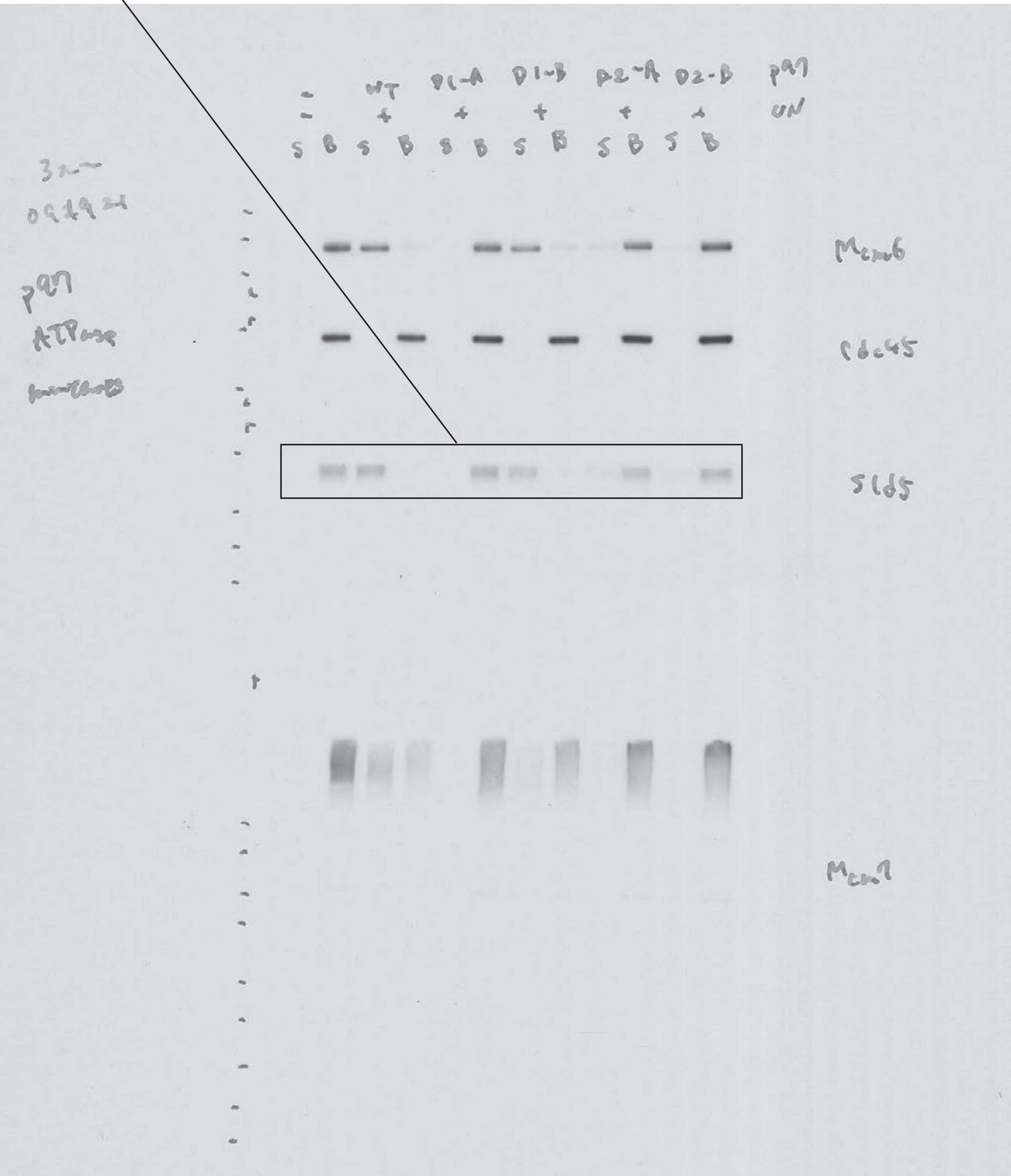

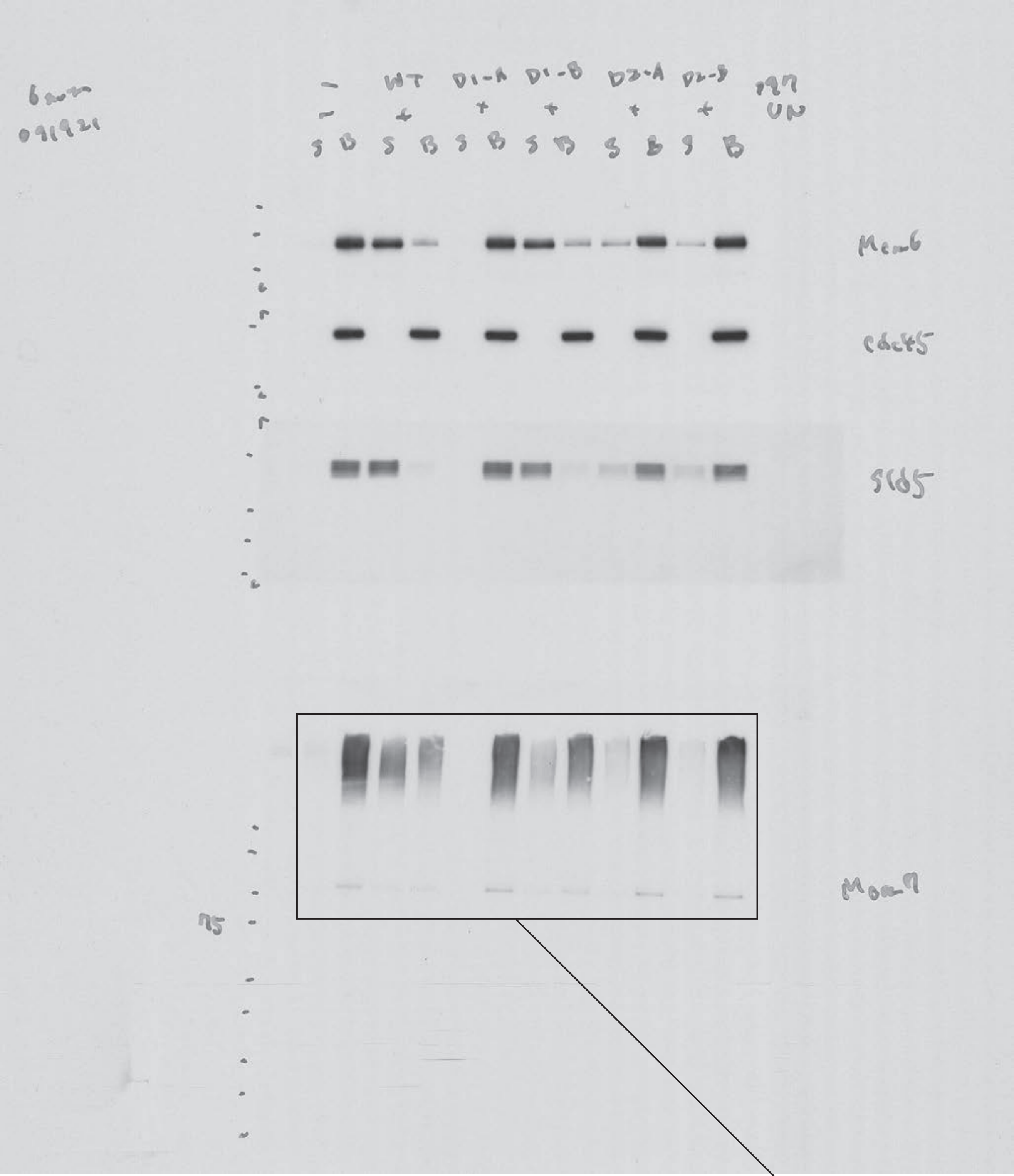

Cropped area for Figure 1E
Mcm7

Cropped area for Figure 1E
Cdc45

Cropped area for Figure 1E
Mcm6

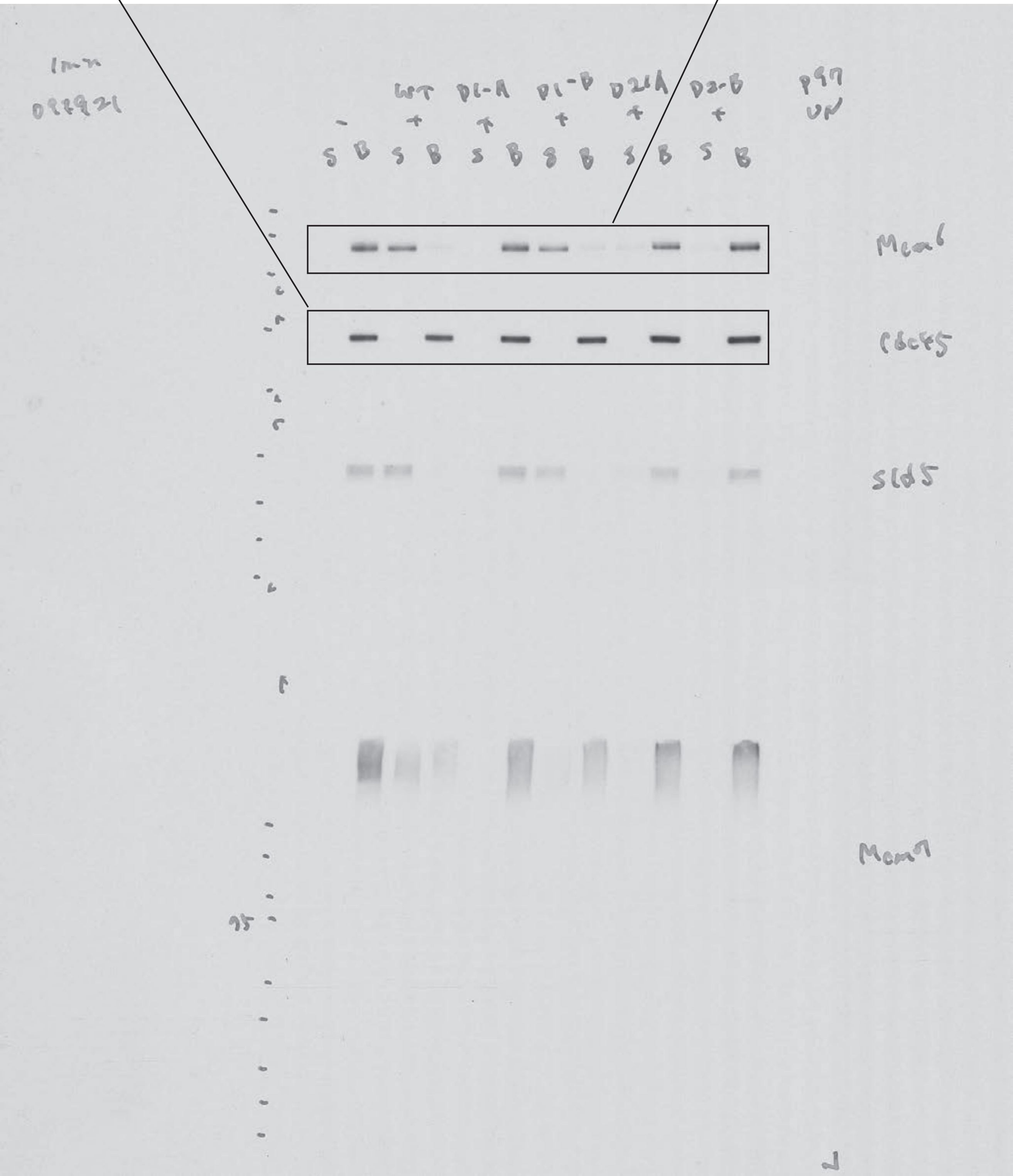

Supplement: Figure 1—source data 1. [file elife-76763-fig1-data1.pdf]
