# Supplementary material for: Multiple UBX proteins reduce the ubiquitin threshold of the mammalian p97-UFD1-NPL4 unfoldase
Source: eLife. 2022 Aug 3;11:e76763. doi: 10.7554/eLife.76763 (PMC9377798; doi:10.7554/eLife.76763)
Supplement: Figure 1—figure supplement 1—source data 1. [file elife-76763-fig1-figsupp1-data1.pdf]

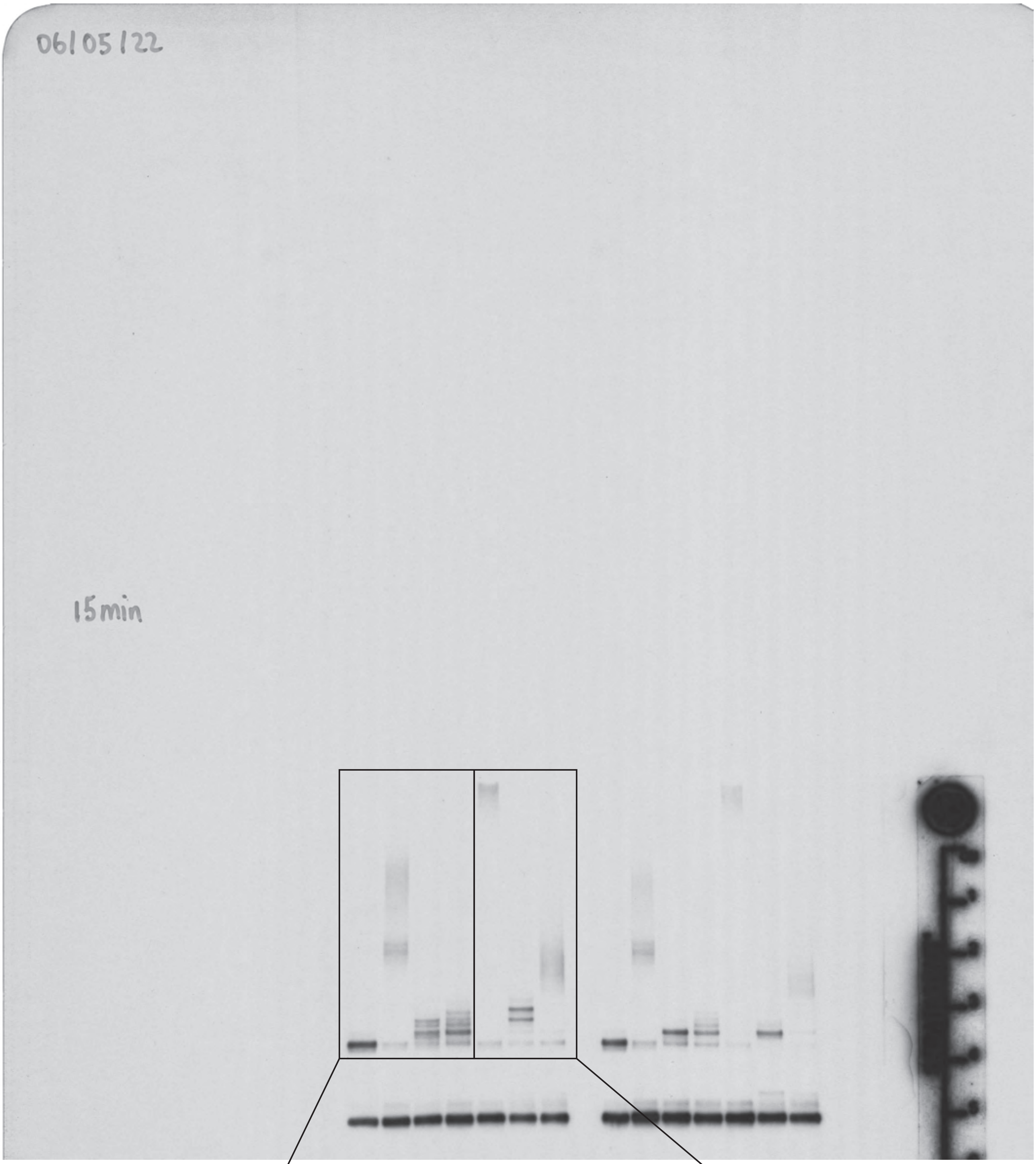

Cropped area for Figure 1-figure supplement 1D
Mcm7

Cropped area for Figure 1-figure supplement 1A
Mcm7

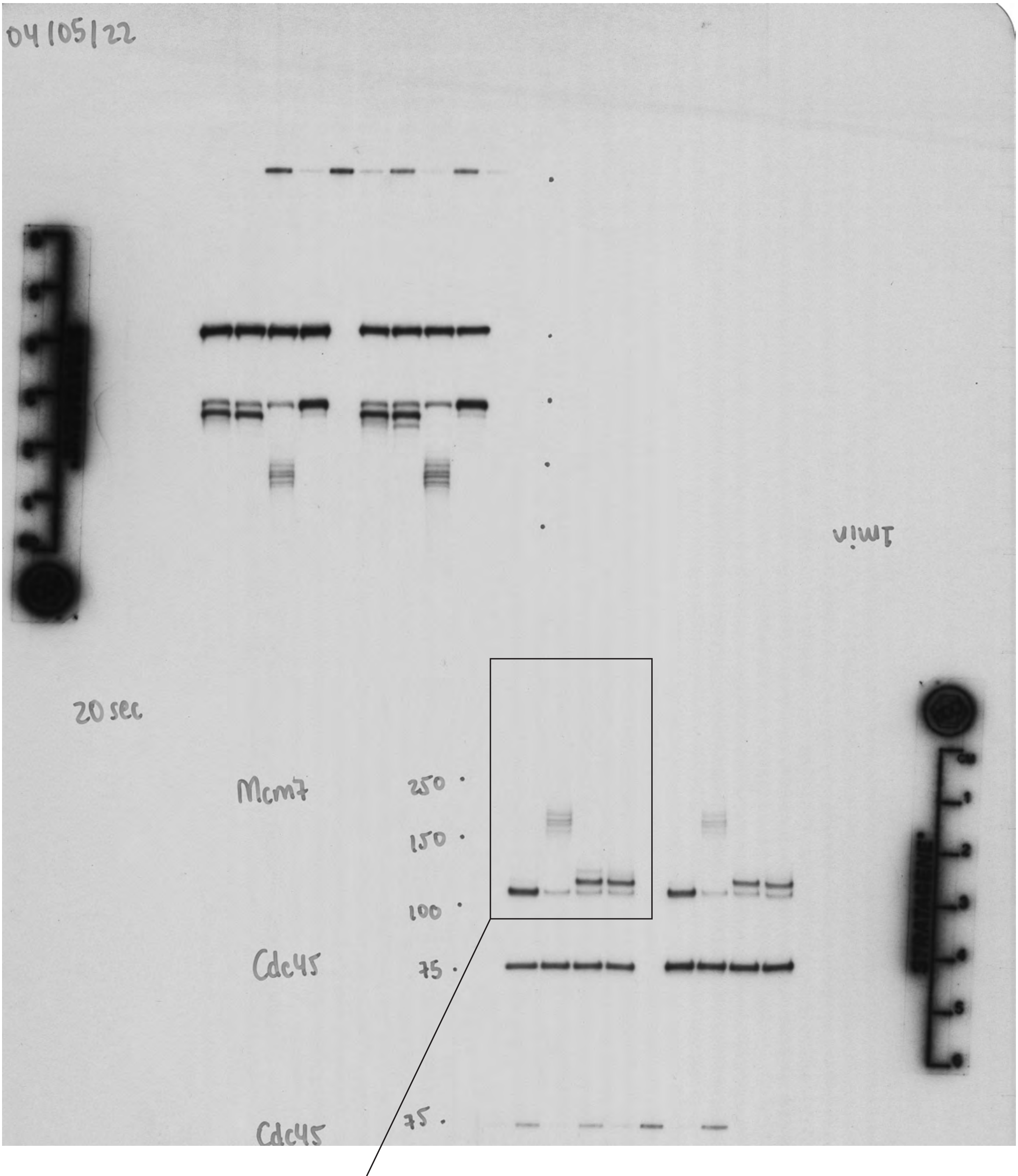

Cropped area for Figure 1-figure supplement 1B
Mcm7

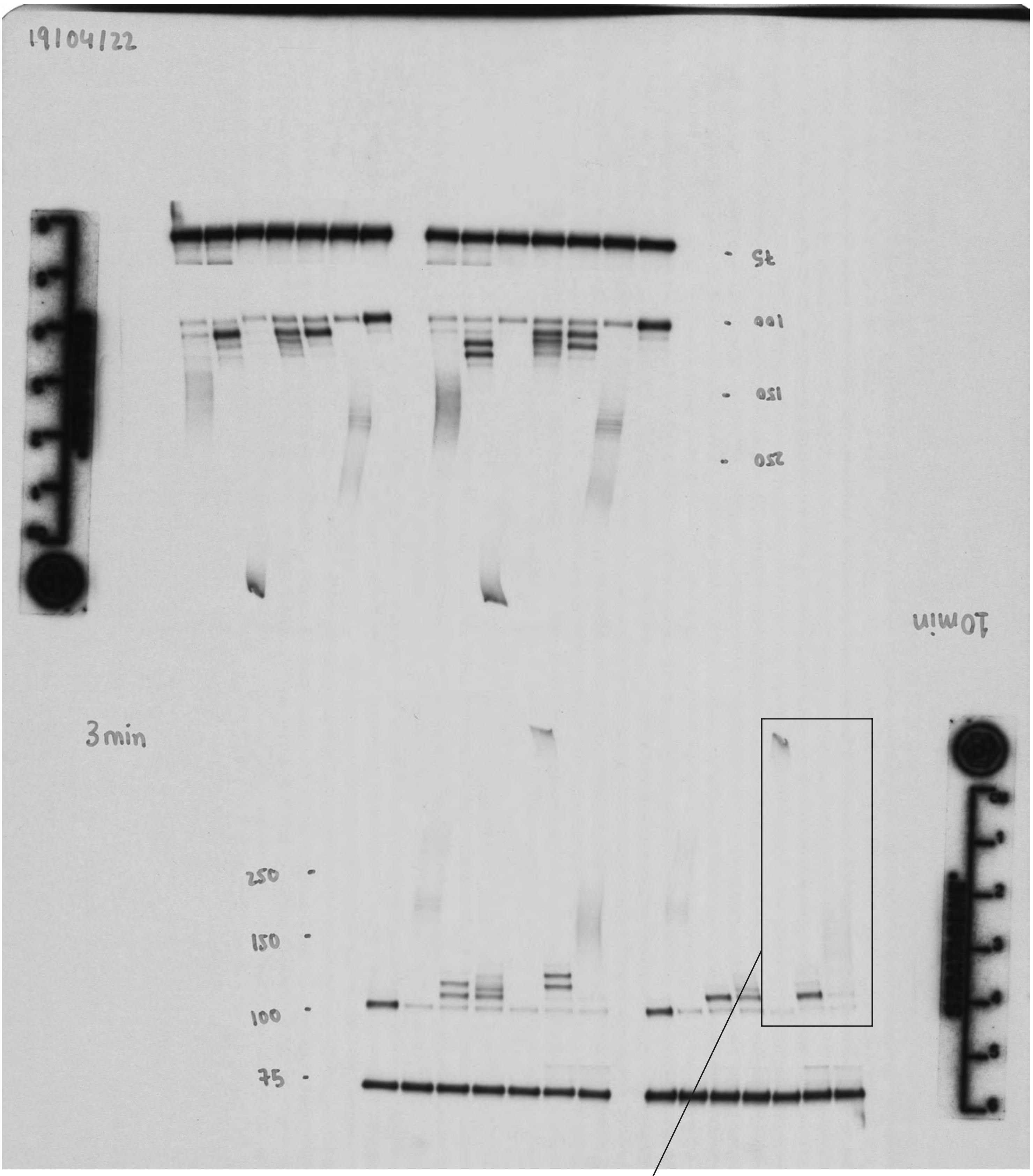

Cropped area for Figure 1-figure supplement 1C
Mcm7
