# Supplementary material for: Multiple UBX proteins reduce the ubiquitin threshold of the mammalian p97-UFD1-NPL4 unfoldase
Source: eLife. 2022 Aug 3;11:e76763. doi: 10.7554/eLife.76763 (PMC9377798; doi:10.7554/eLife.76763)
Supplement: Figure 2—source data 1. [file elife-76763-fig2-data1.pdf]

Cropped area for Figure 2B left panels: Mcm6

Cropped area for Figure 2B centre panels: Mcm6

Cropped area for Figure 2B right panels: Mcm6

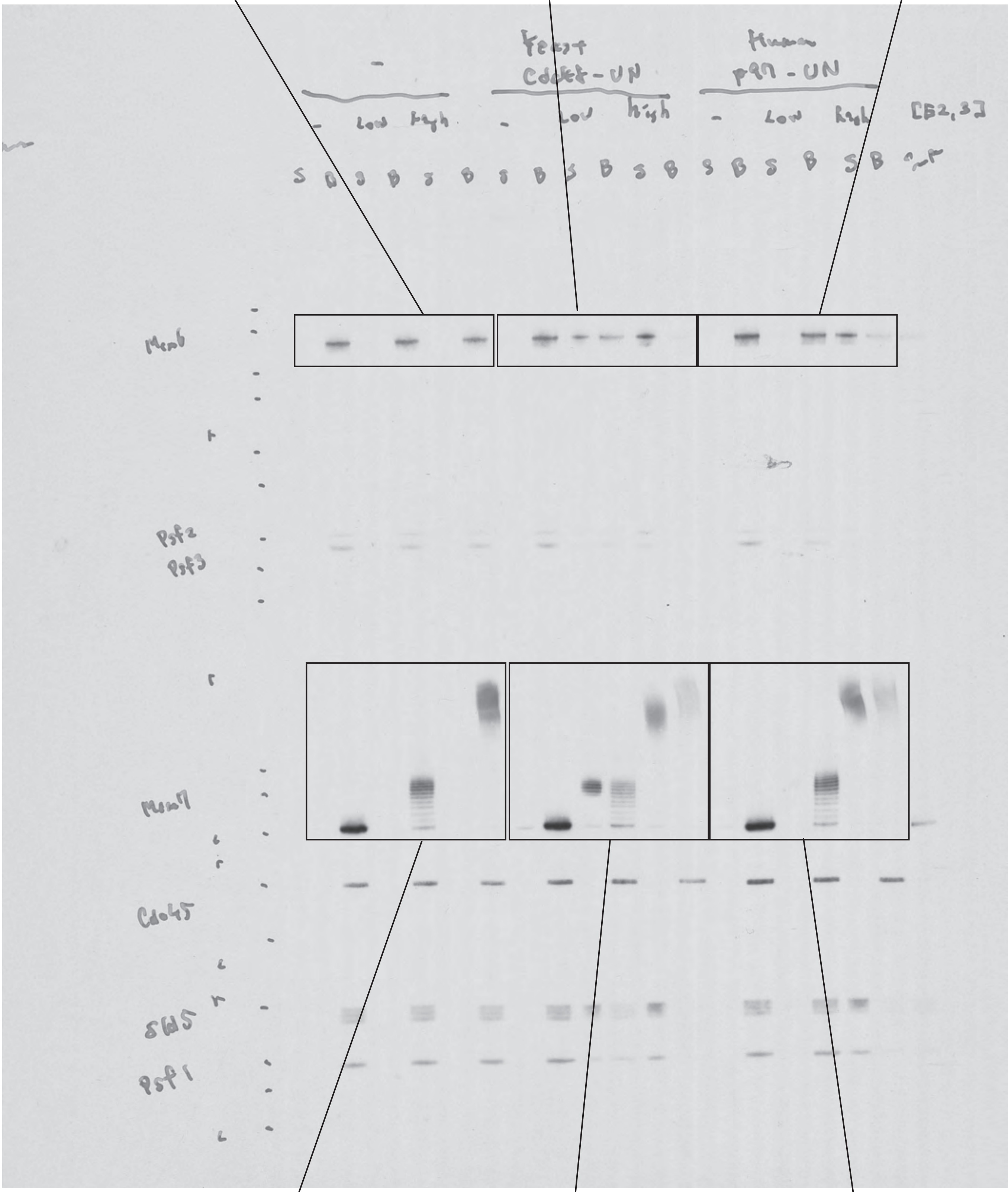

Cropped area for Figure 2B left panels: Mcm7

Cropped area for Figure 2B centre panels: Mcm7

Cropped area for Figure 2B right panels: Mcm7

Cropped area for Figure 2B left panels: Cdc45

Cropped area for Figure 2B centre panels: Cdc45

Cropped area for Figure 2B right panels: Cdc45

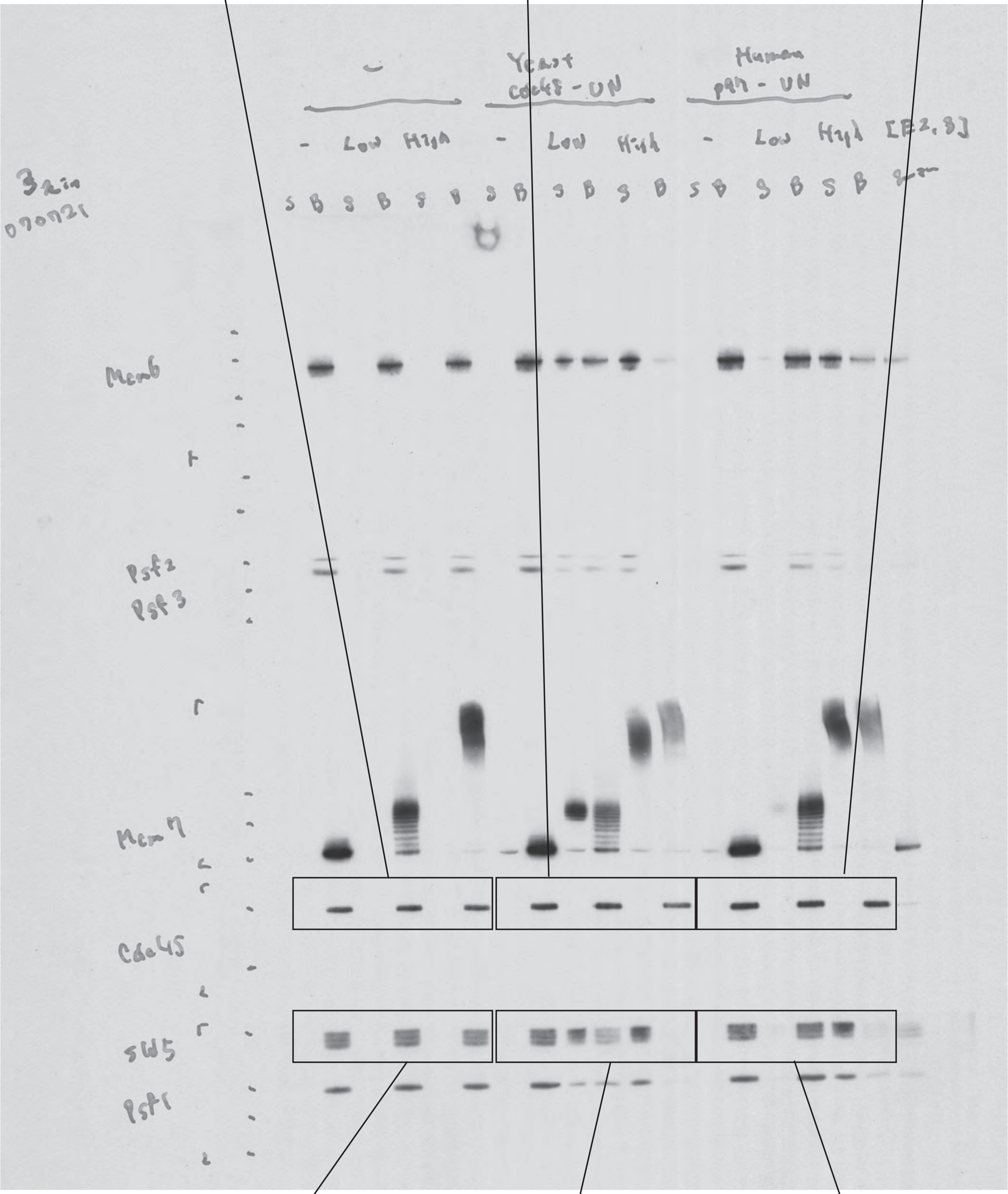

Cropped area for Figure 2B left panels: Sld5

Cropped area for Figure 2B centre panels: Sld5

Cropped area for Figure 2B right panels: Sld5
