# Supplementary material for: Multiple UBX proteins reduce the ubiquitin threshold of the mammalian p97-UFD1-NPL4 unfoldase
Source: eLife. 2022 Aug 3;11:e76763. doi: 10.7554/eLife.76763 (PMC9377798; doi:10.7554/eLife.76763)
Supplement: Figure 2—figure supplement 1—source data 1. [file elife-76763-fig2-figsupp1-data1.pdf]

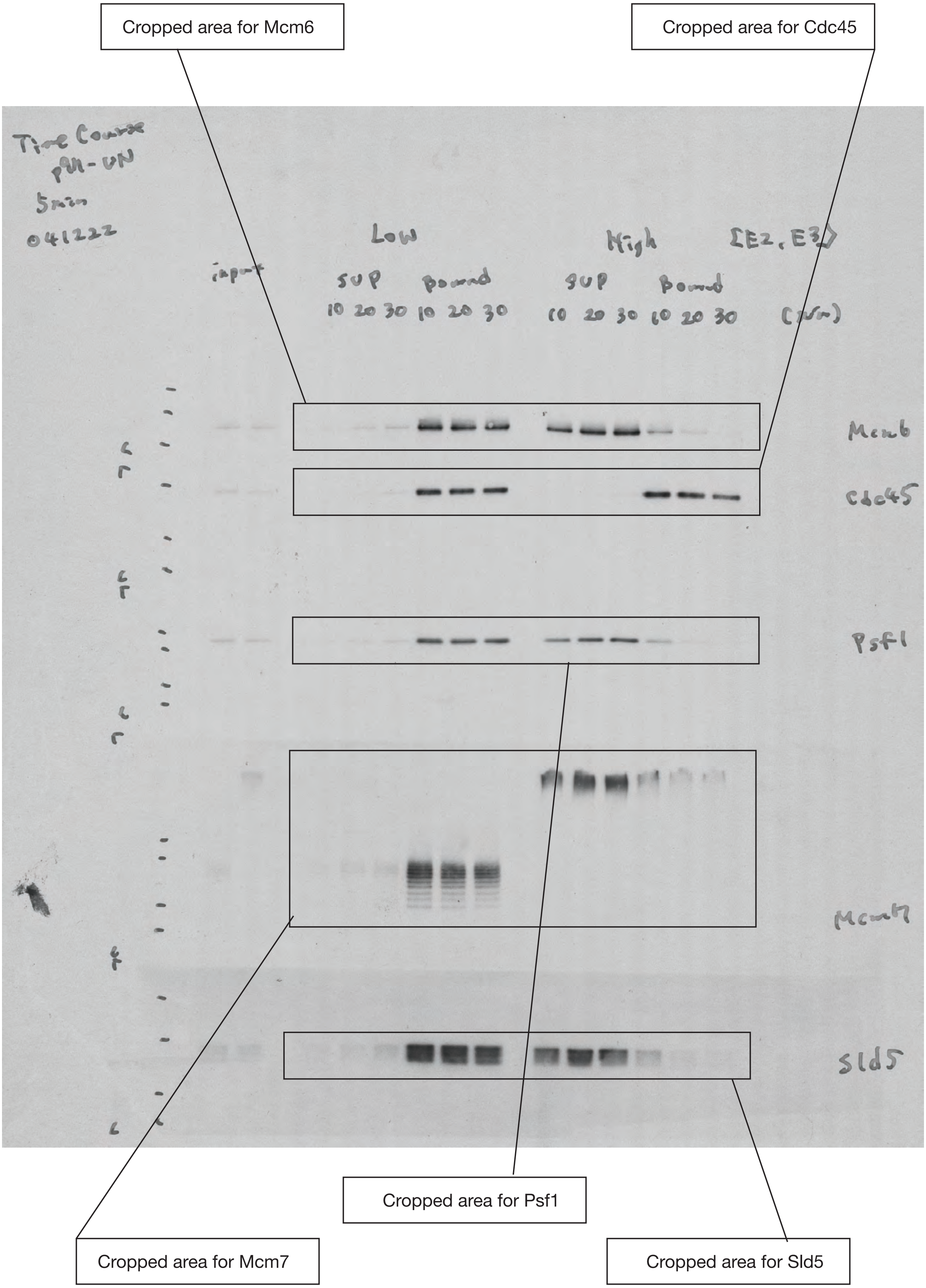

Cropped area for Mcm6
Cropped area for Cdc45
Time Course
Low
High
SUP
Bound
10 20 30 10 20 30
10 20 30 10 20 30
(min)
Mcm6
Cdc45
Psf1
Mcm7
Sld5
Cropped area for Psf1
Cropped area for Mcm7
Cropped area for Sld5
