# Supplementary material for: Multiple UBX proteins reduce the ubiquitin threshold of the mammalian p97-UFD1-NPL4 unfoldase
Source: eLife. 2022 Aug 3;11:e76763. doi: 10.7554/eLife.76763 (PMC9377798; doi:10.7554/eLife.76763)
Supplement: Figure 2—figure supplement 2—source data 1. [file elife-76763-fig2-figsupp2-data1.pdf]

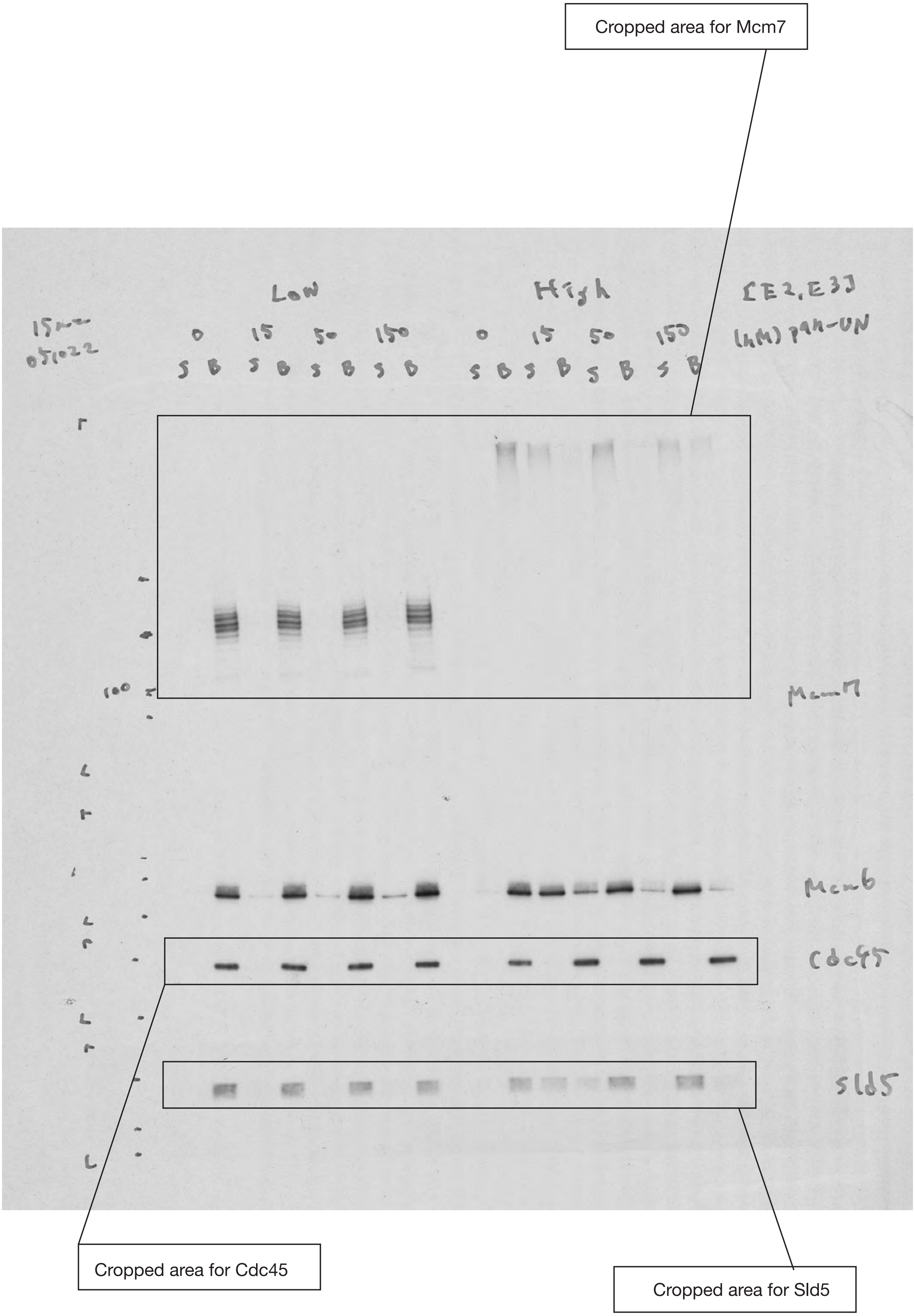
Cropped area for Mcm7
Low
High
[E2, E3]
0 15 50 150
0 15 50 150
S B S B S B S B
S B S B S B S B
100
Mcm7
Mcm6
Cdc45
Sld5
Cropped area for Cdc45
Cropped area for Sld5

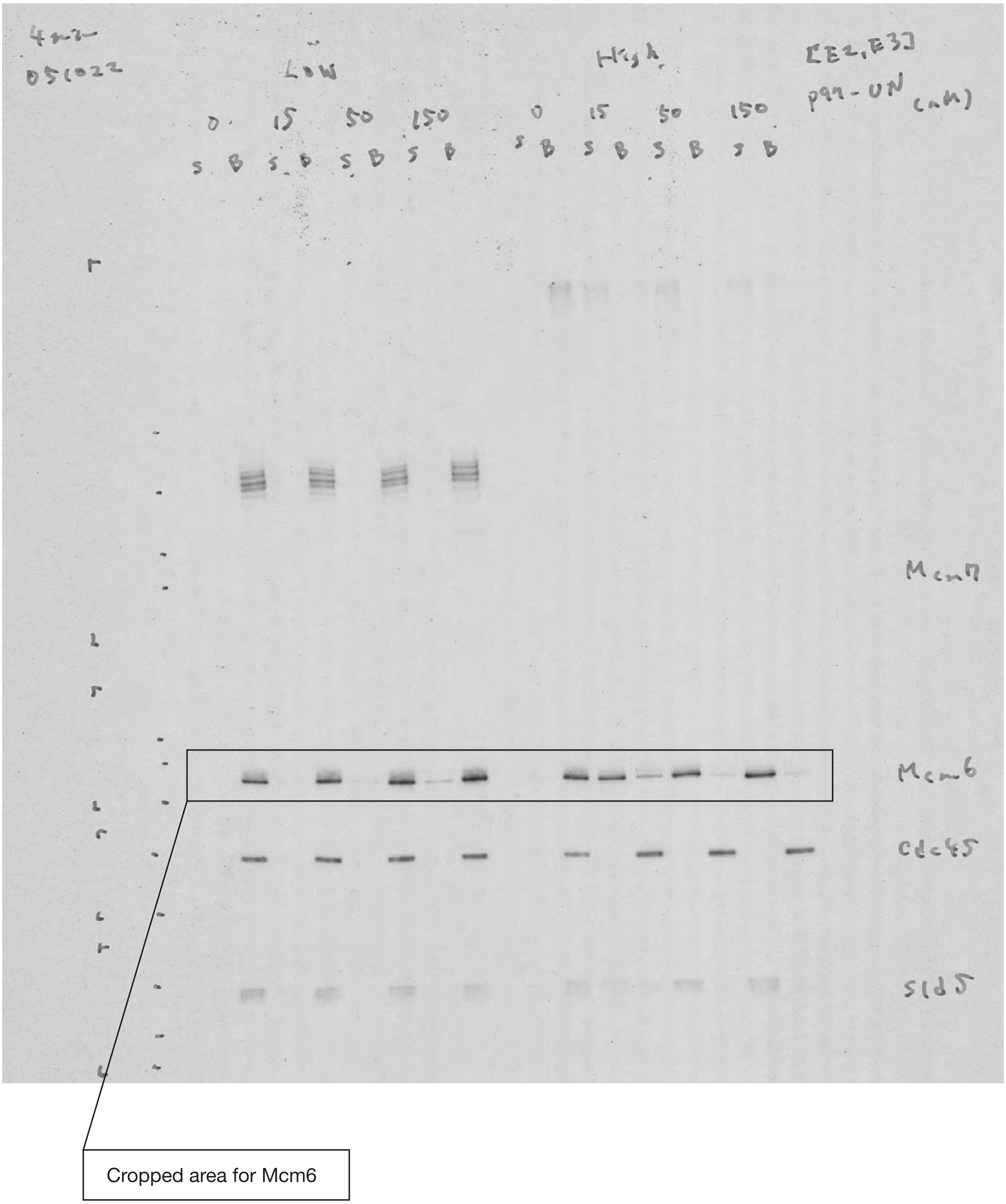
Low
High
Mcm6
Cdc45
Sld5
Cropped area for Mcm6
