# Supplementary material for: Multiple UBX proteins reduce the ubiquitin threshold of the mammalian p97-UFD1-NPL4 unfoldase
Source: eLife. 2022 Aug 3;11:e76763. doi: 10.7554/eLife.76763 (PMC9377798; doi:10.7554/eLife.76763)
Supplement: Figure 2—figure supplement 3—source data 1. [file elife-76763-fig2-figsupp3-data1.pdf]

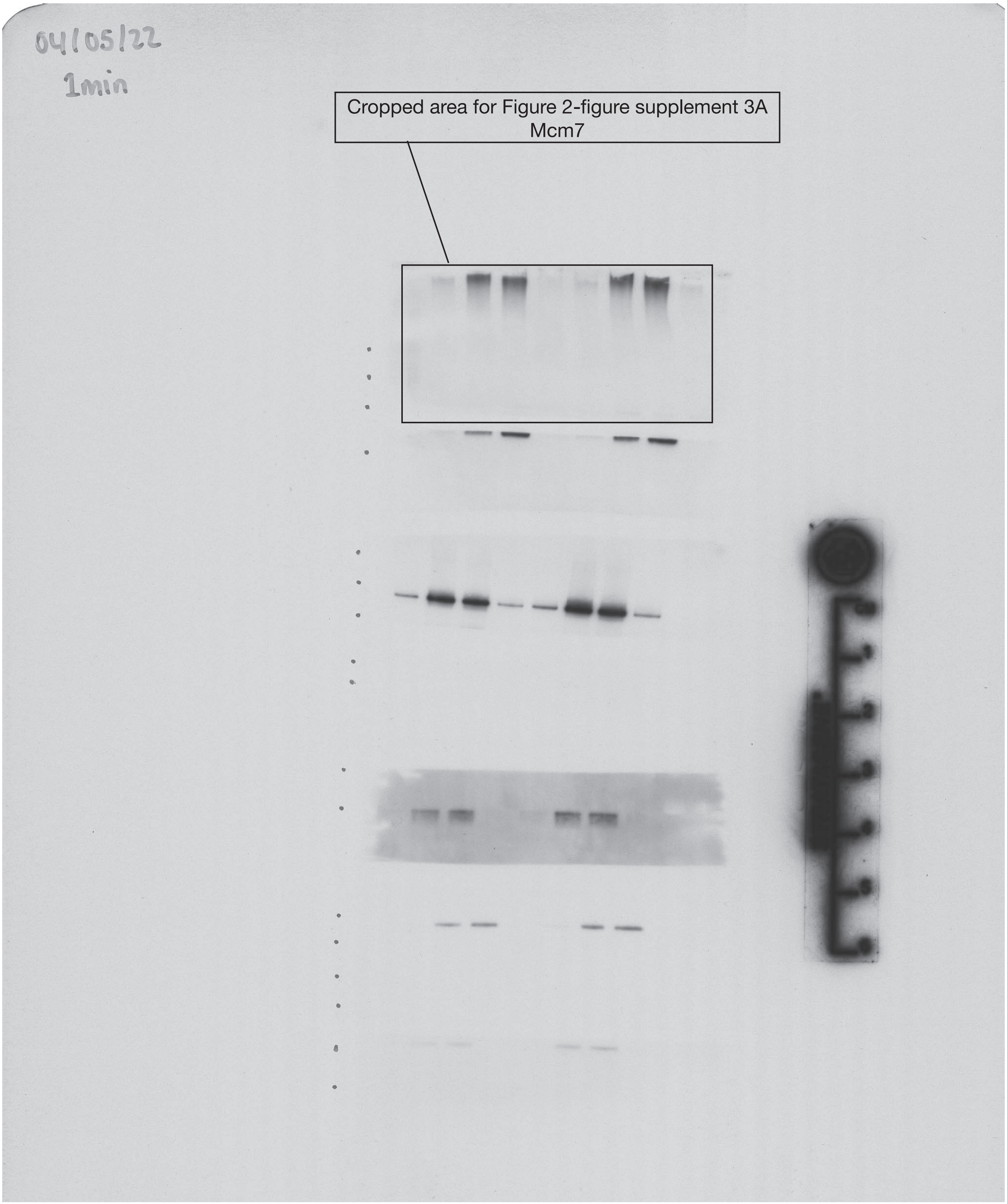
04/05/22
1min
Cropped area for Figure 2-figure supplement 3A
Mcm7

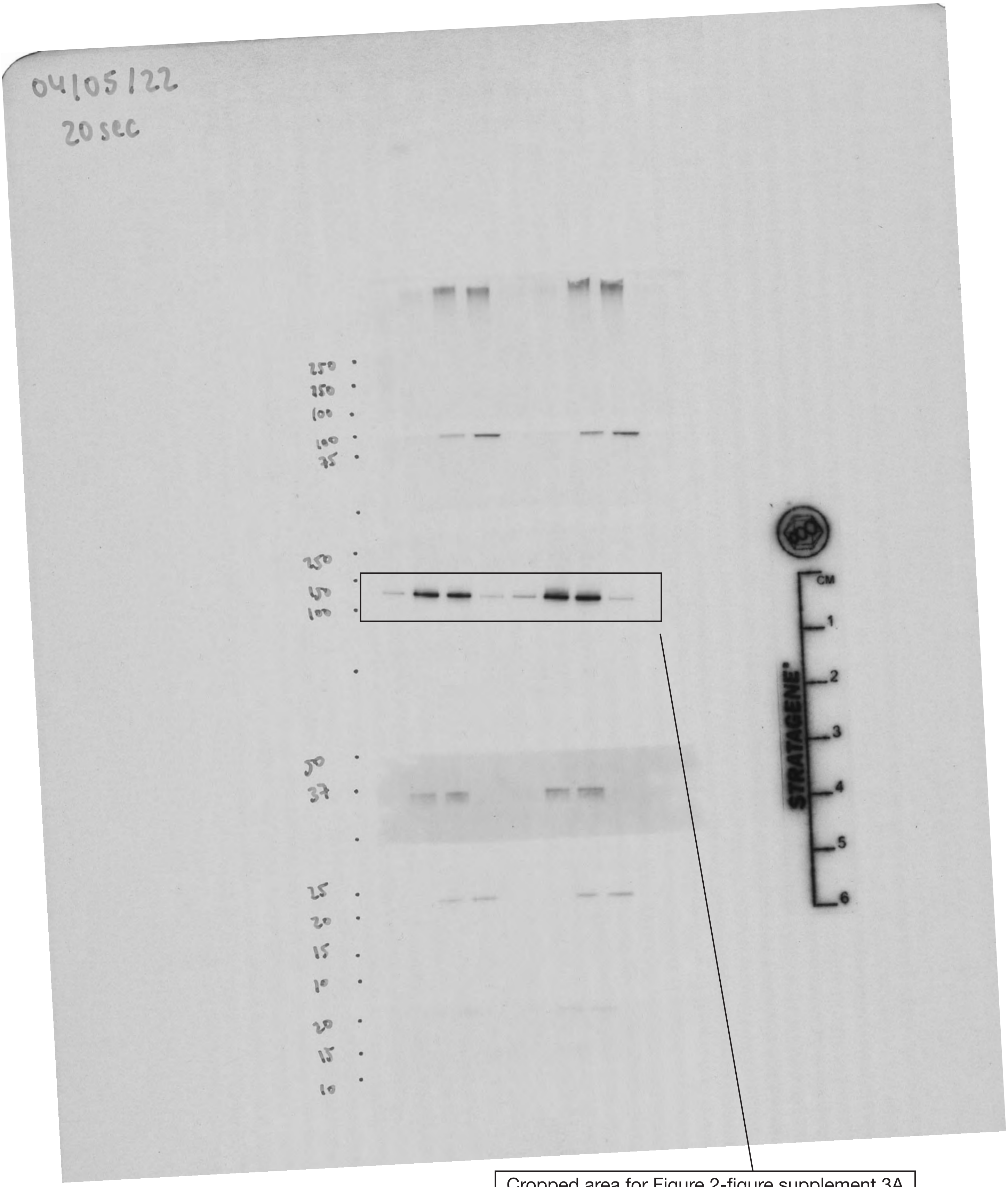

Cropped area for Figure 2-figure supplement 3A
Mcm6

Cropped area for Figure 2-figure supplement 3A
Cdc45

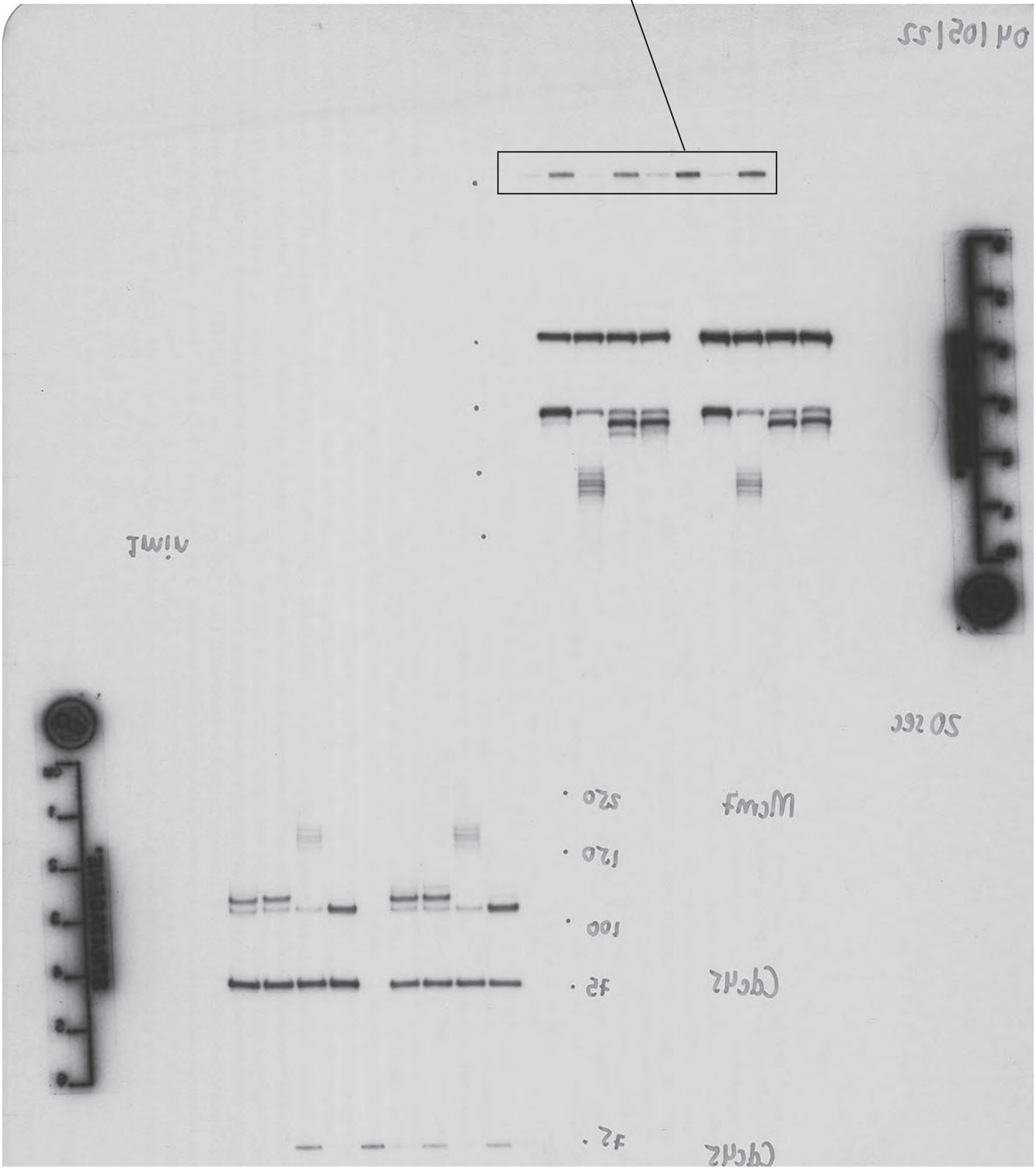

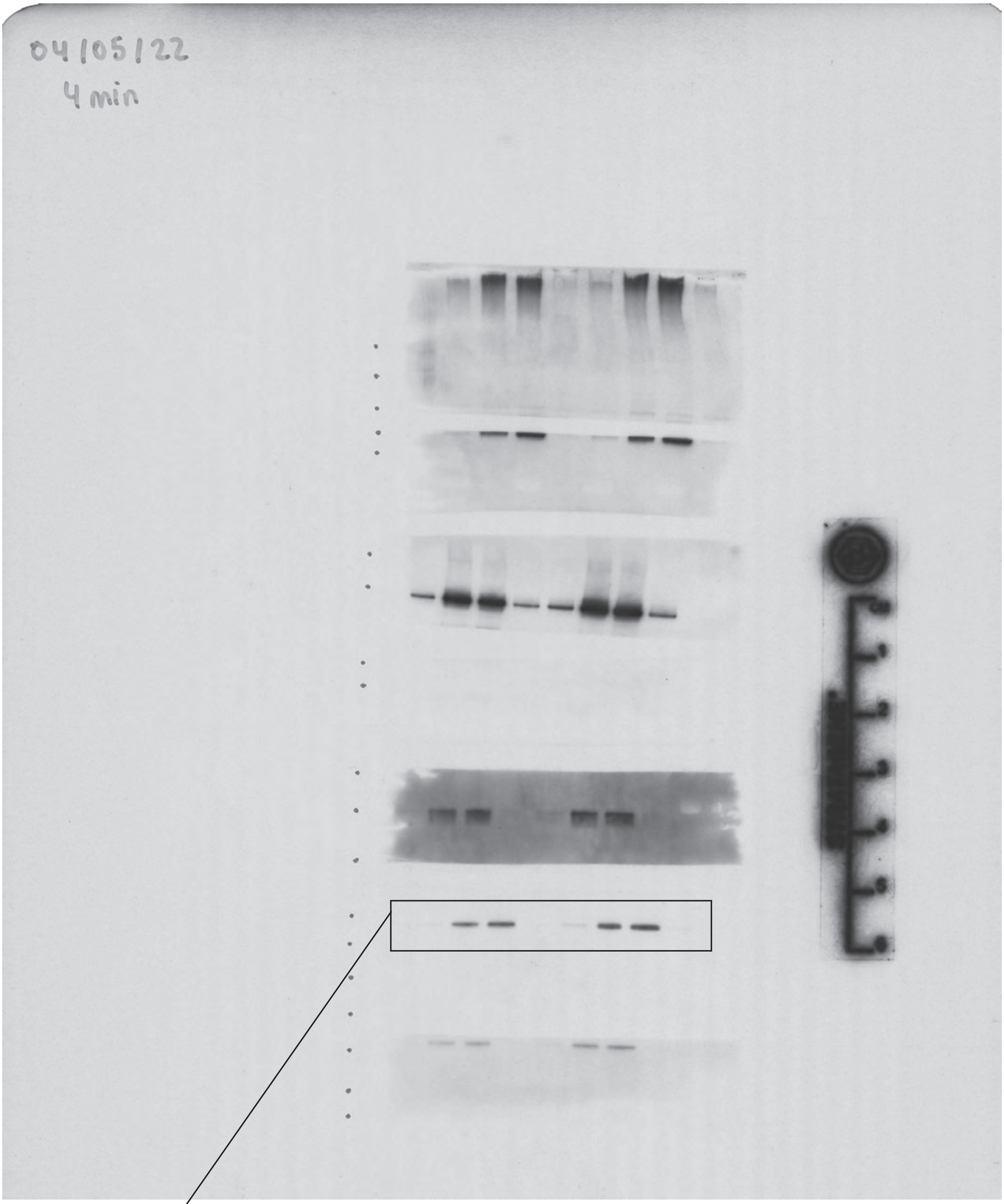

Cropped area for Figure 2-figure supplement 3A
Psf1

Cropped area for Figure 2-figure supplement 3B
Mcm7

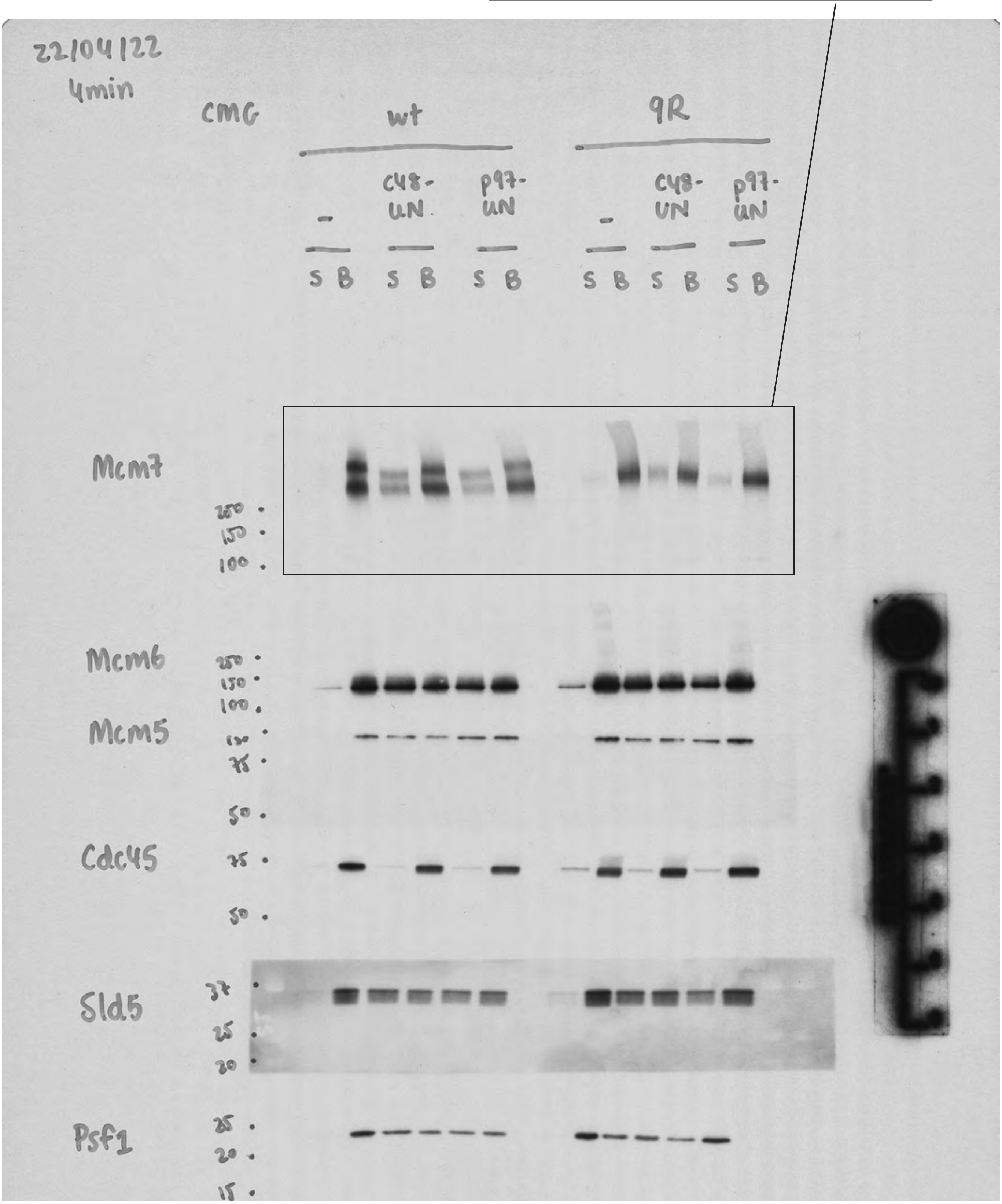

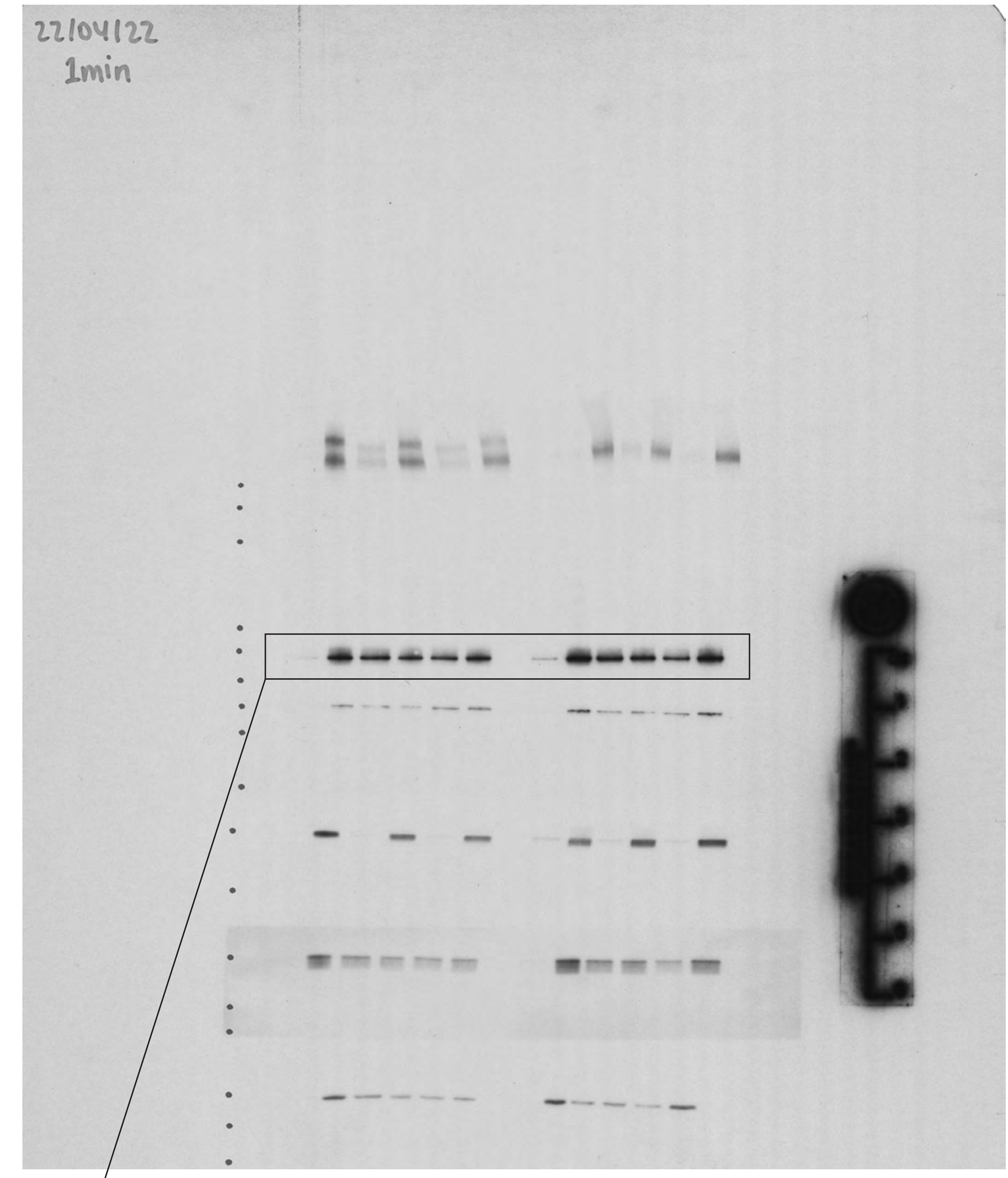

Cropped area for Figure 2-figure supplement 3B
Mcm6

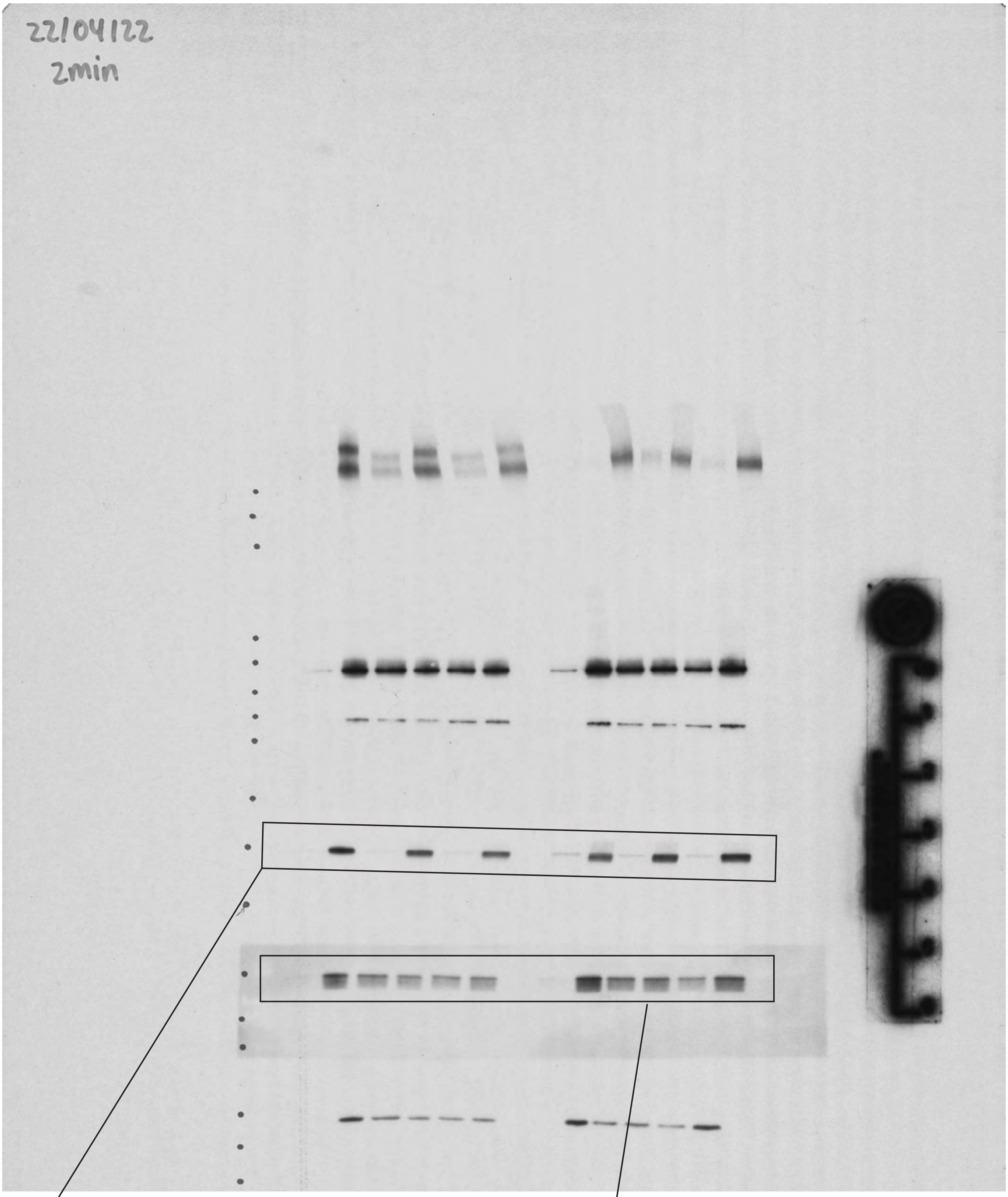

Cropped area for Figure 2-figure supplement 3B
Cdc45

Cropped area for Figure 2-figure supplement 3B
Sld5
