# Supplementary material for: Multiple UBX proteins reduce the ubiquitin threshold of the mammalian p97-UFD1-NPL4 unfoldase
Source: eLife. 2022 Aug 3;11:e76763. doi: 10.7554/eLife.76763 (PMC9377798; doi:10.7554/eLife.76763)
Supplement: Figure 3—source data 1. [file elife-76763-fig3-data1.pdf]

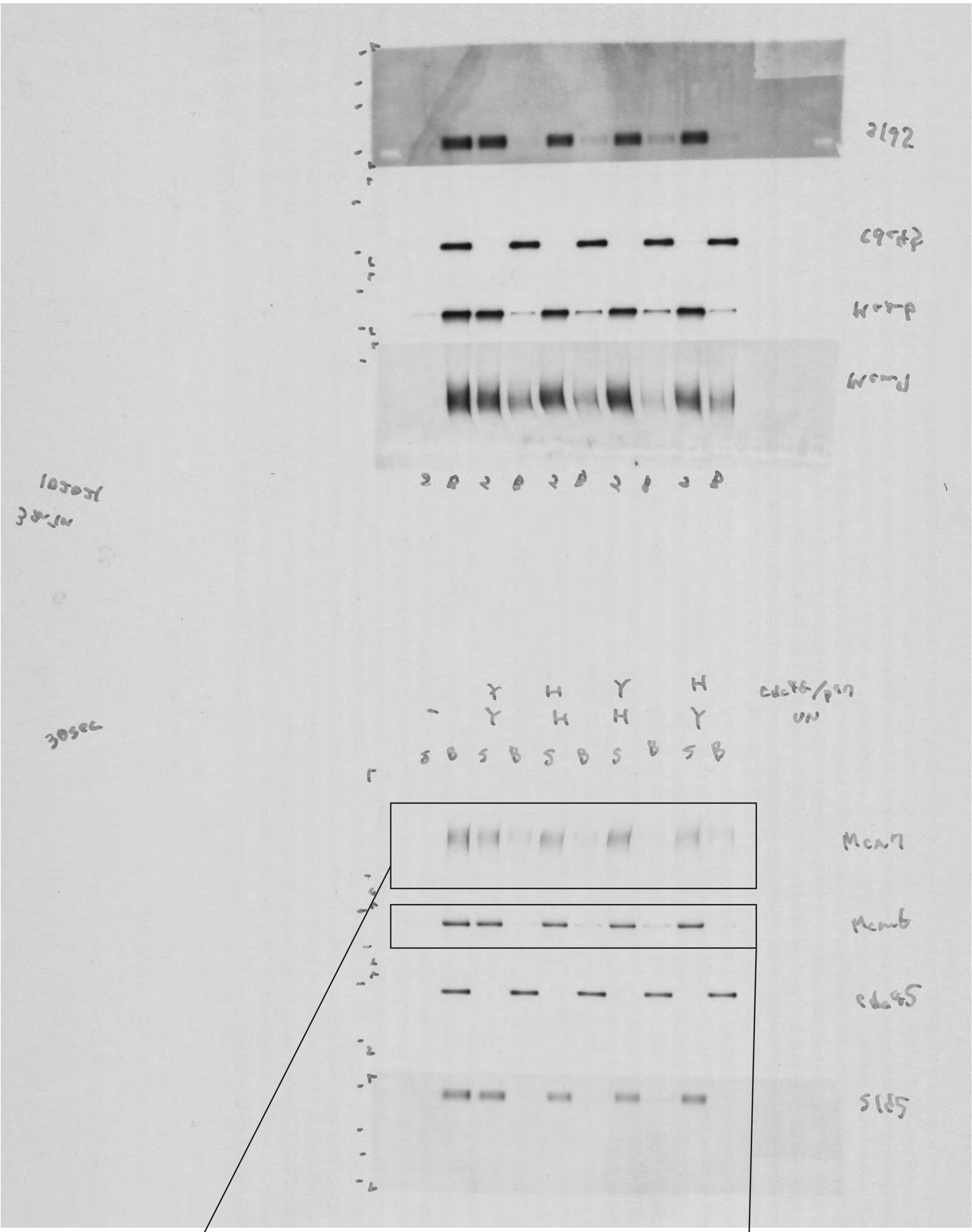

Cropped area for Figure 3A
Mcm7

Cropped area for Figure 3A
Mcm6

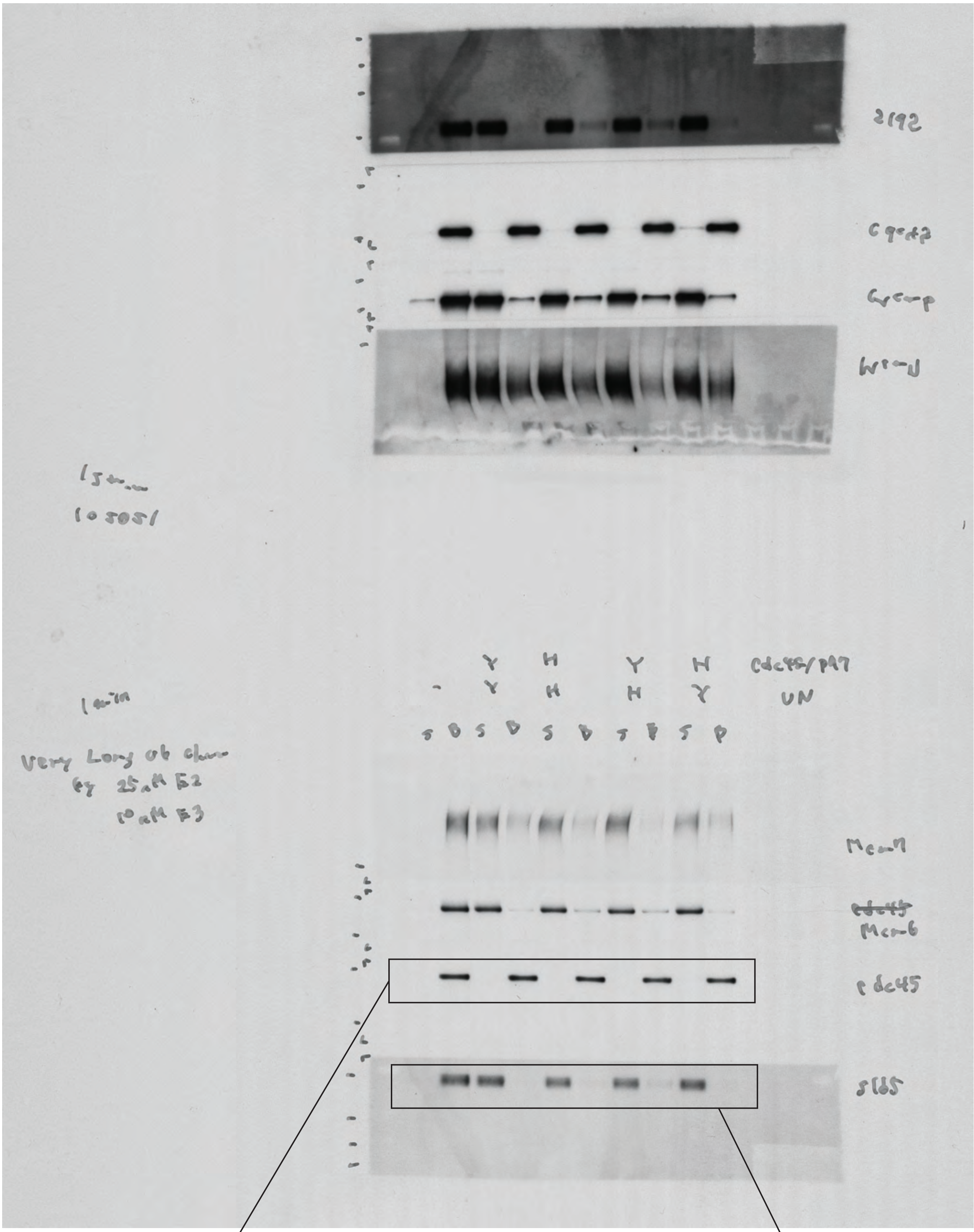

Cropped area for Figure 3A
Cdc45

Cropped area for Figure 3A
Sld5

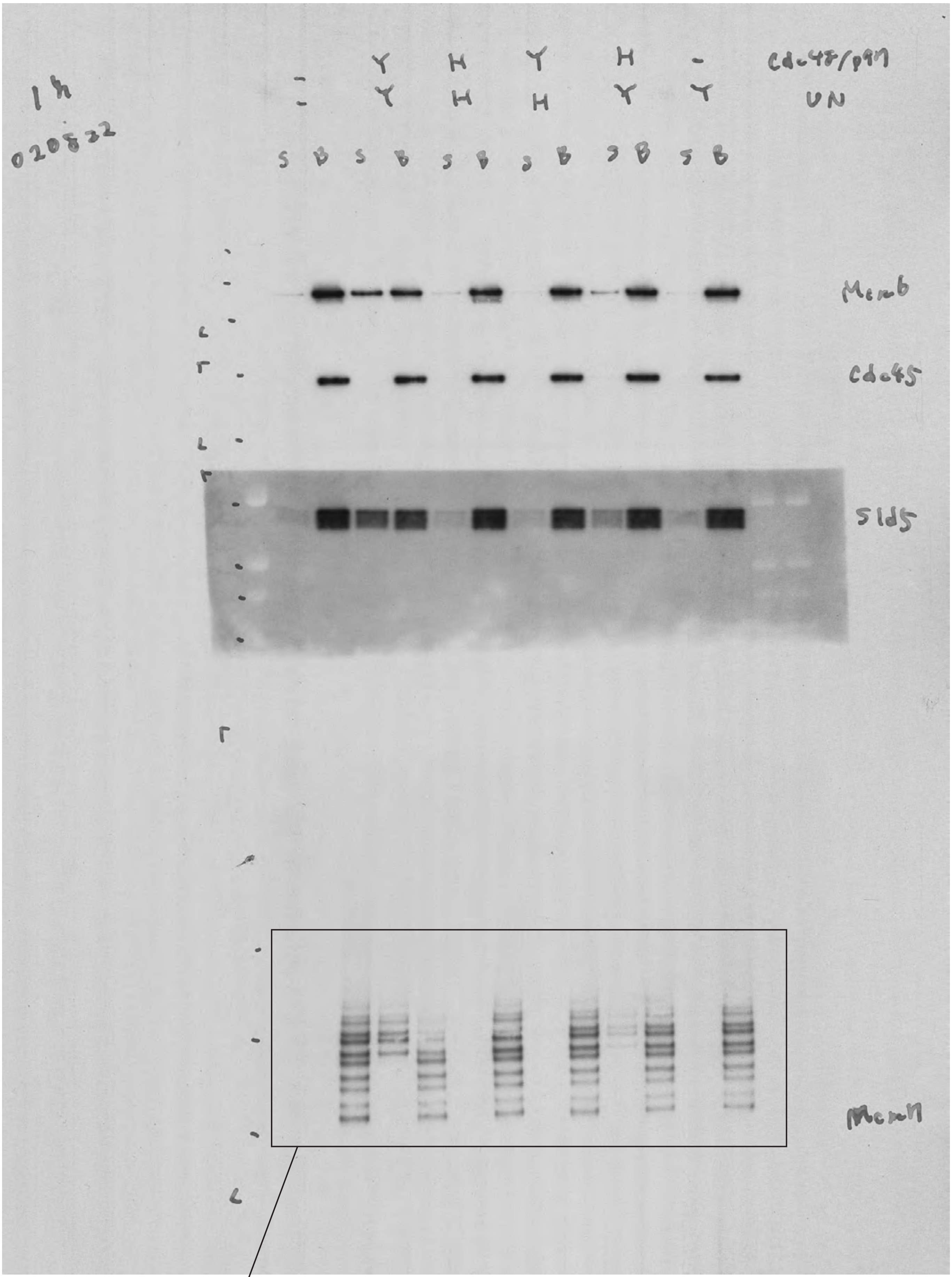

Cropped area for Figure 3B
Mcm7

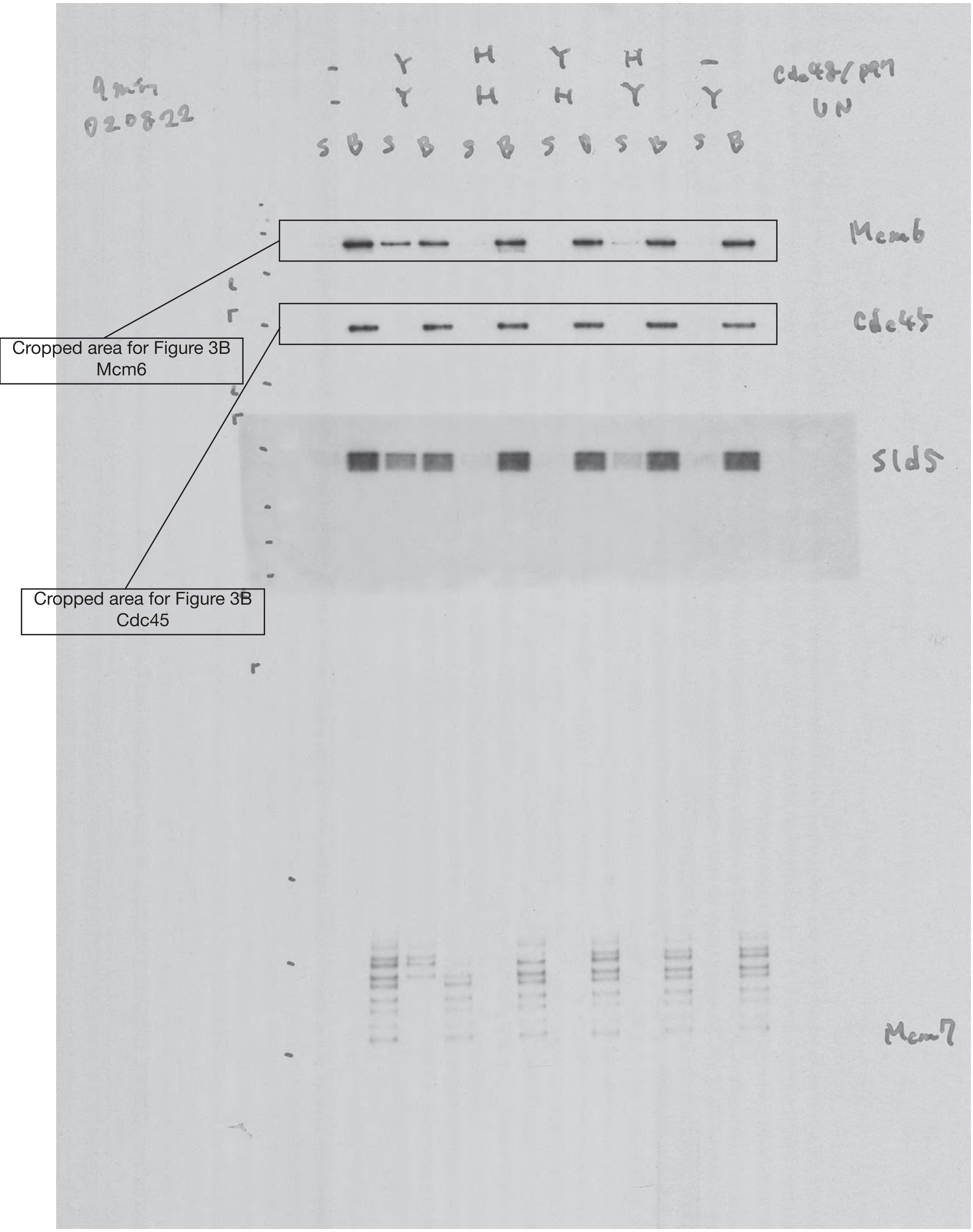
Cdc48/p97
UN
S B S B S B S B S B S B
Mcm6
Cdc45
Sld5
Mcm7
Cropped area for Figure 3B Mcm6
Cropped area for Figure 3B Cdc45

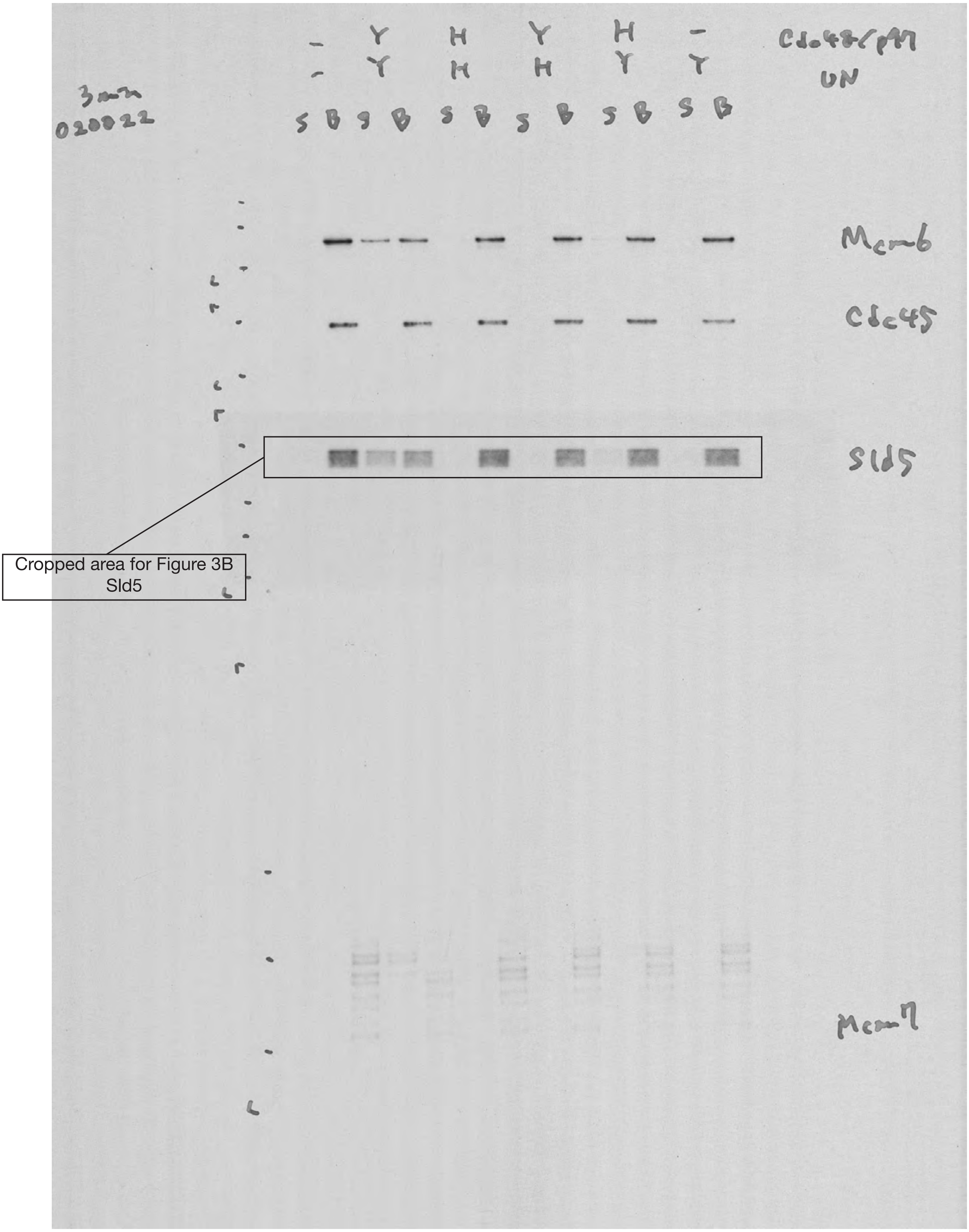
Cdc48/pM
UN
Mcm6
Cdc45
Sld5
Mcm7
Cropped area for Figure 3B
Sld5
