# Supplementary material for: Multiple UBX proteins reduce the ubiquitin threshold of the mammalian p97-UFD1-NPL4 unfoldase
Source: eLife. 2022 Aug 3;11:e76763. doi: 10.7554/eLife.76763 (PMC9377798; doi:10.7554/eLife.76763)
Supplement: Figure 3—figure supplement 2—source data 1. [file elife-76763-fig3-figsupp2-data1.pdf]

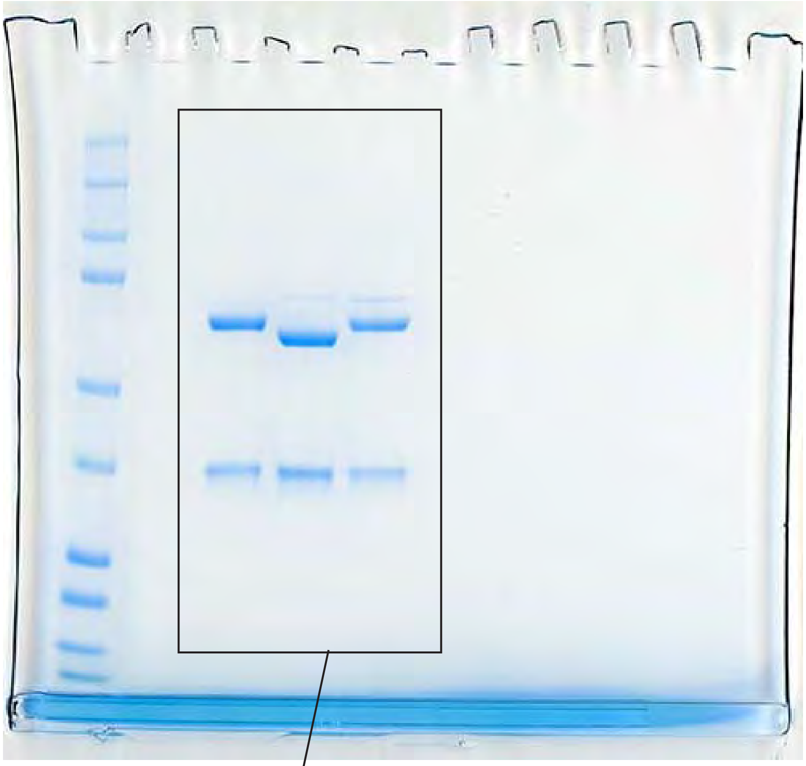

Cropped area for Figure 3-figure supplement 2B

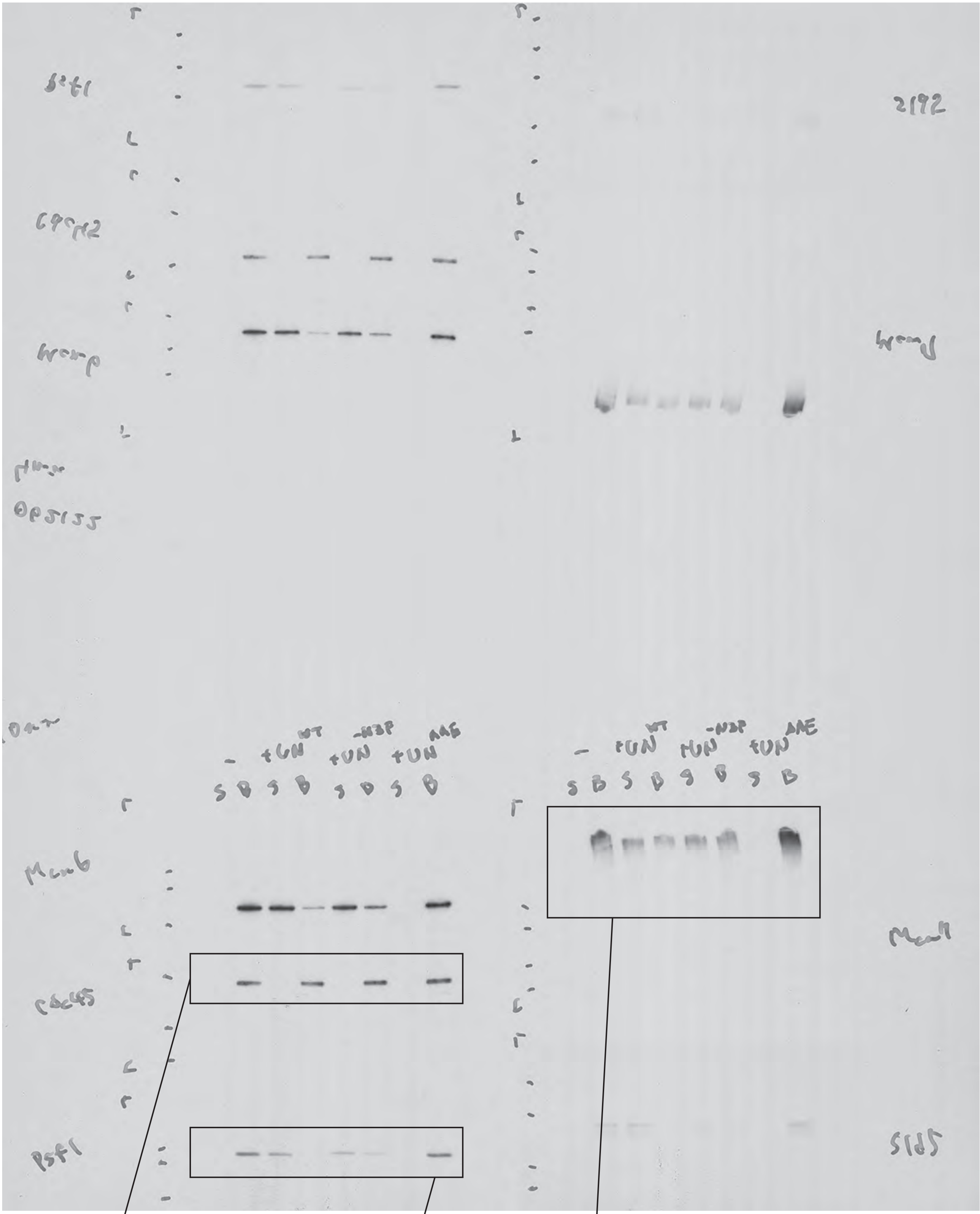

Cropped area for Cdc45

Cropped area for Mcm7

Cropped area for Psf1

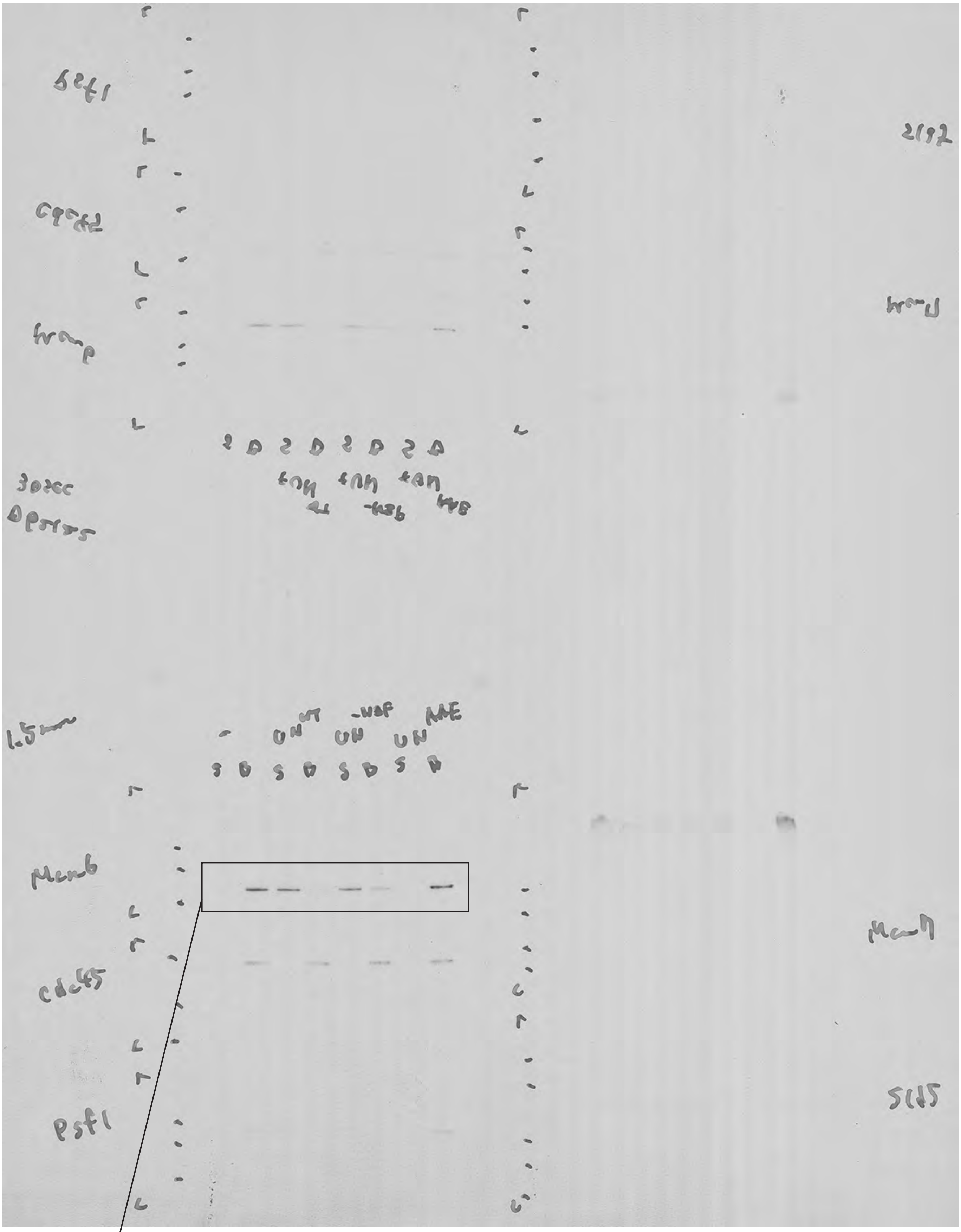

Cropped area for Mcm6

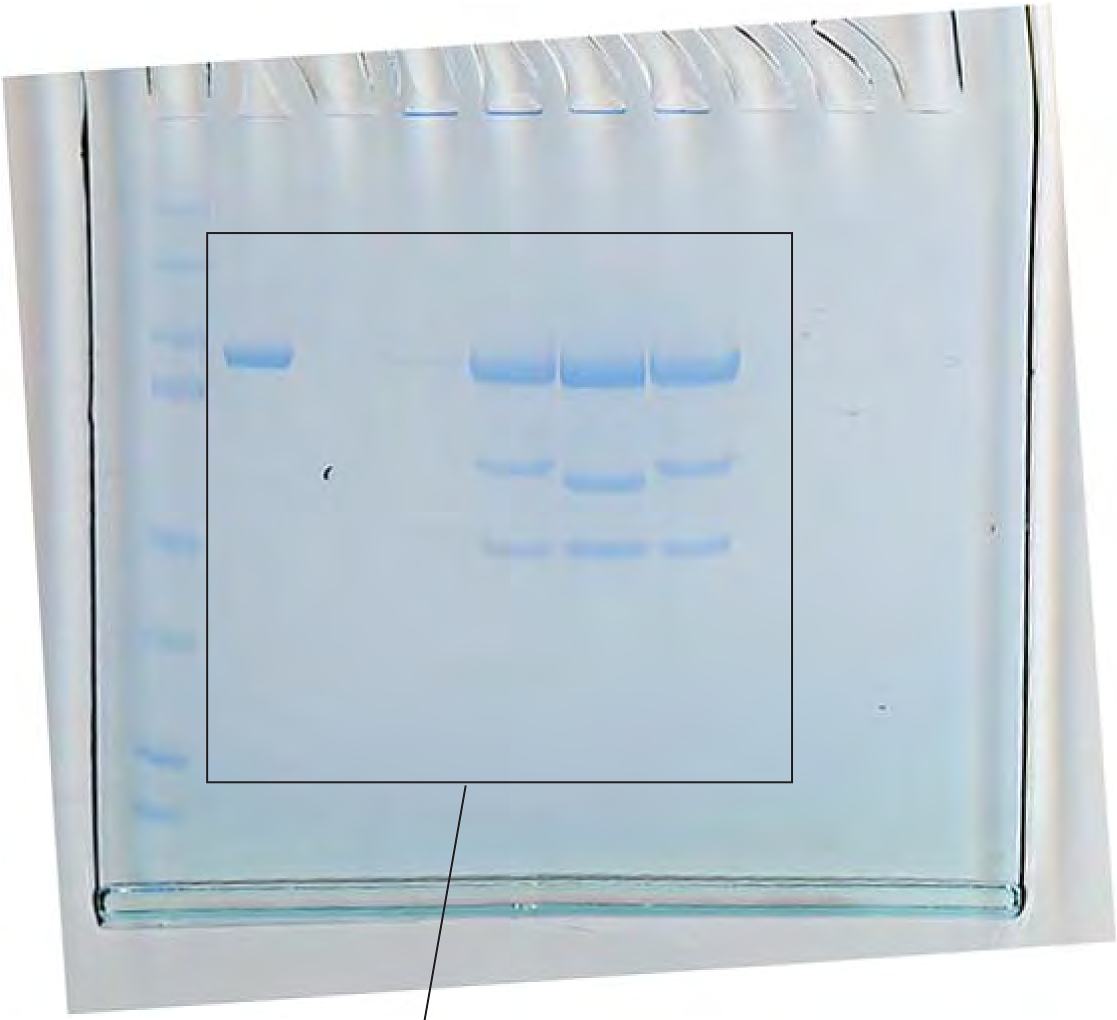

Cropped region for Figure 3-figure supplement 2D

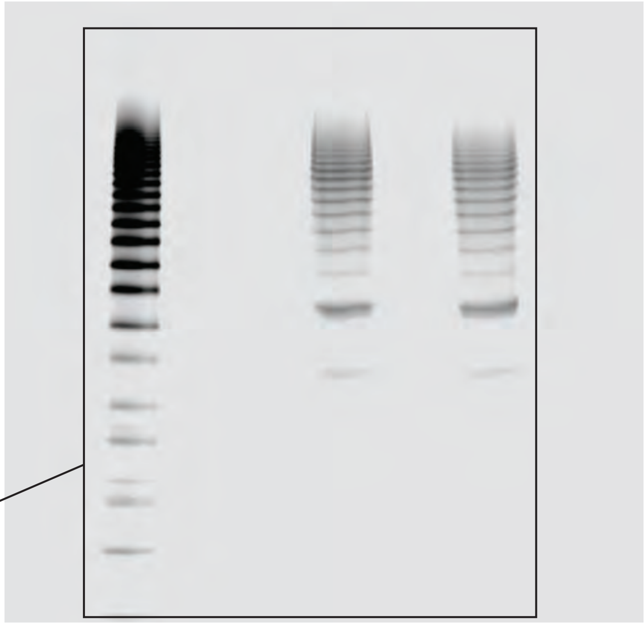

Cropped area for Figure 3-figure supplement 2E
K48-linked ubiquitin chains
(IR Dye 800CW), 800 nm channel

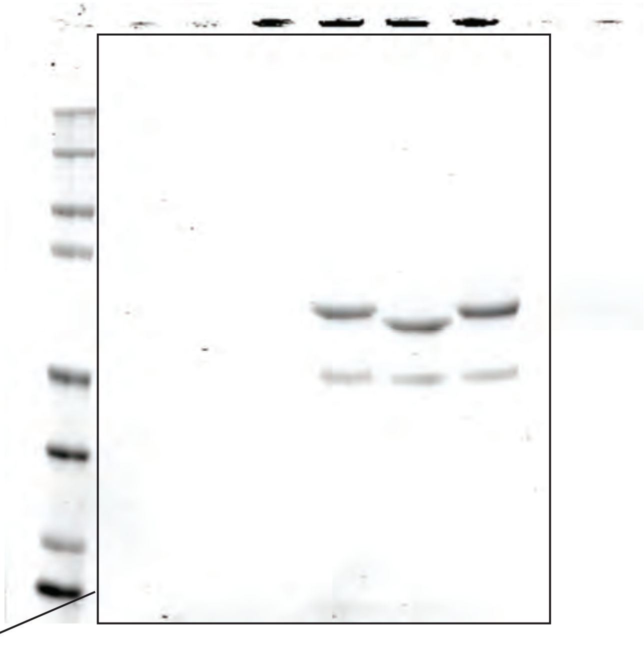

Cropped area for Figure 3-figure supplement 2E
Coomassie blue staining,
700 nm channel
