# Supplementary material for: Multiple UBX proteins reduce the ubiquitin threshold of the mammalian p97-UFD1-NPL4 unfoldase
Source: eLife. 2022 Aug 3;11:e76763. doi: 10.7554/eLife.76763 (PMC9377798; doi:10.7554/eLife.76763)
Supplement: Figure 4—source data 1. [file elife-76763-fig4-data1.pdf]

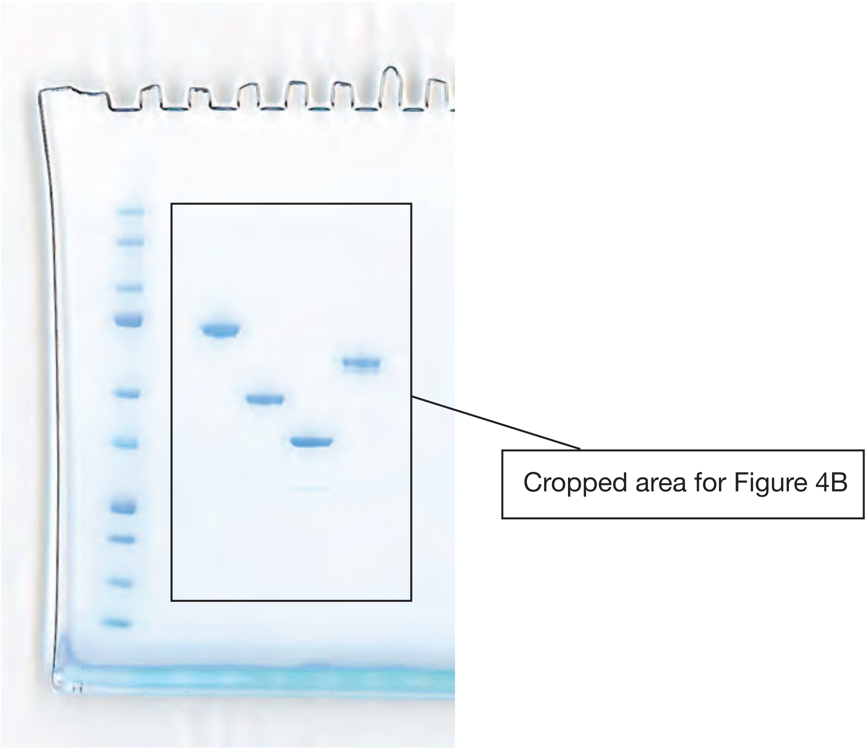
Cropped area for Figure 4B

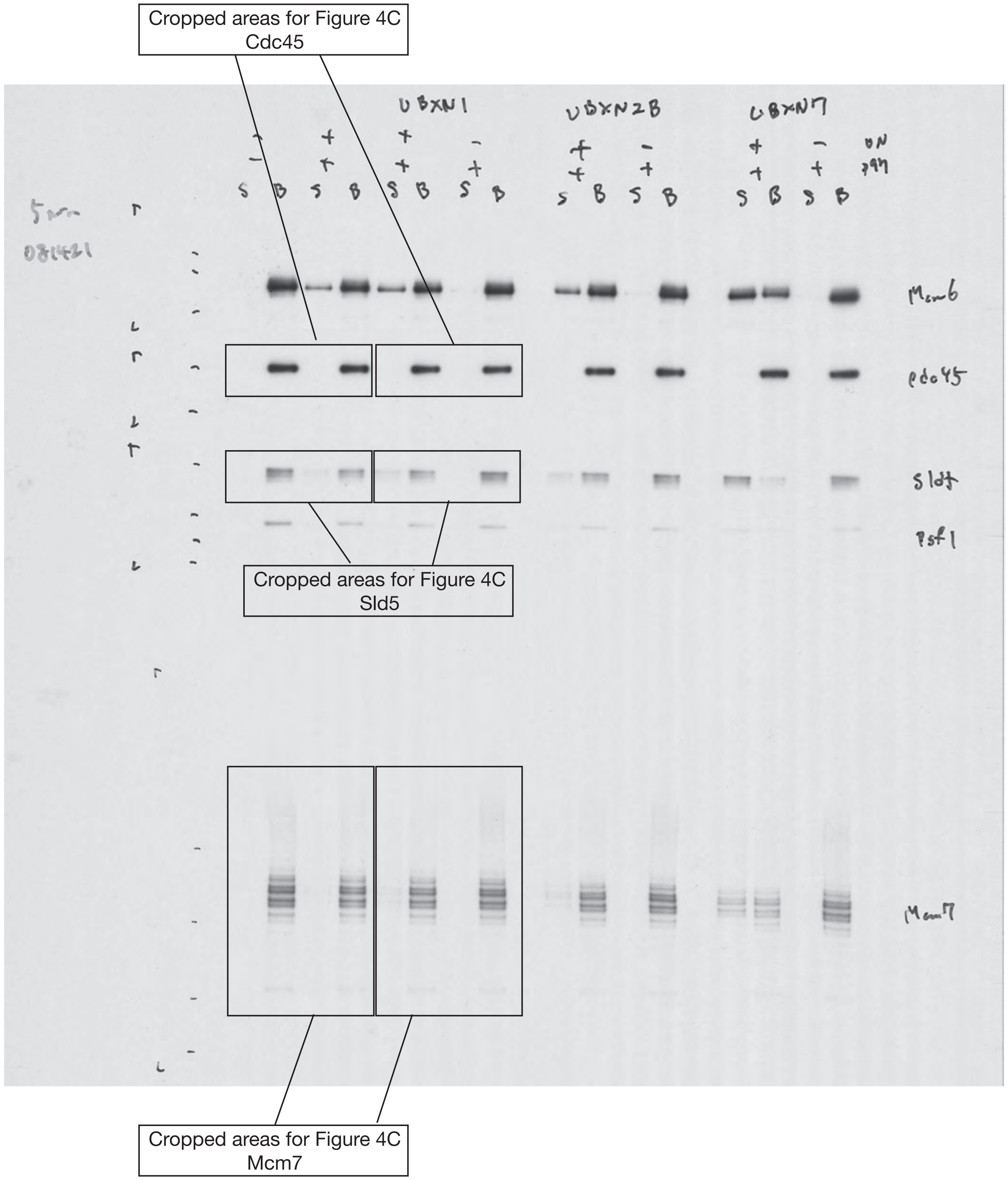
Cropped areas for Figure 4C
Cdc45
Cropped areas for Figure 4C
Sld5
Cropped areas for Figure 4C
Mcm7

Cropped areas for Figure 4C
Mcm6

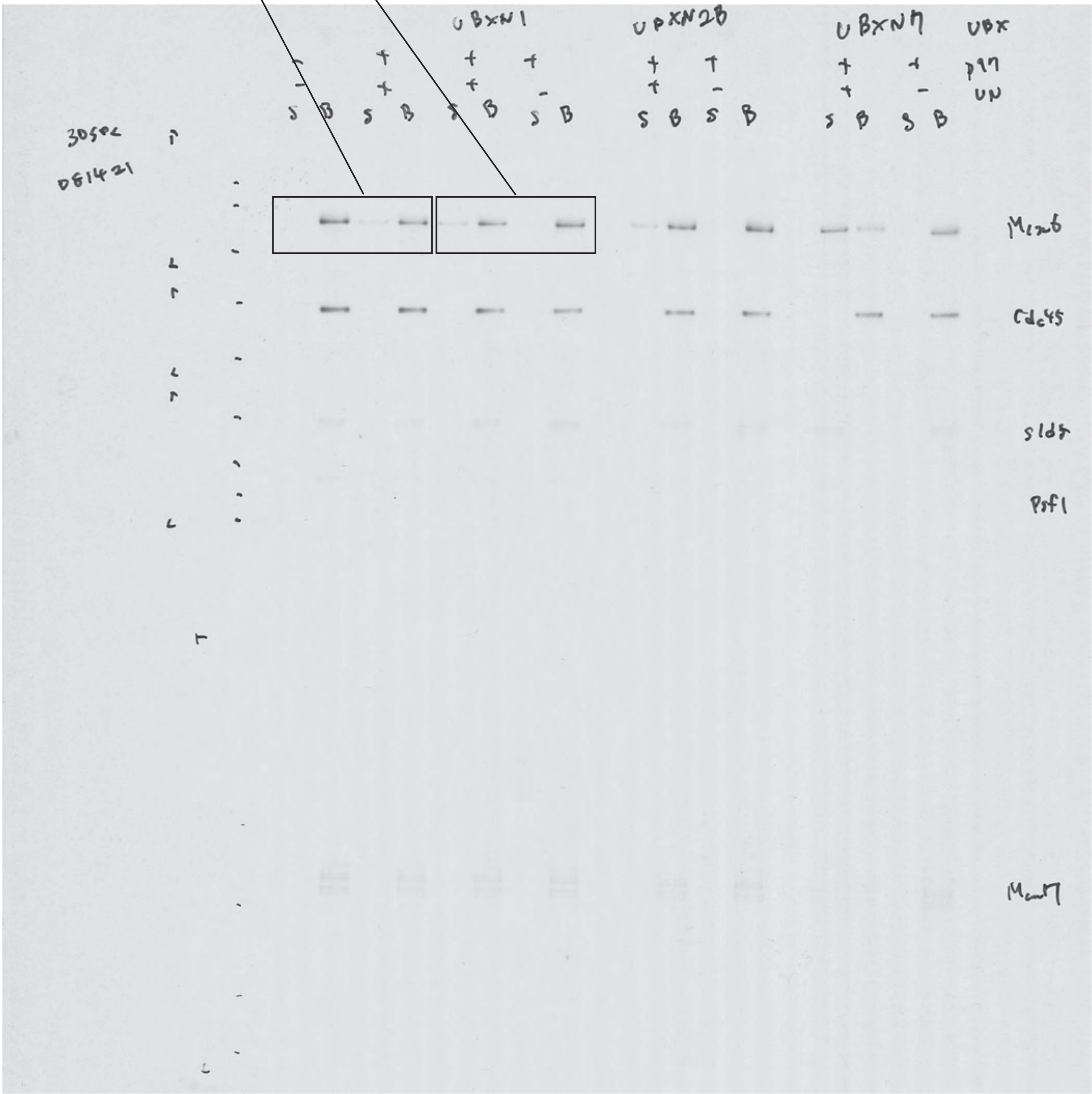

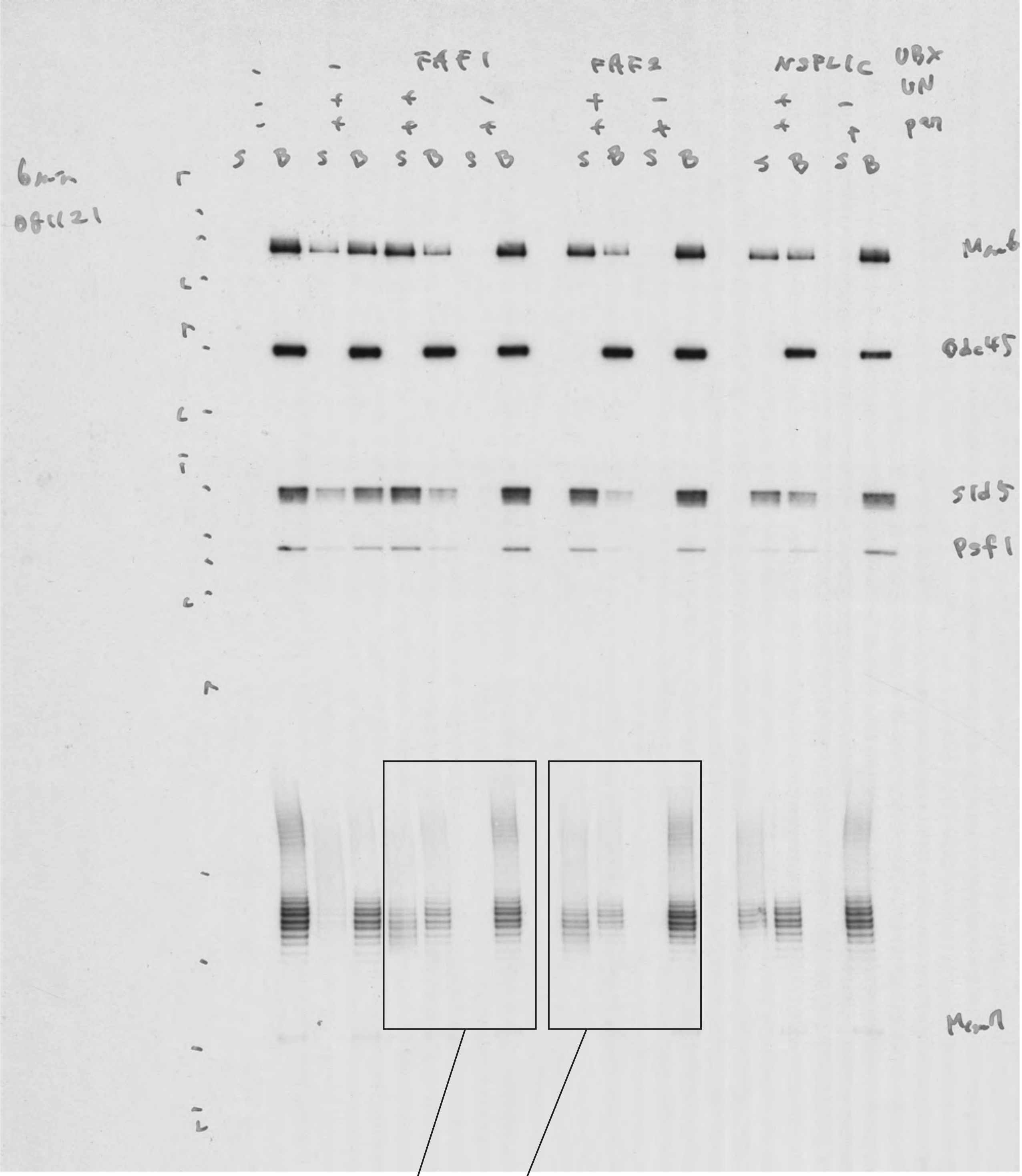

Cropped areas for Figure 4D
Mcm7 (FAF1 & FAF2 samples)

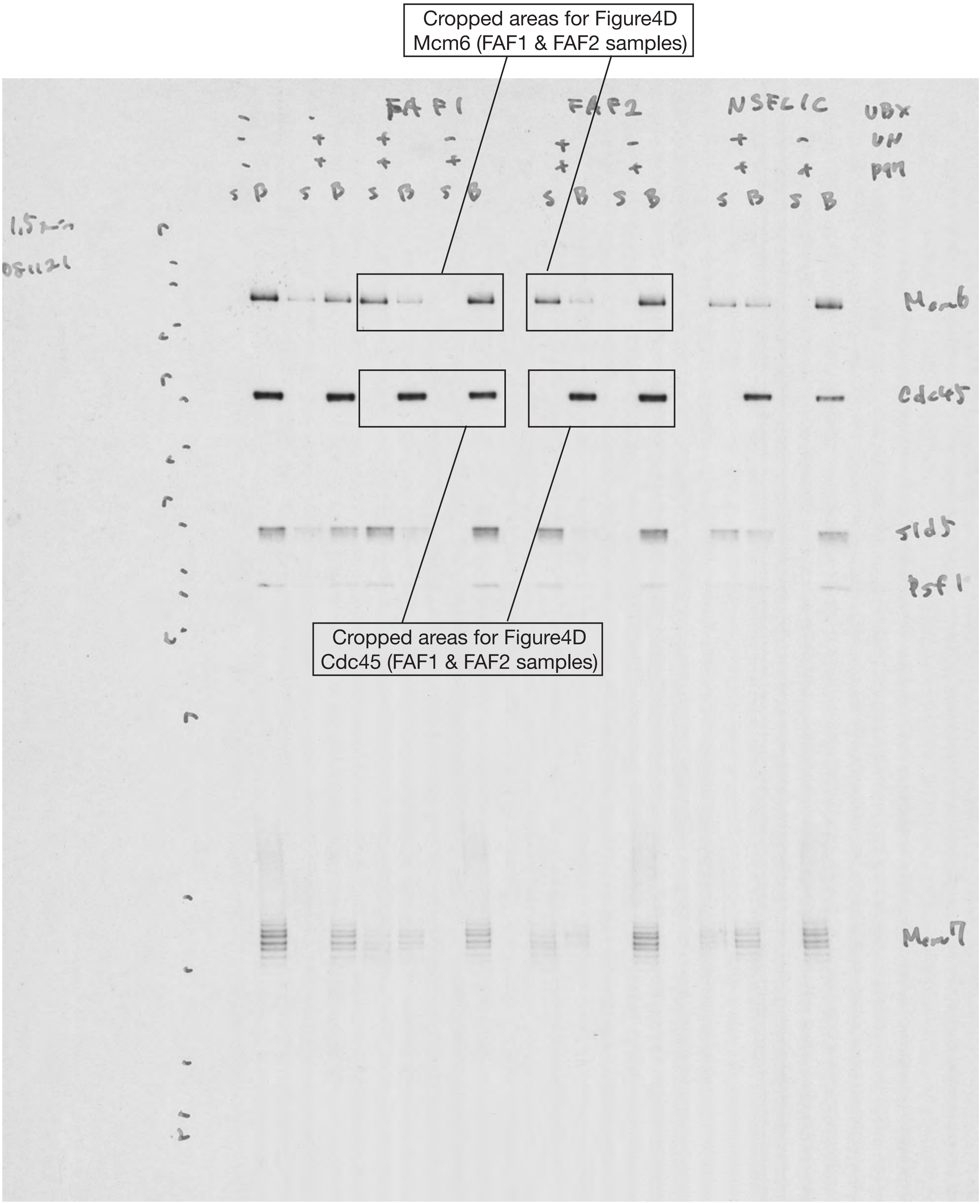
Cropped areas for Figure4D
Mcm6 (FAF1 & FAF2 samples)
Cropped areas for Figure4D
Cdc45 (FAF1 & FAF2 samples)
FAF1
FAF2
NSFL1C
UBX
UN
p97
Mcm6
Cdc45
Sld5
Psf1
Mcm7

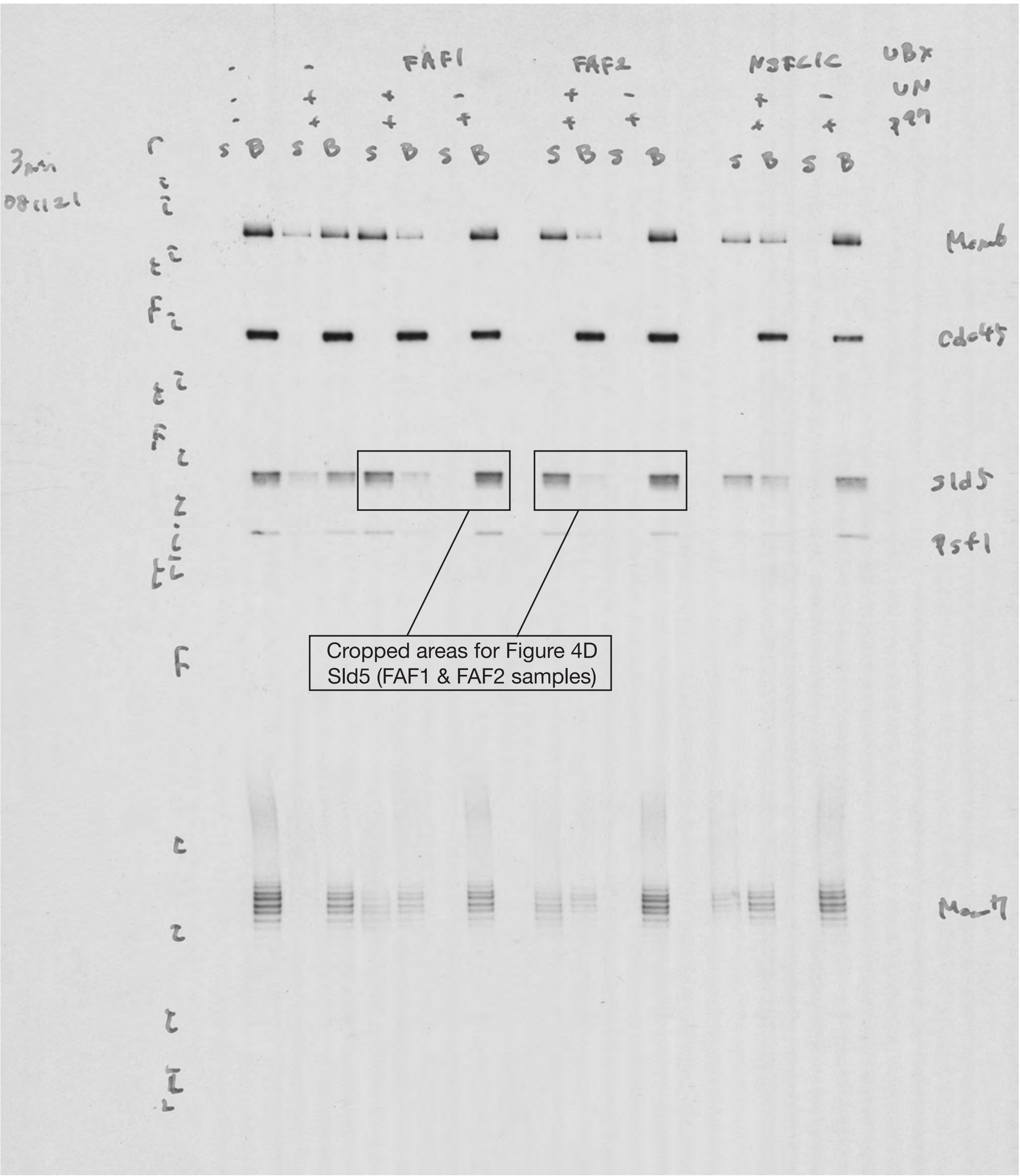
FAF1
FAF2
UBX
UN
p97
Mcm6
Cdc45
Sld5
Psf1
Mcm7
Cropped areas for Figure 4D
Sld5 (FAF1 & FAF2 samples)

Cropped area for Figure 4D
Cdc45 (UBXN7 sample)

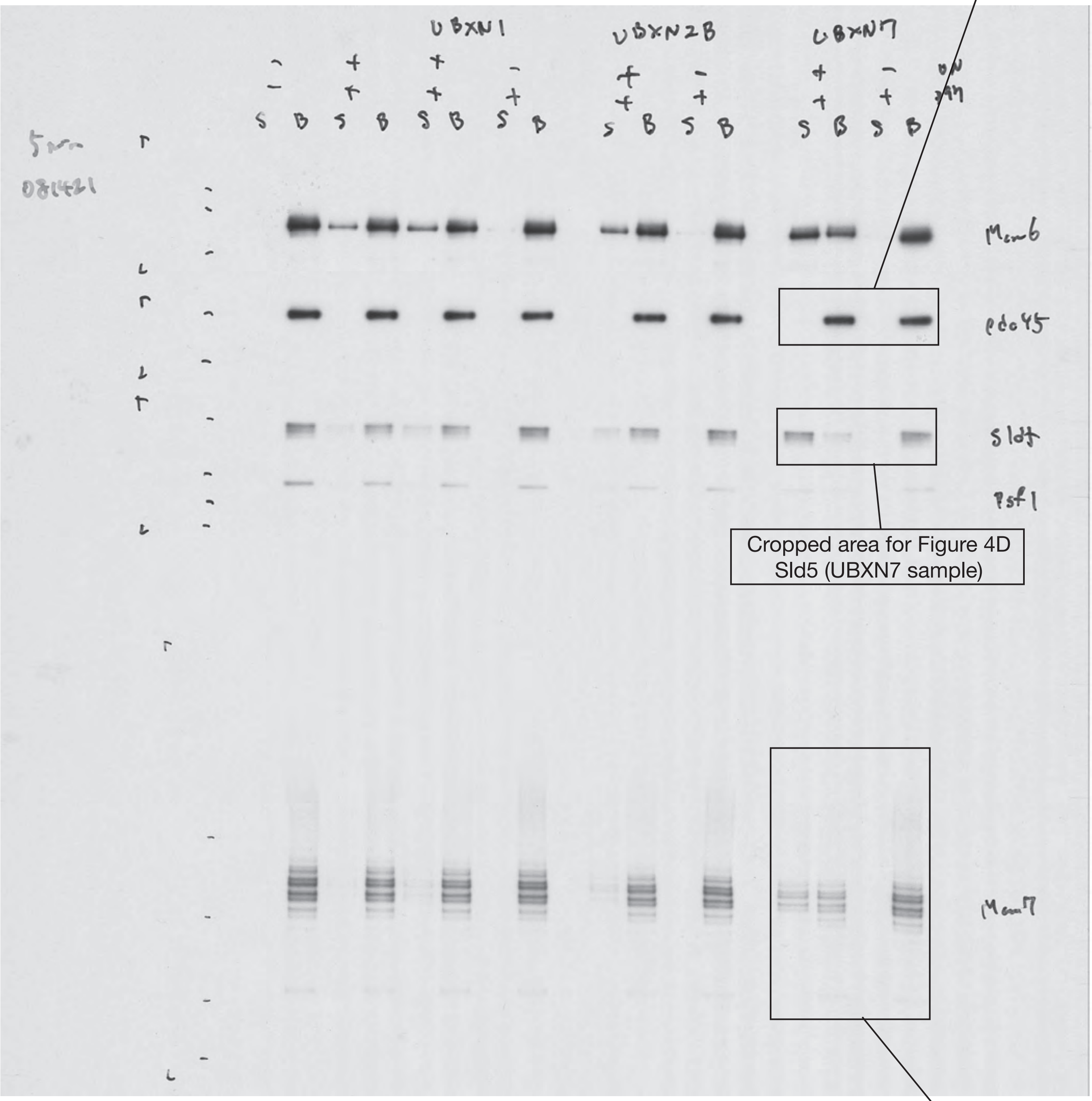

Cropped area for Figure 4D
Sld5 (UBXN7 sample)

Cropped area for Figure 4D
Mcm7 (UBXN7 sample)

Cropped area for Figure 4D
Mcm6 (UBXN7 sample)

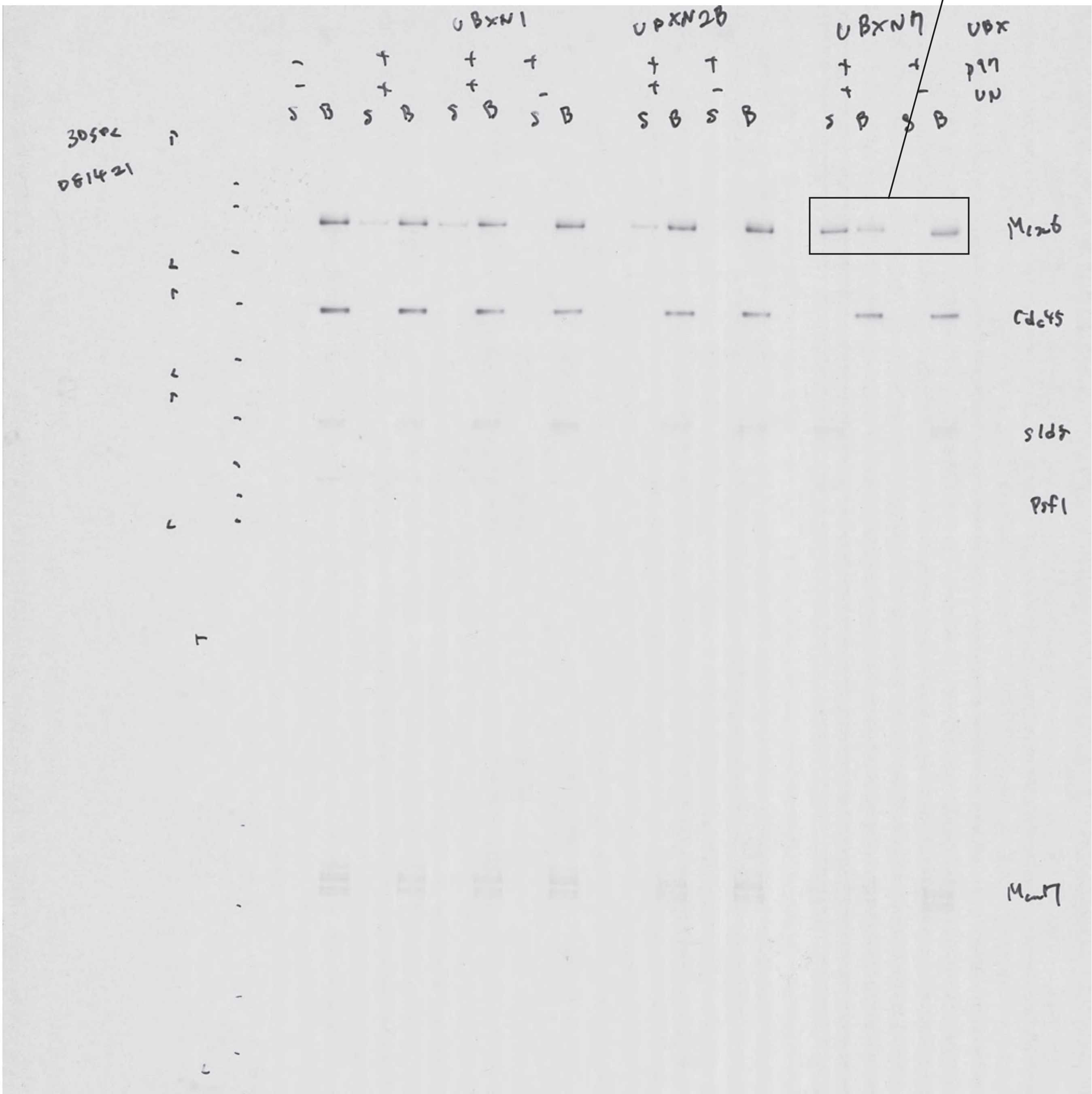

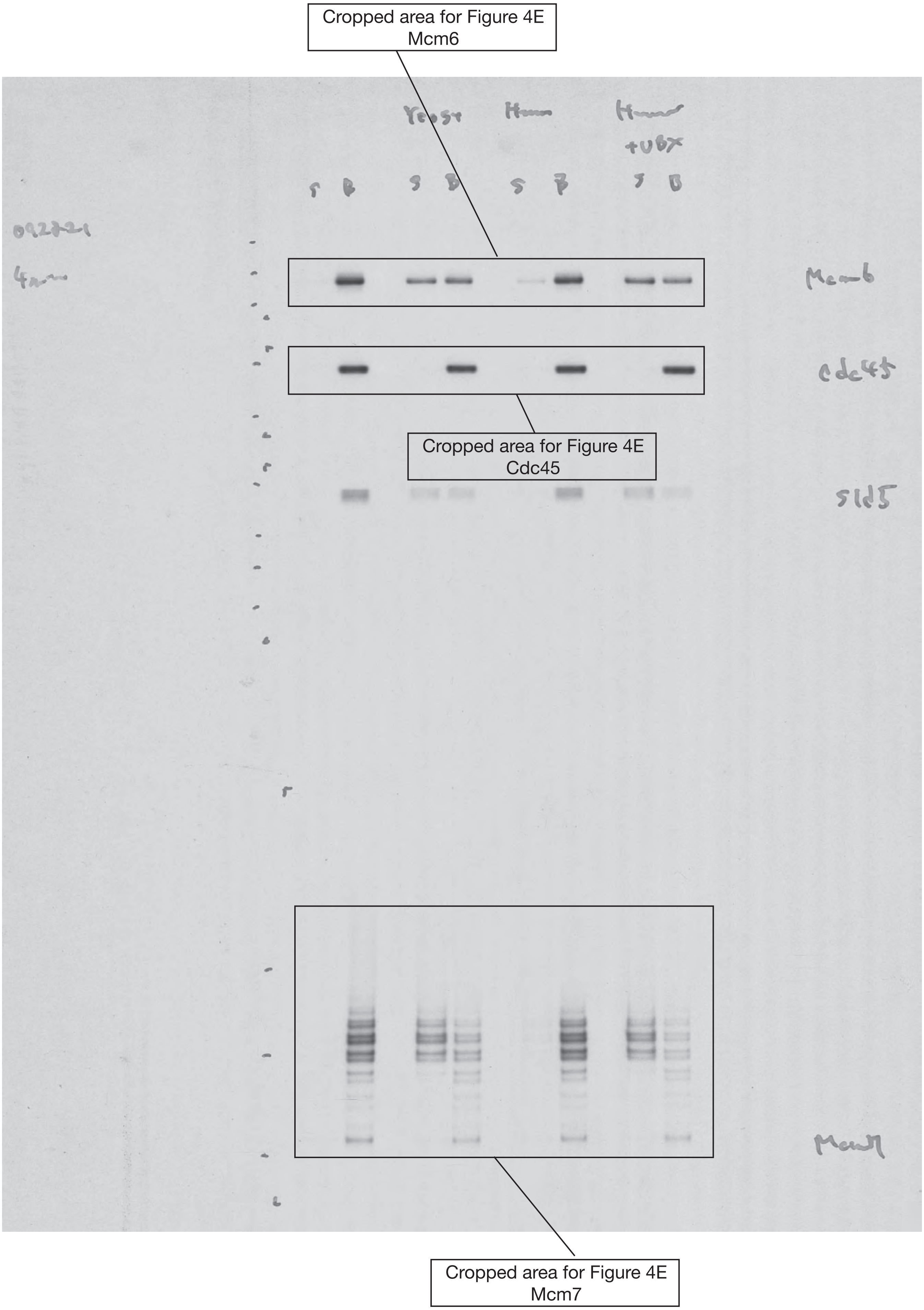
Cropped area for Figure 4E
Mcm6
Yeast
+UBX
S B S B S B S B
Mcm6
Cdc45
Cropped area for Figure 4E
Cdc45
Sld5
Mcm7
Cropped area for Figure 4E
Mcm7

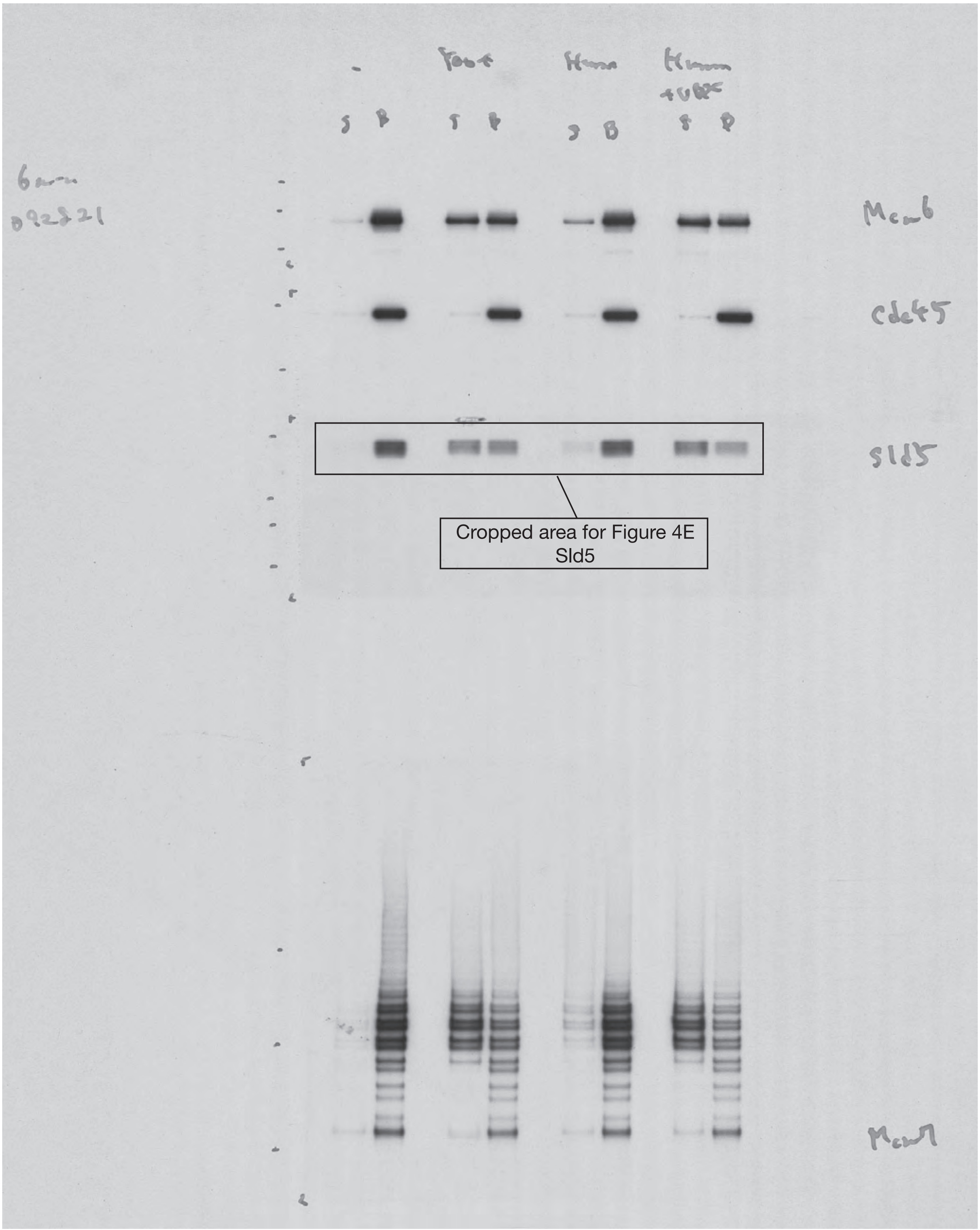
Mcm6
Cdc45
Sld5
Cropped area for Figure 4E
Sld5
Mcm7
