# Supplementary figures and images for: Multiple UBX proteins reduce the ubiquitin threshold of the mammalian p97-UFD1-NPL4 unfoldase
Source: eLife. 2022 Aug 3;11:e76763. doi: 10.7554/eLife.76763 (PMC9377798; doi:10.7554/eLife.76763)

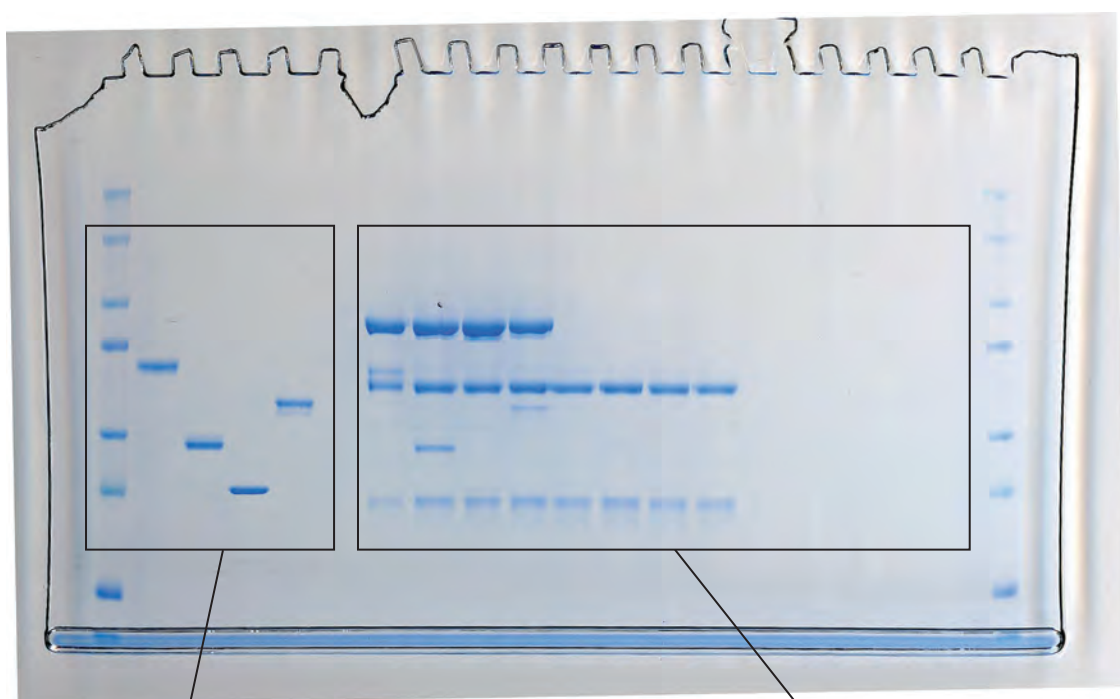

Cropped area for Figure 4-figure supplement 1A

Cropped area for Figure 4-figure supplement 1B

Supplement: Figure 4—figure supplement 1—source data 1. [file elife-76763-fig4-figsupp1-data1.pdf]

Cropped area for Figure 4-figure supplement 3  
Cdc45

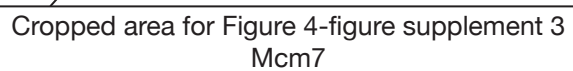

Supplement: Figure 4—figure supplement 3—source data 1. [file elife-76763-fig4-figsupp3-data1.pdf]
