# Supplementary material for: Multiple UBX proteins reduce the ubiquitin threshold of the mammalian p97-UFD1-NPL4 unfoldase
Source: eLife. 2022 Aug 3;11:e76763. doi: 10.7554/eLife.76763 (PMC9377798; doi:10.7554/eLife.76763)
Supplement: Figure 4—figure supplement 2—source data 1. [file elife-76763-fig4-figsupp2-data1.pdf]

Cropped area for Figure 4-figure supplement 2
Mcm7

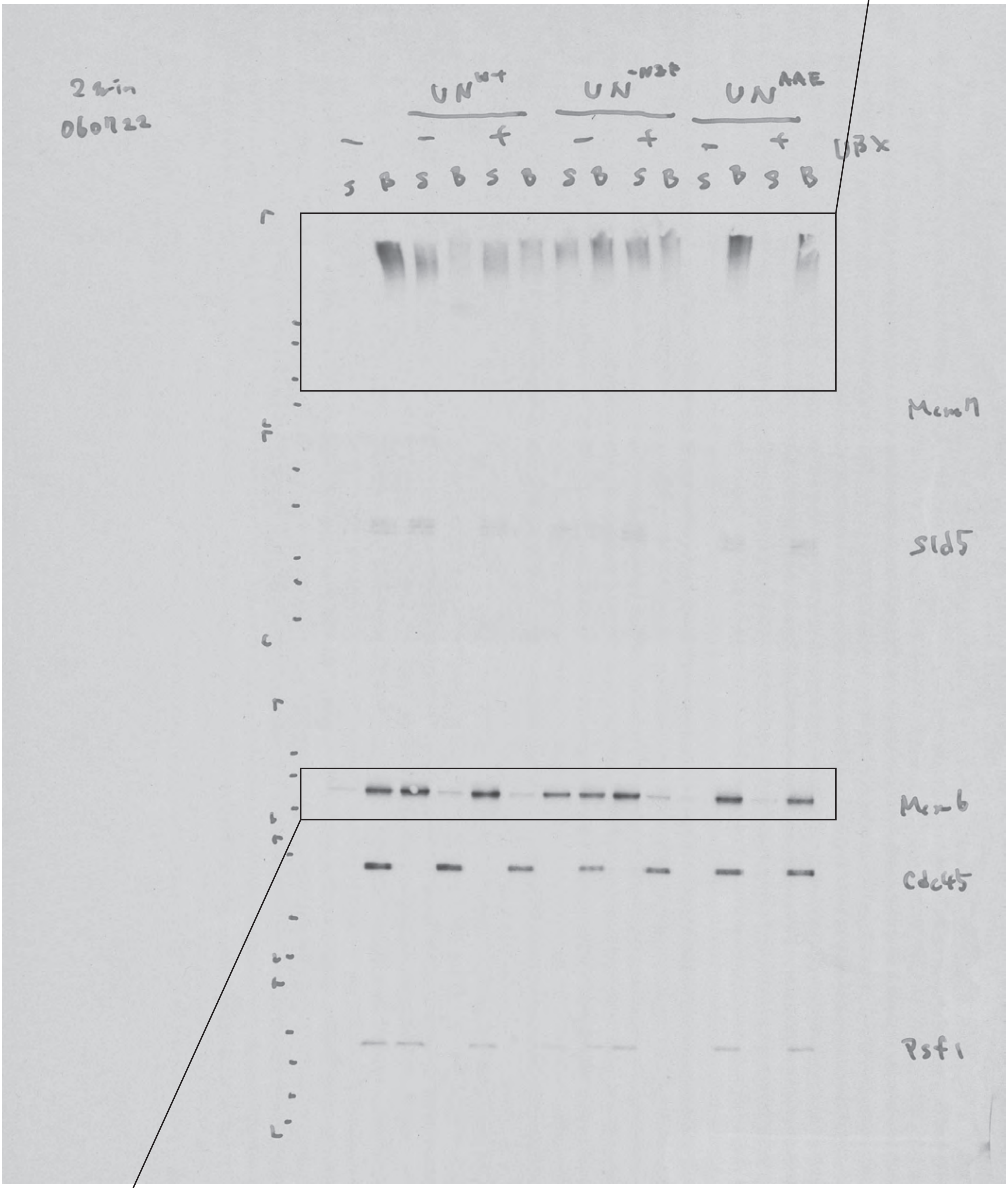

Cropped area for Figure 4-figure supplement 2
Mcm6

Cropped area for Figure 4-figure supplement 2
Sld5

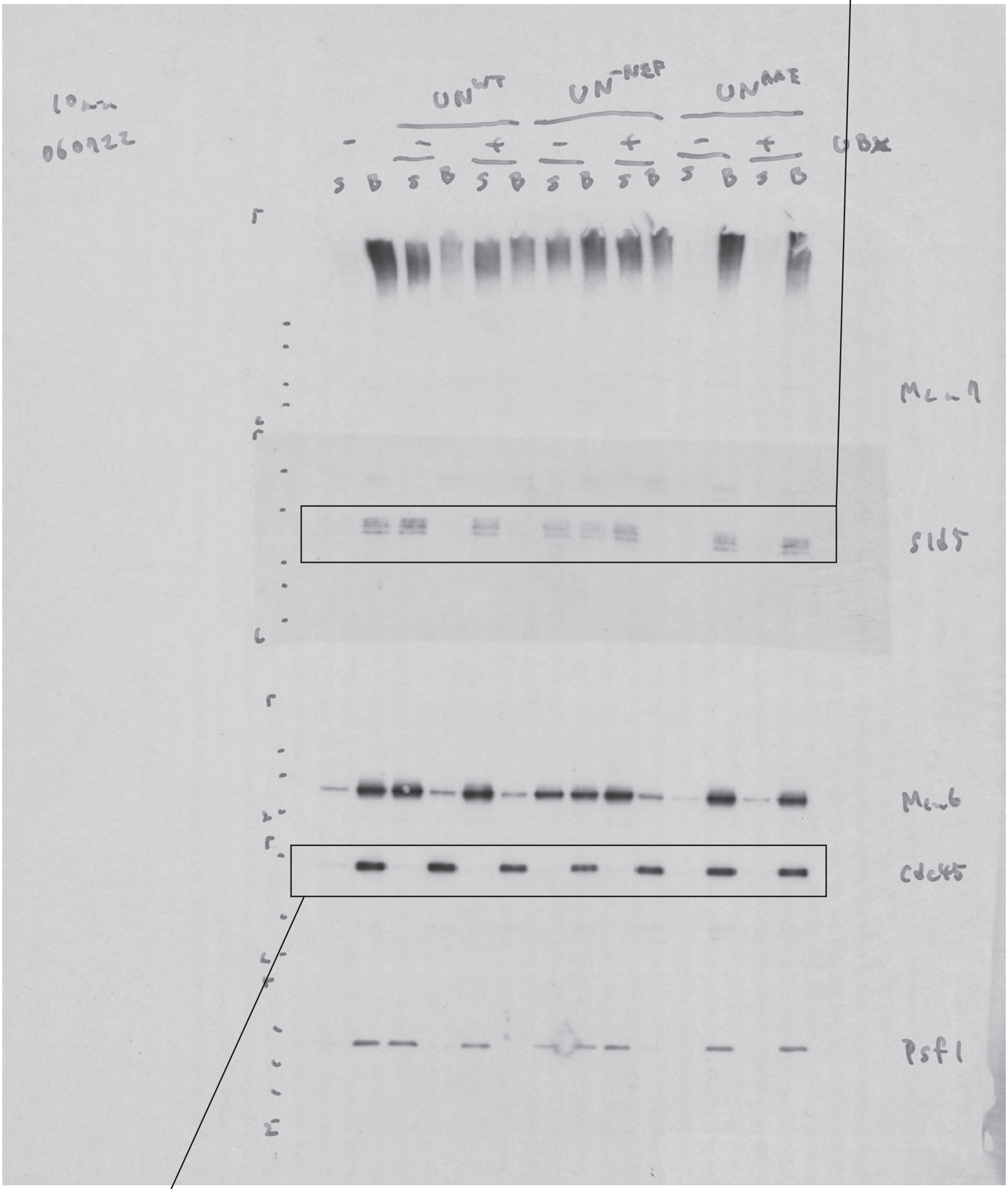

Cropped area for Figure 4-figure supplement 2
Cdc45
