# Supplementary material for: Multiple UBX proteins reduce the ubiquitin threshold of the mammalian p97-UFD1-NPL4 unfoldase
Source: eLife. 2022 Aug 3;11:e76763. doi: 10.7554/eLife.76763 (PMC9377798; doi:10.7554/eLife.76763)

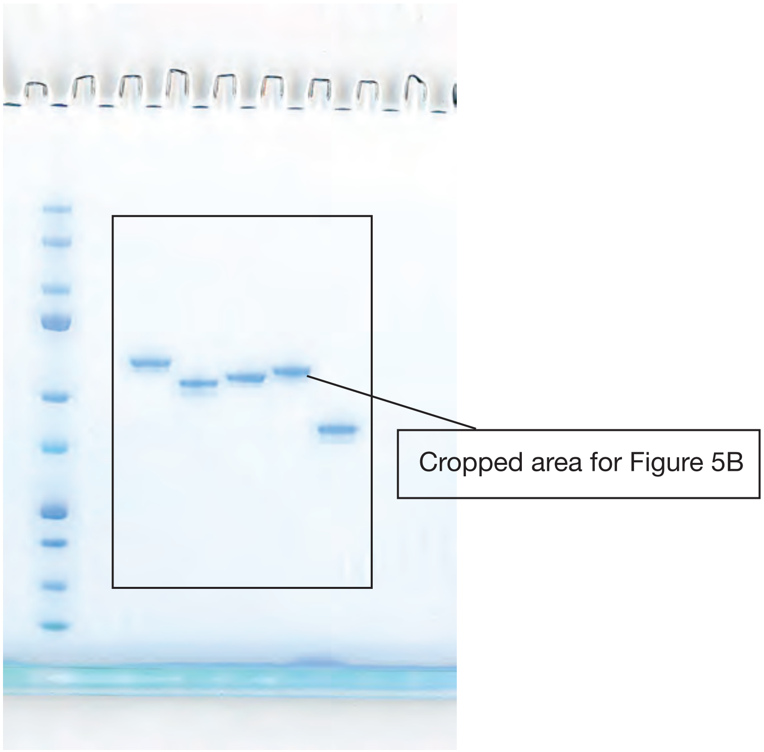
Cropped area for Figure 5B

Cropped area for Figure 5C
Cdc45

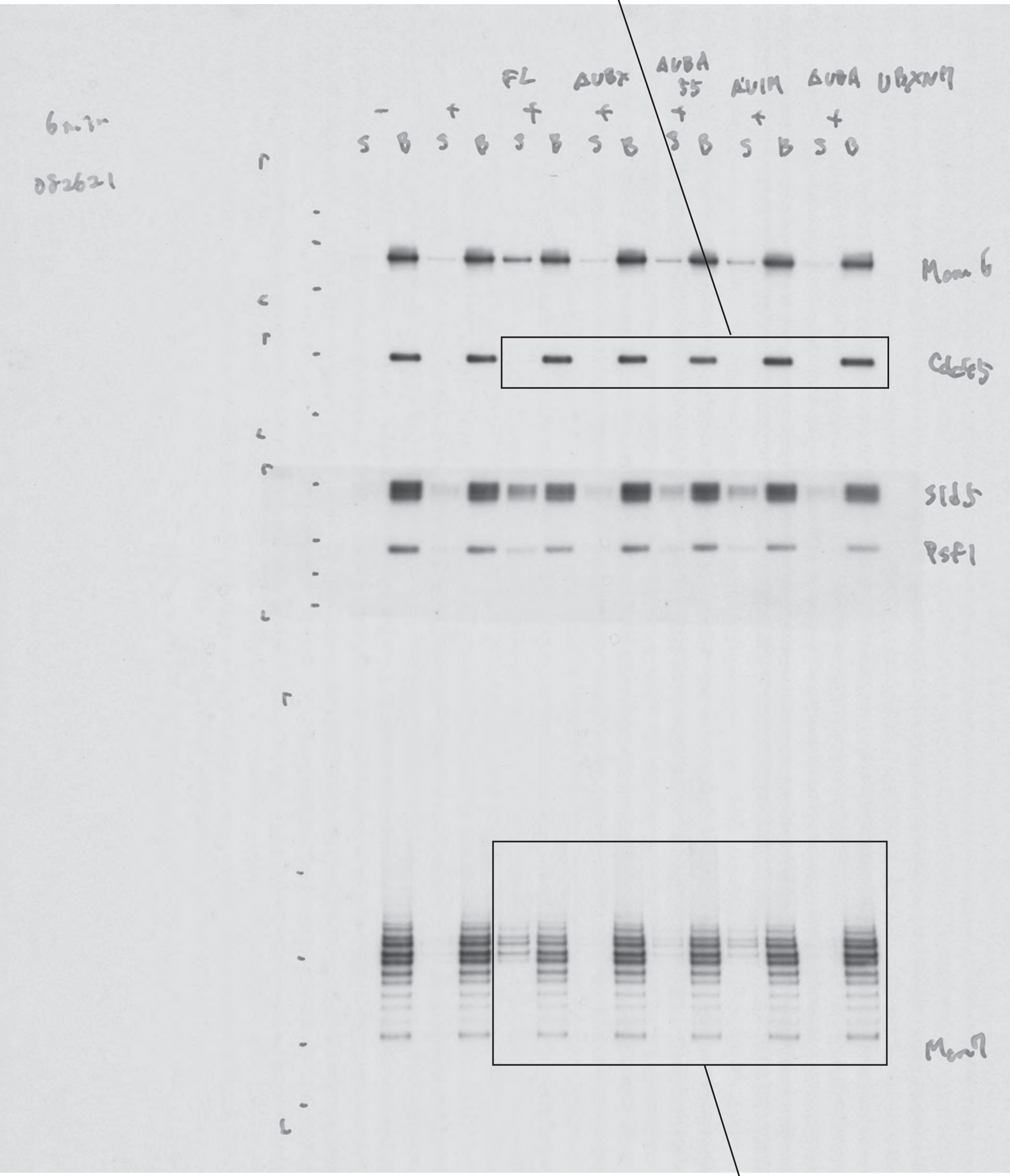

Cropped area for Figure 5C
Mcm7

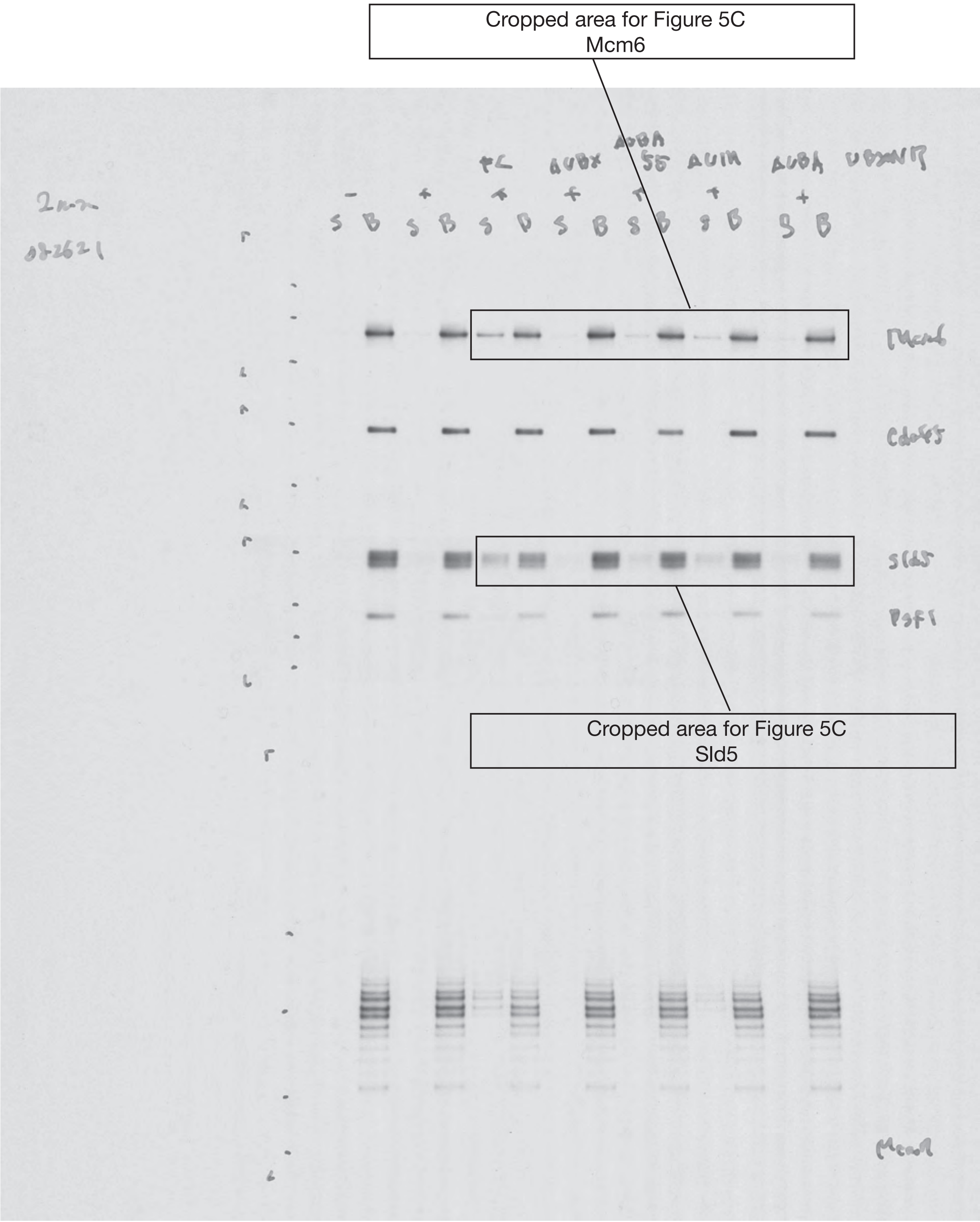
Cropped area for Figure 5C
Mcm6
Cropped area for Figure 5C
Sld5
Mcm6
Cdc45
Sld5
Psf1
Mcm2

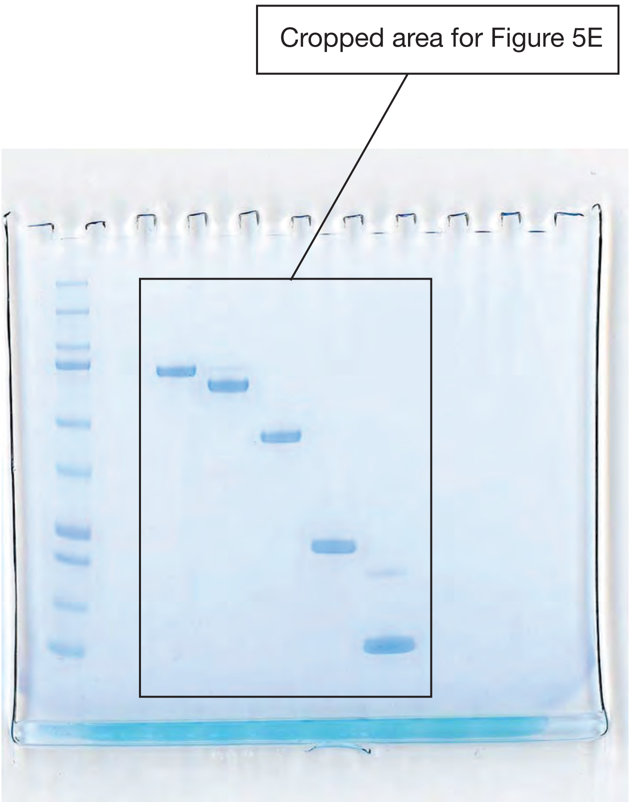
Cropped area for Figure 5E

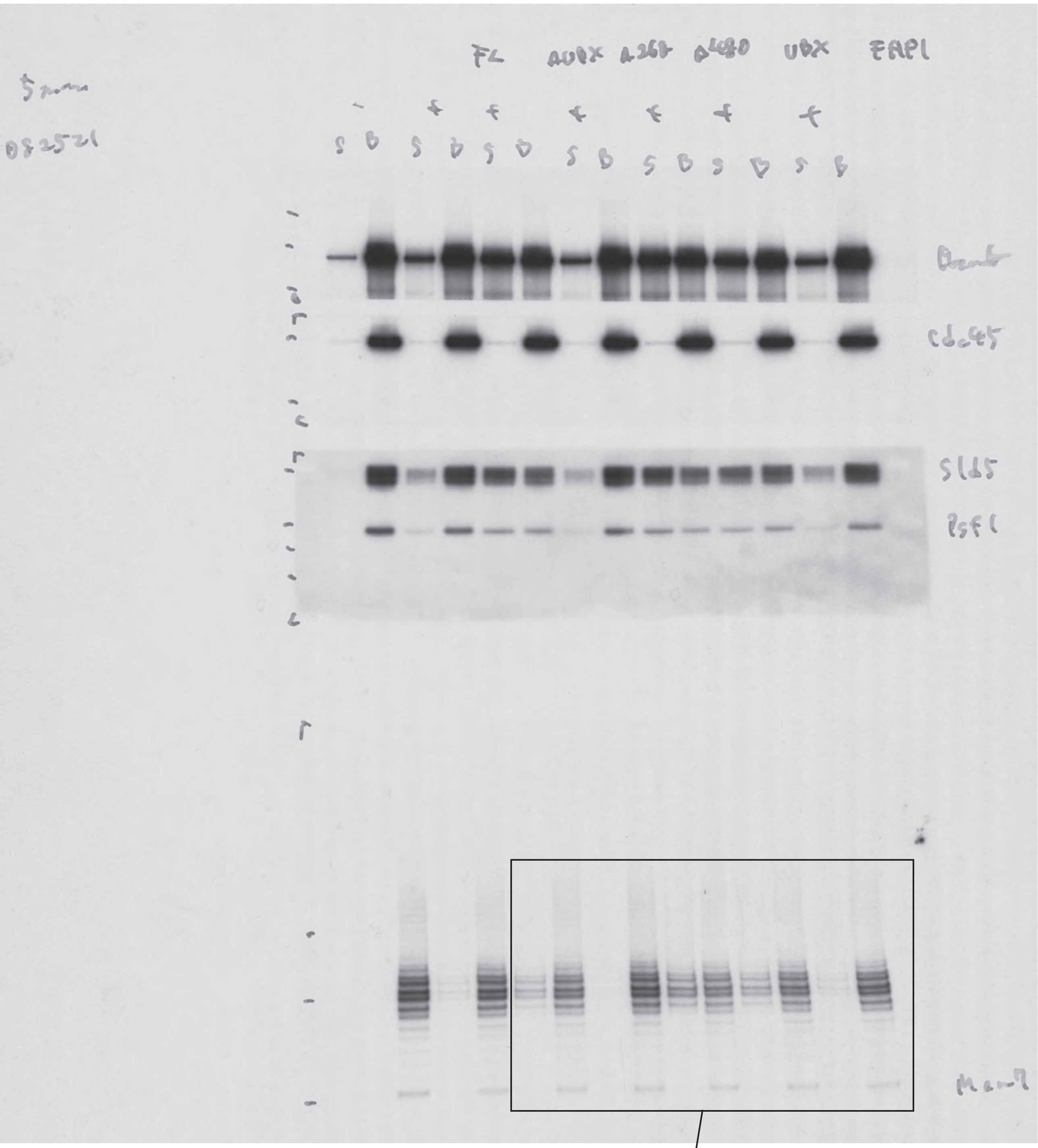

Cropped area for Figure 5F
Mcm7

Cropped area for Figure 5F
Mcm6

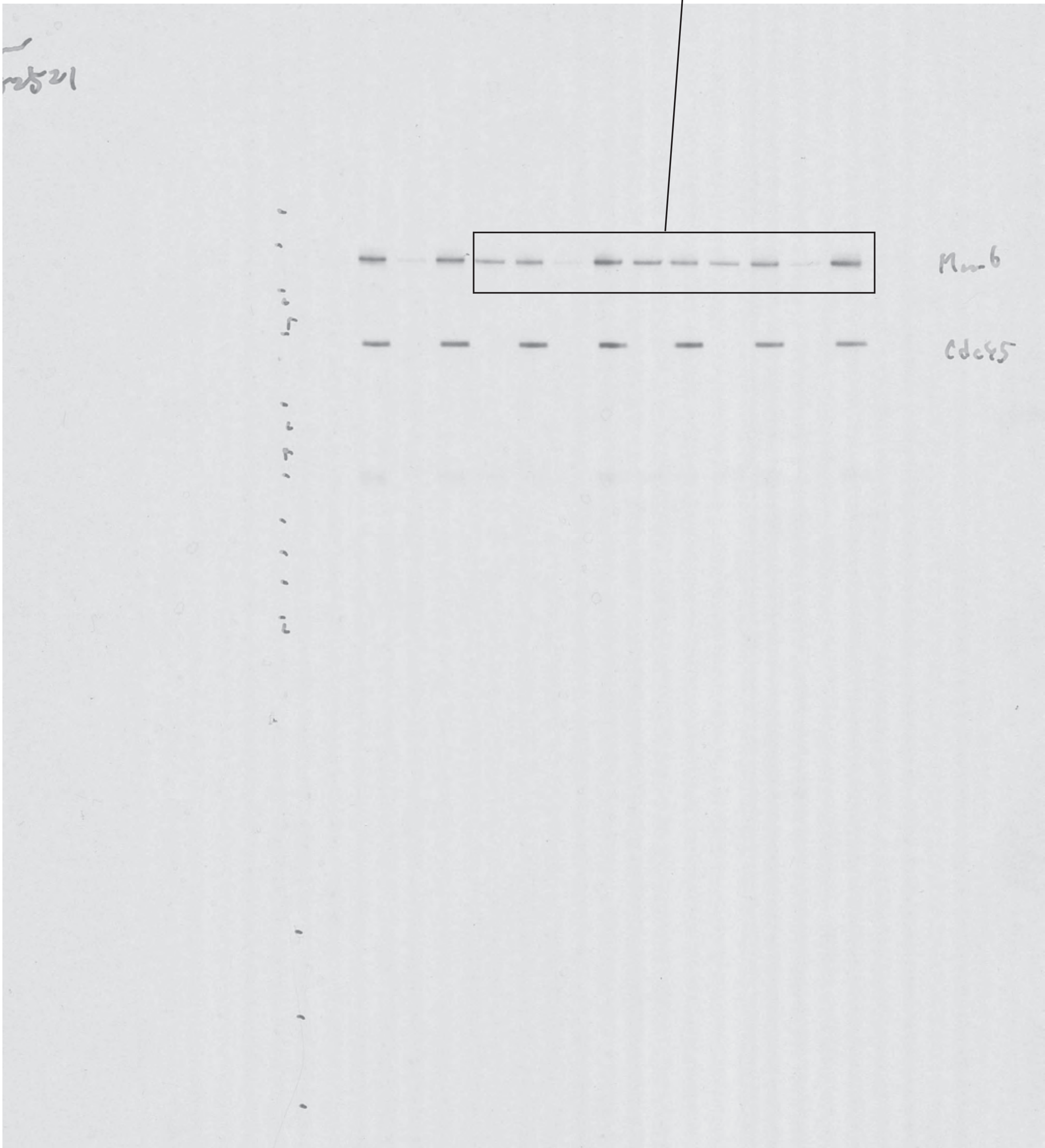

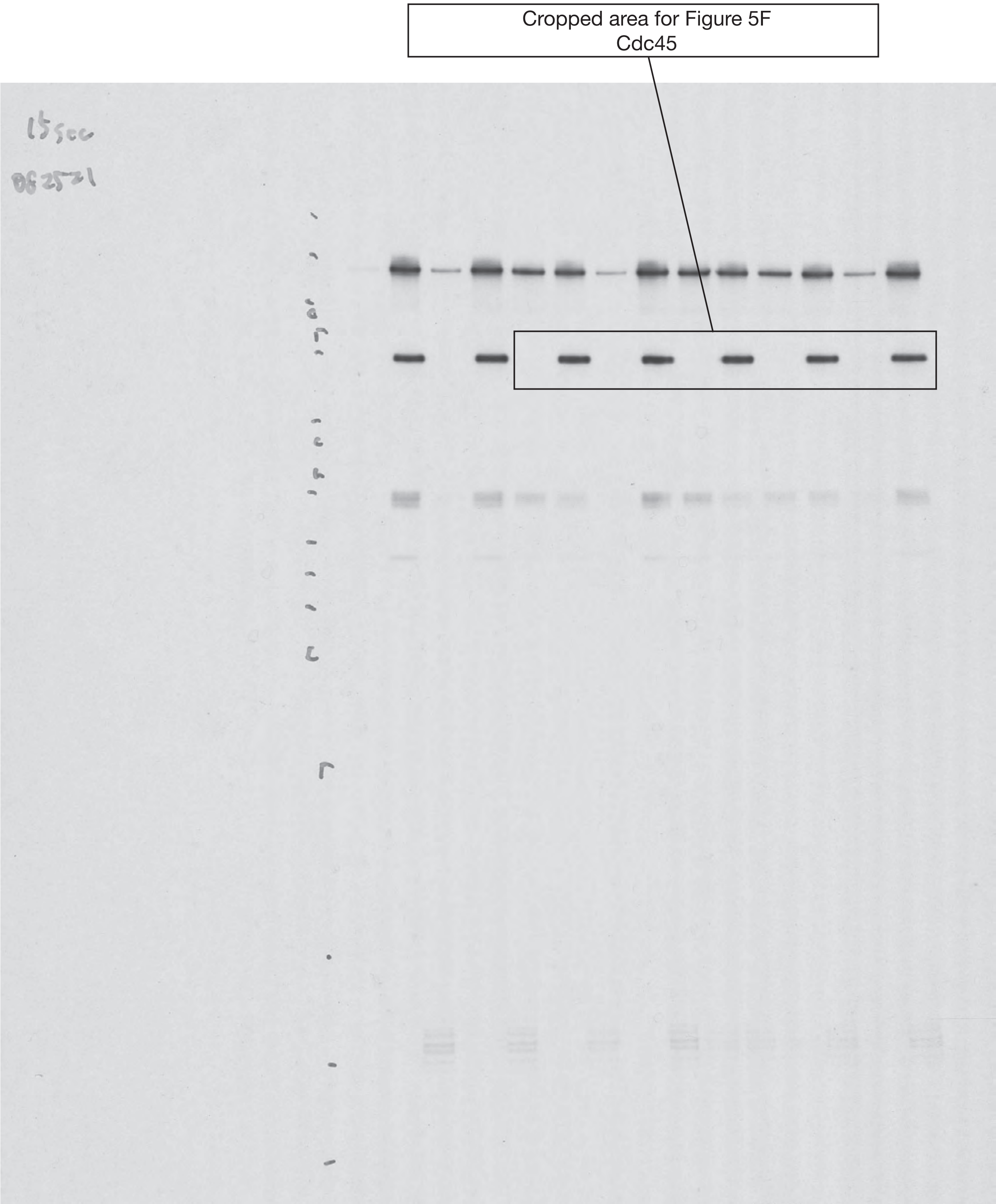
Cropped area for Figure 5F
Cdc45

Cropped area for Figure 5F
Sld5

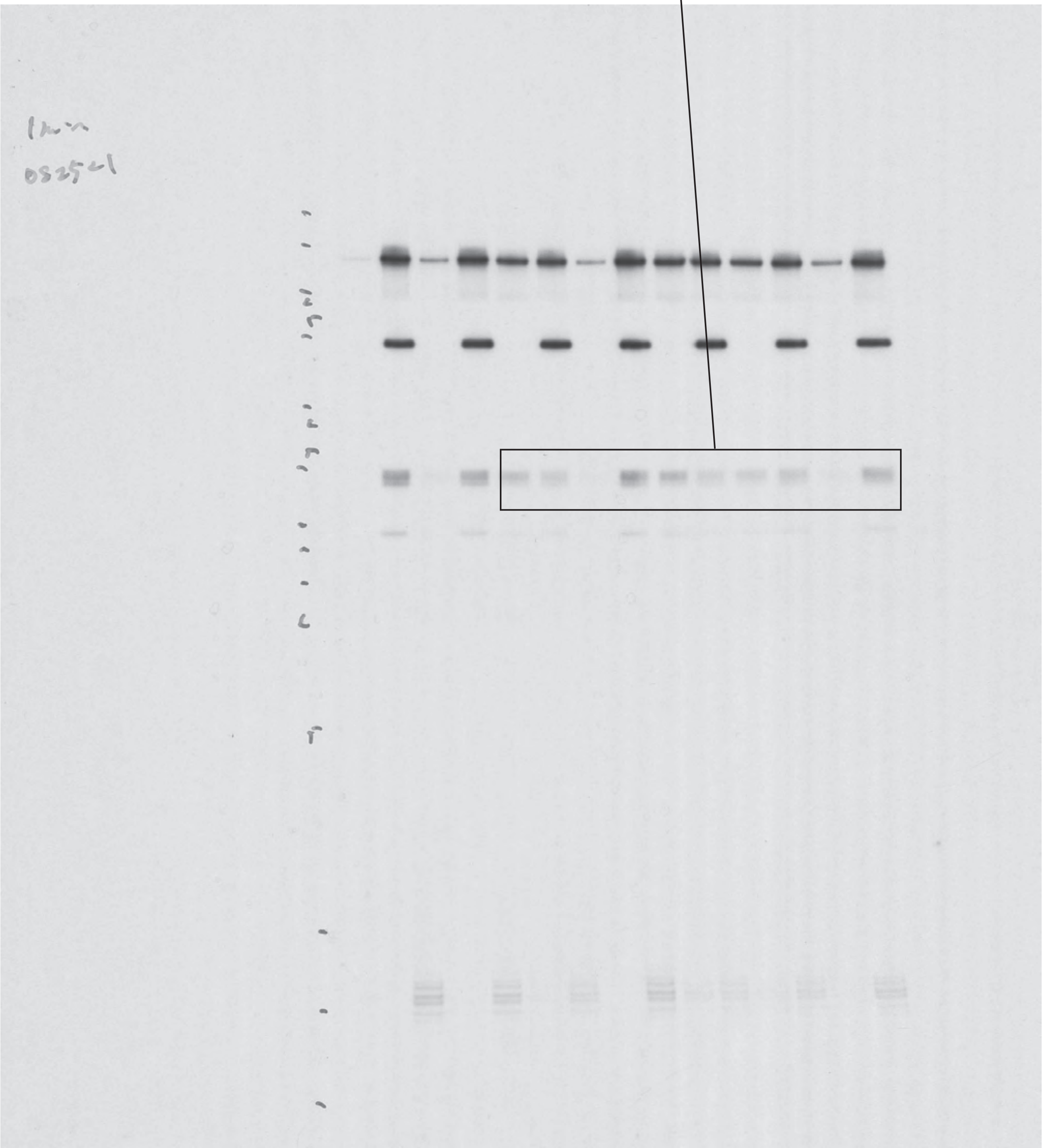

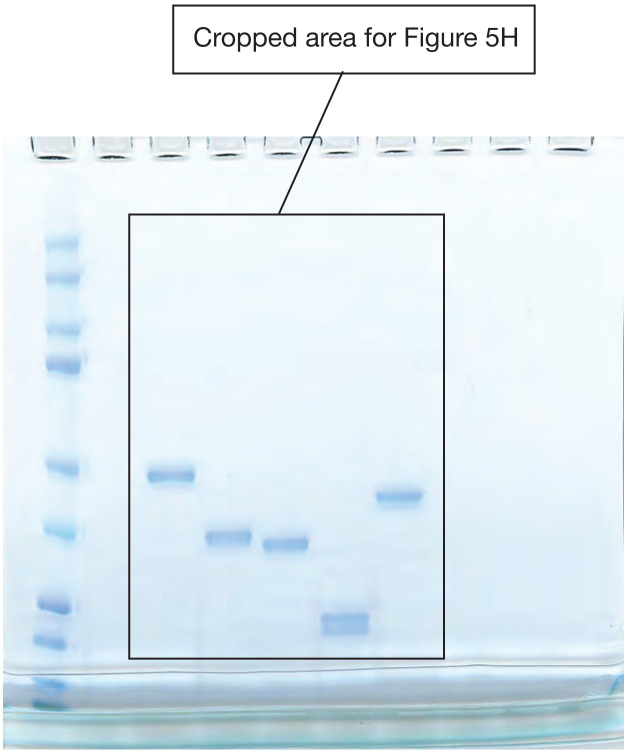
Cropped area for Figure 5H

Cropped area for Figure 5I
Cdc45

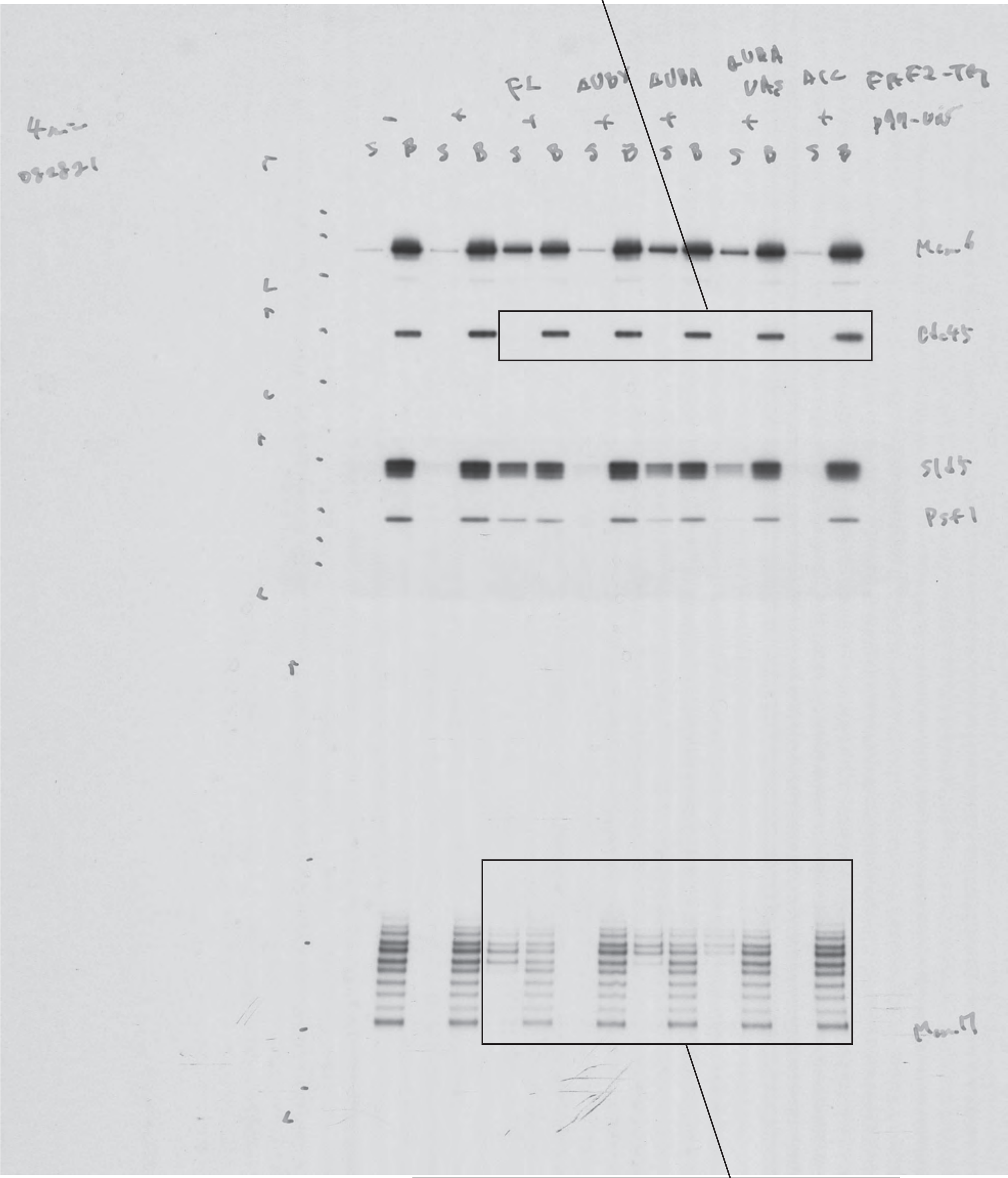

Cropped area for Figure 5I
Mcm7

Cropped area for Figure 5I
Mcm6

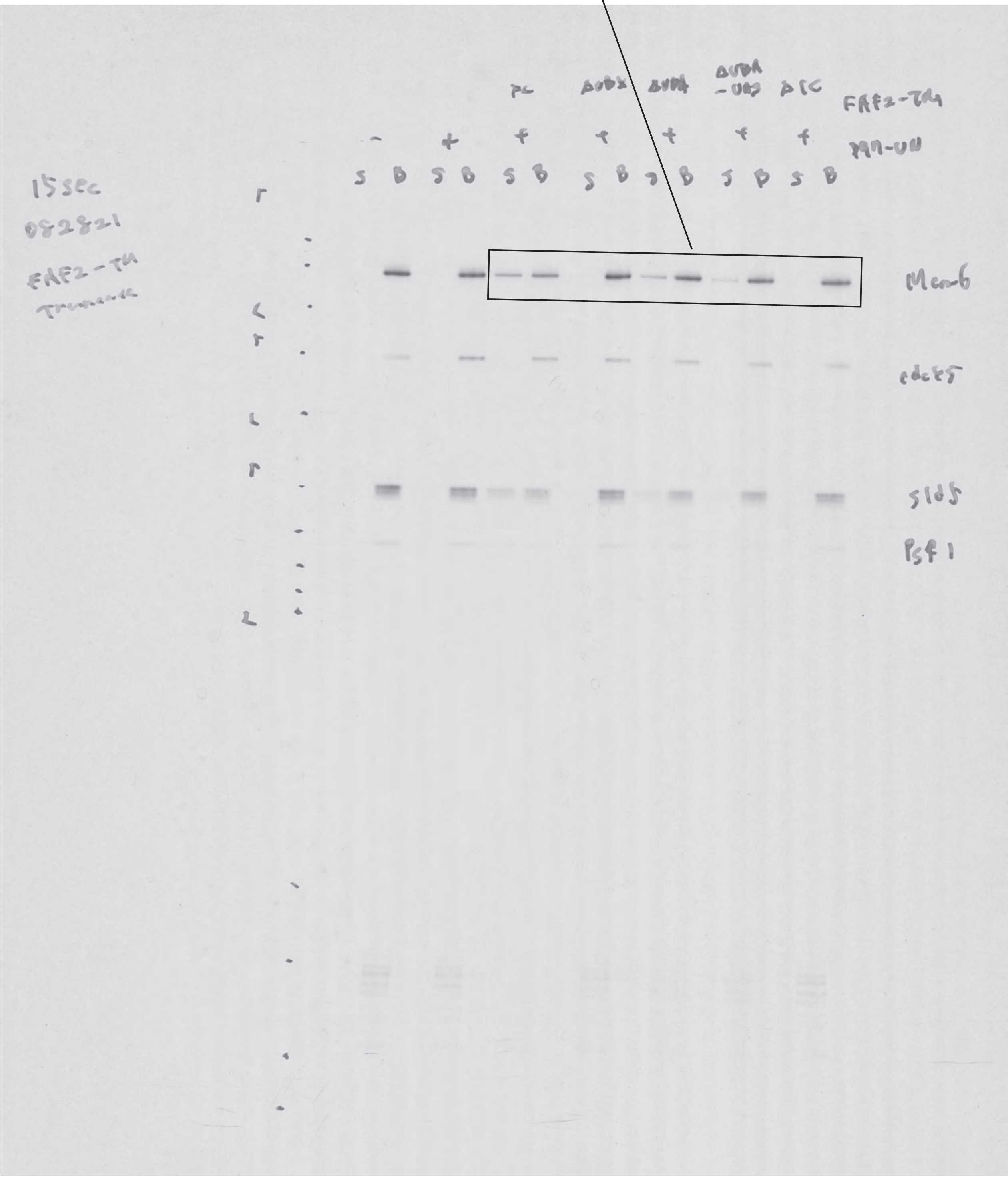

Cropped area for Figure 5I
Sld5

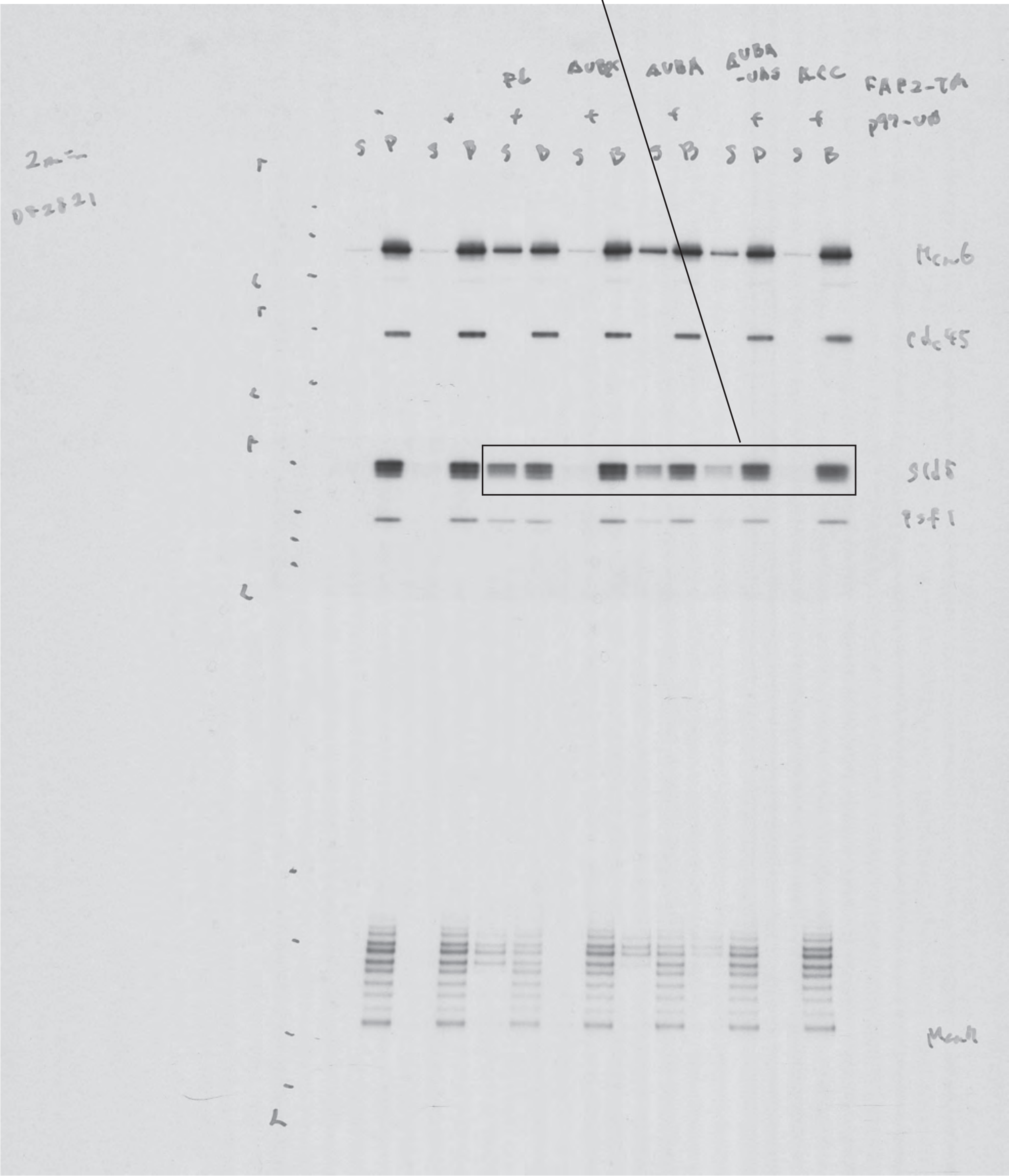

Supplement: Figure 5—source data 1. [file elife-76763-fig5-data1.pdf]
