# Supplementary material for: Multiple UBX proteins reduce the ubiquitin threshold of the mammalian p97-UFD1-NPL4 unfoldase
Source: eLife. 2022 Aug 3;11:e76763. doi: 10.7554/eLife.76763 (PMC9377798; doi:10.7554/eLife.76763)

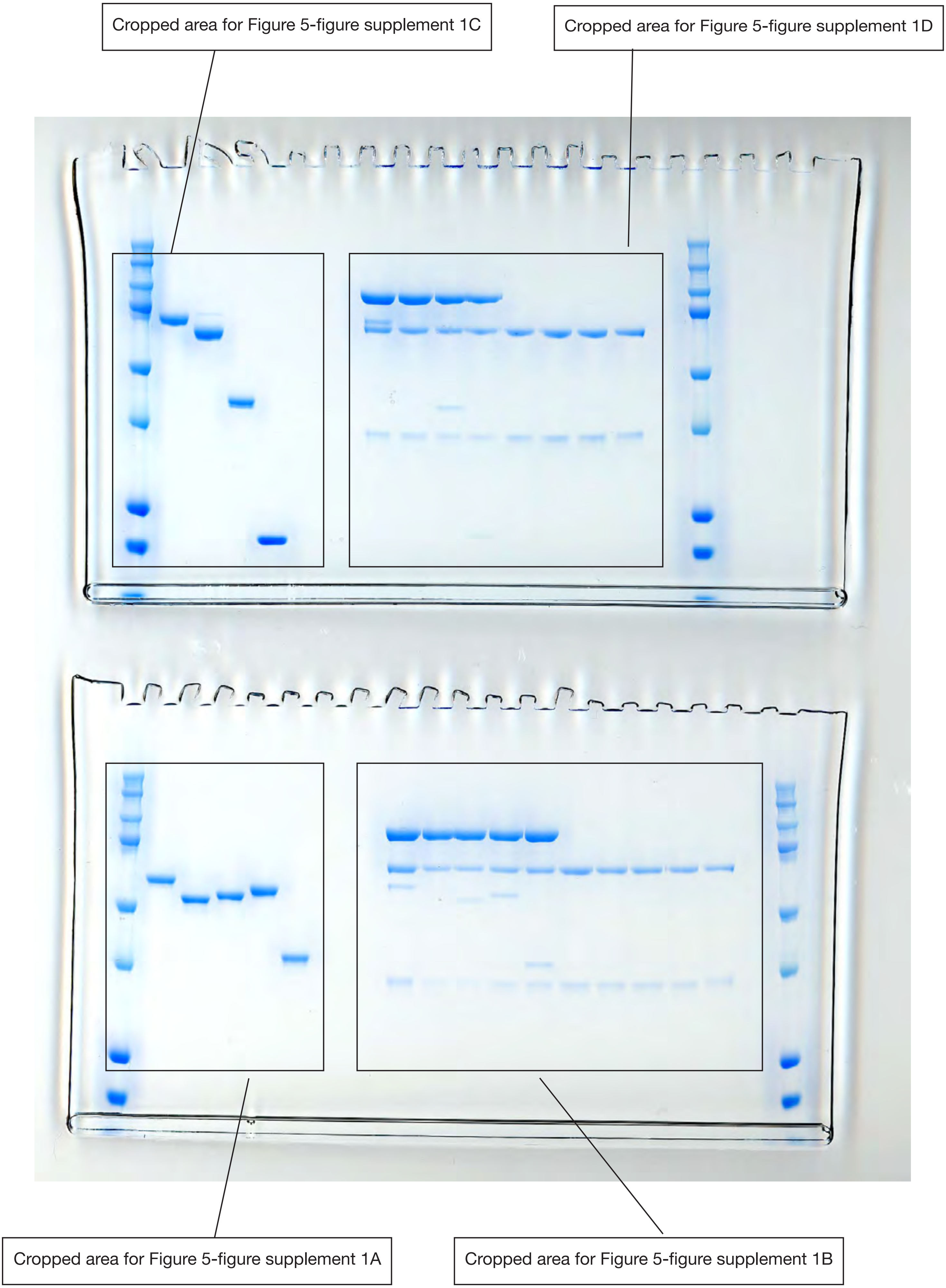
Cropped area for Figure 5-figure supplement 1C
Cropped area for Figure 5-figure supplement 1D
Cropped area for Figure 5-figure supplement 1A
Cropped area for Figure 5-figure supplement 1B

Cropped area for Figure 5-figure supplement 1E

Cropped area for Figure 5-figure supplement 1F

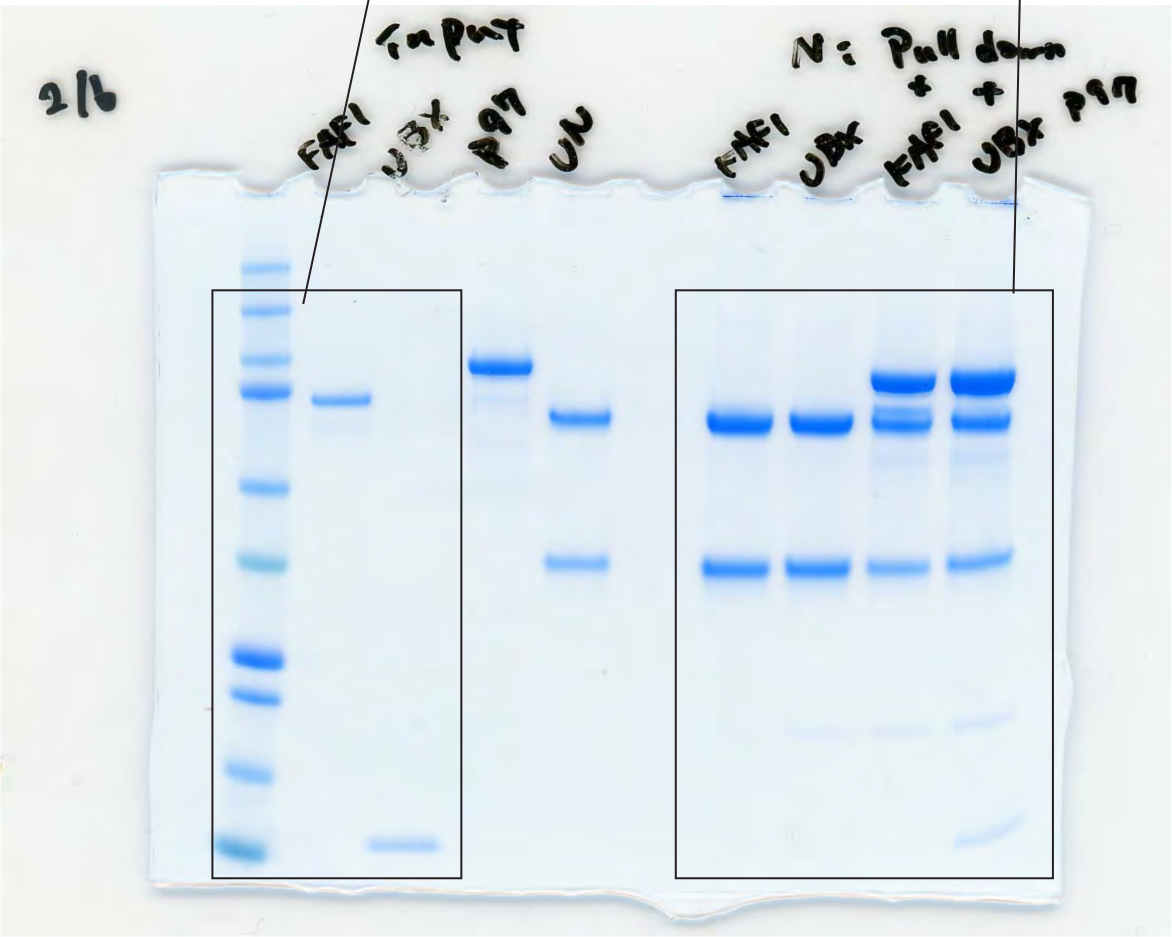

Supplement: Figure 5—figure supplement 1—source data 1. [file elife-76763-fig5-figsupp1-data1.pdf]
