# Supplementary material for: Multiple UBX proteins reduce the ubiquitin threshold of the mammalian p97-UFD1-NPL4 unfoldase
Source: eLife. 2022 Aug 3;11:e76763. doi: 10.7554/eLife.76763 (PMC9377798; doi:10.7554/eLife.76763)

Raw data file for Figure 5-figure supplement 3B
- sample scanned at 800 nm
with an Odyssey® CLx imaging System
(Li-Cor)

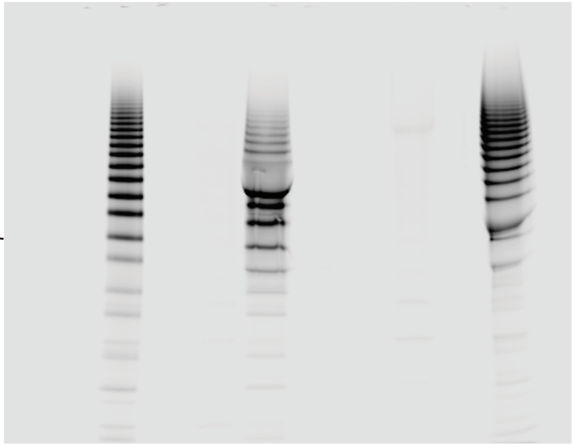

Raw data file for Figure 5-figure supplement 3B
- sample scanned at 700 nm
with an Odyssey® CLx imaging System
(Li-Cor)

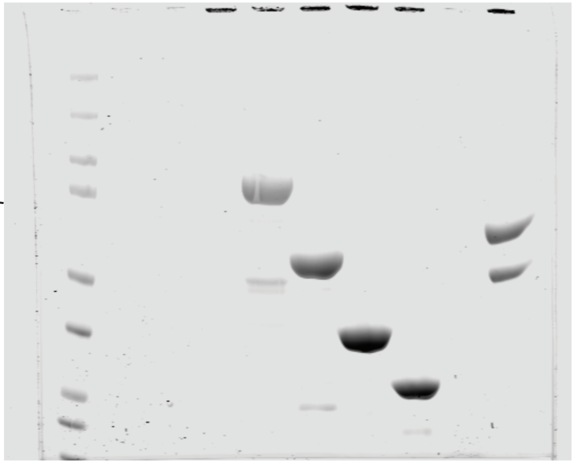

Supplement: Figure 5—figure supplement 3—source data 1. [file elife-76763-fig5-figsupp3-data1.pdf]
