# Supplementary material for: Multiple UBX proteins reduce the ubiquitin threshold of the mammalian p97-UFD1-NPL4 unfoldase
Source: eLife. 2022 Aug 3;11:e76763. doi: 10.7554/eLife.76763 (PMC9377798; doi:10.7554/eLife.76763)
Supplement: Figure 6—source data 1. [file elife-76763-fig6-data1.pdf]

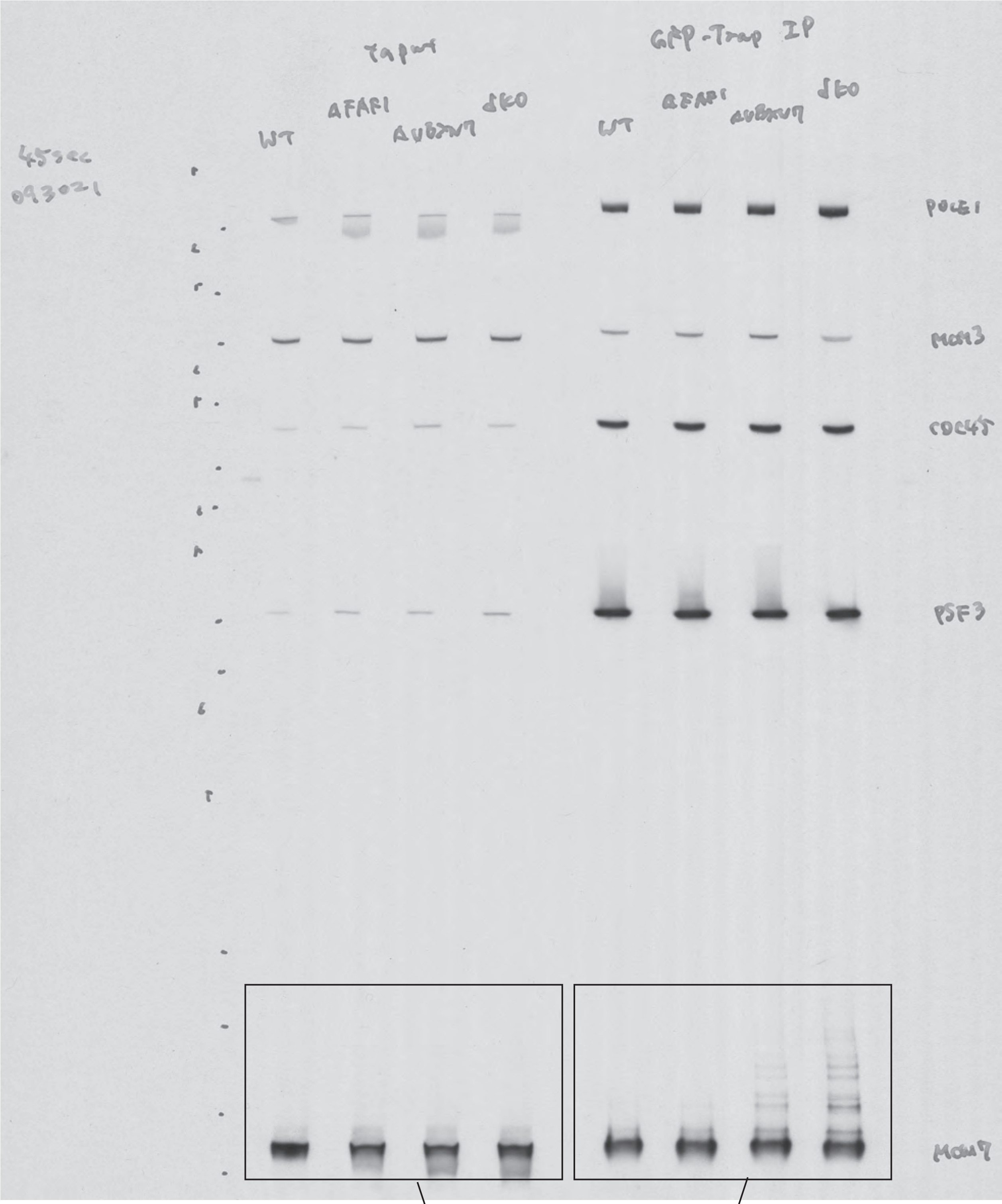

Cropped areas for Figure 6A
MCM7

Cropped areas for Figure 6A
MCM3

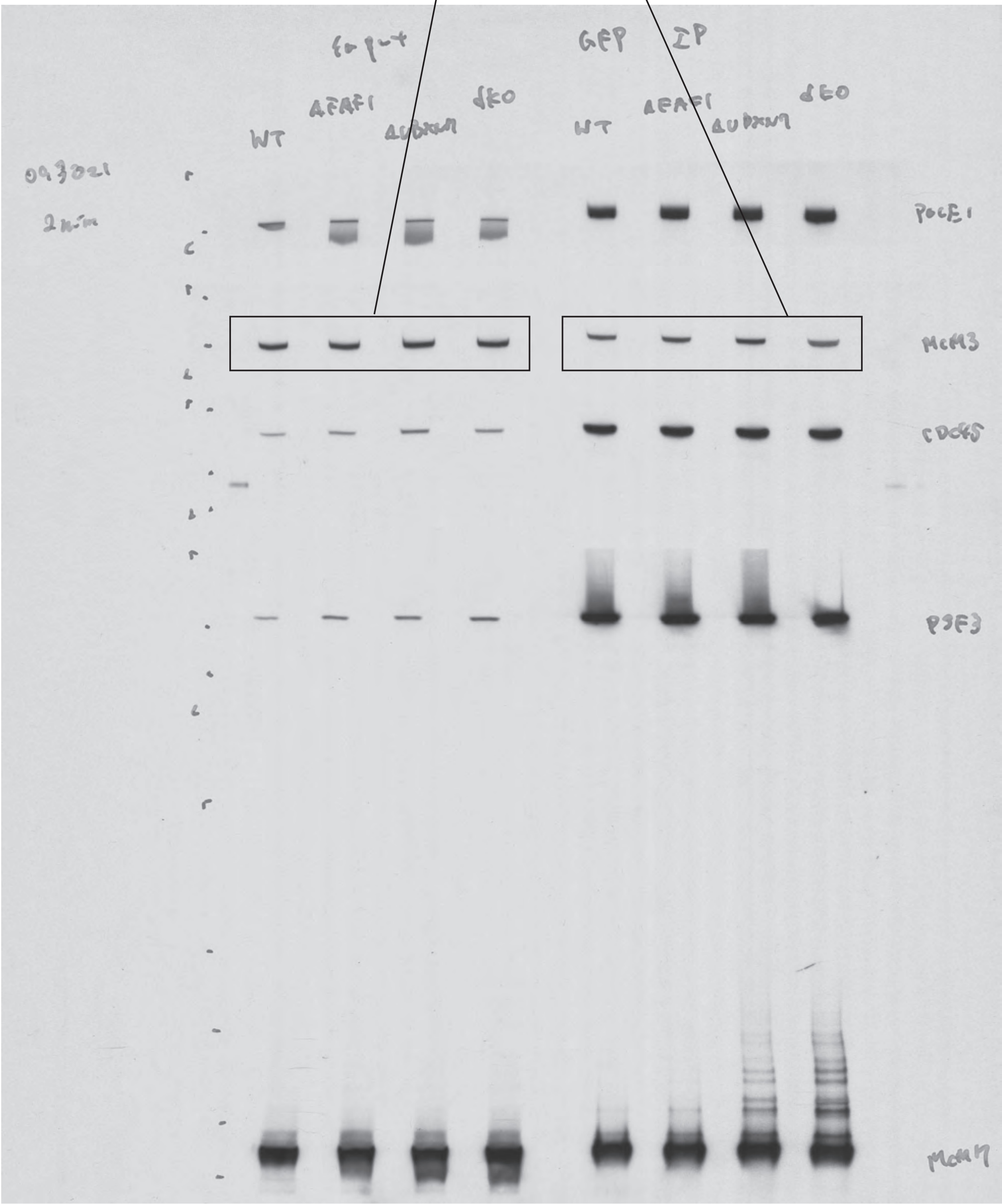

Cropped areas for Figure 6A
CDC45

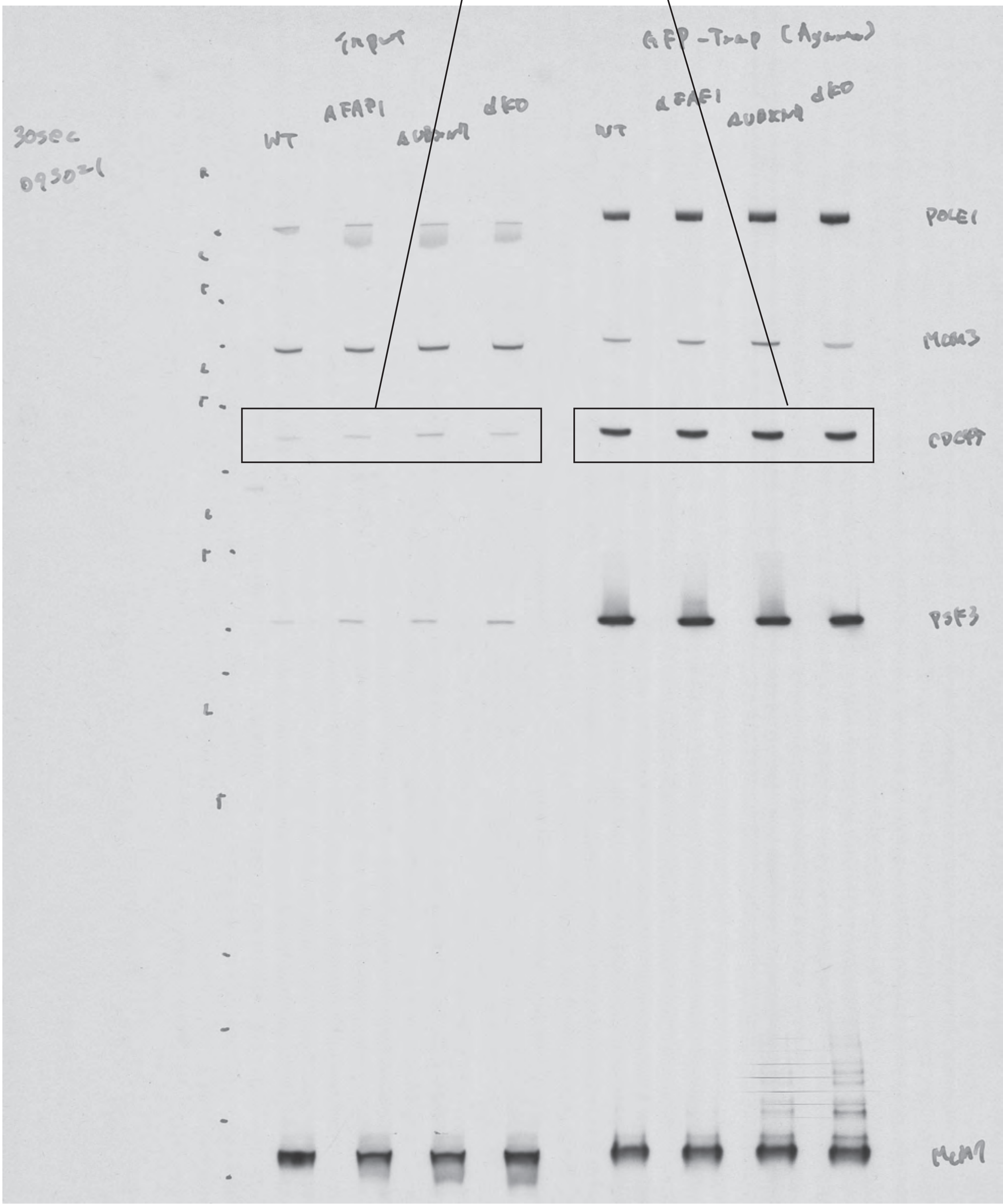

Cropped areas for Figure 6A
PSF3

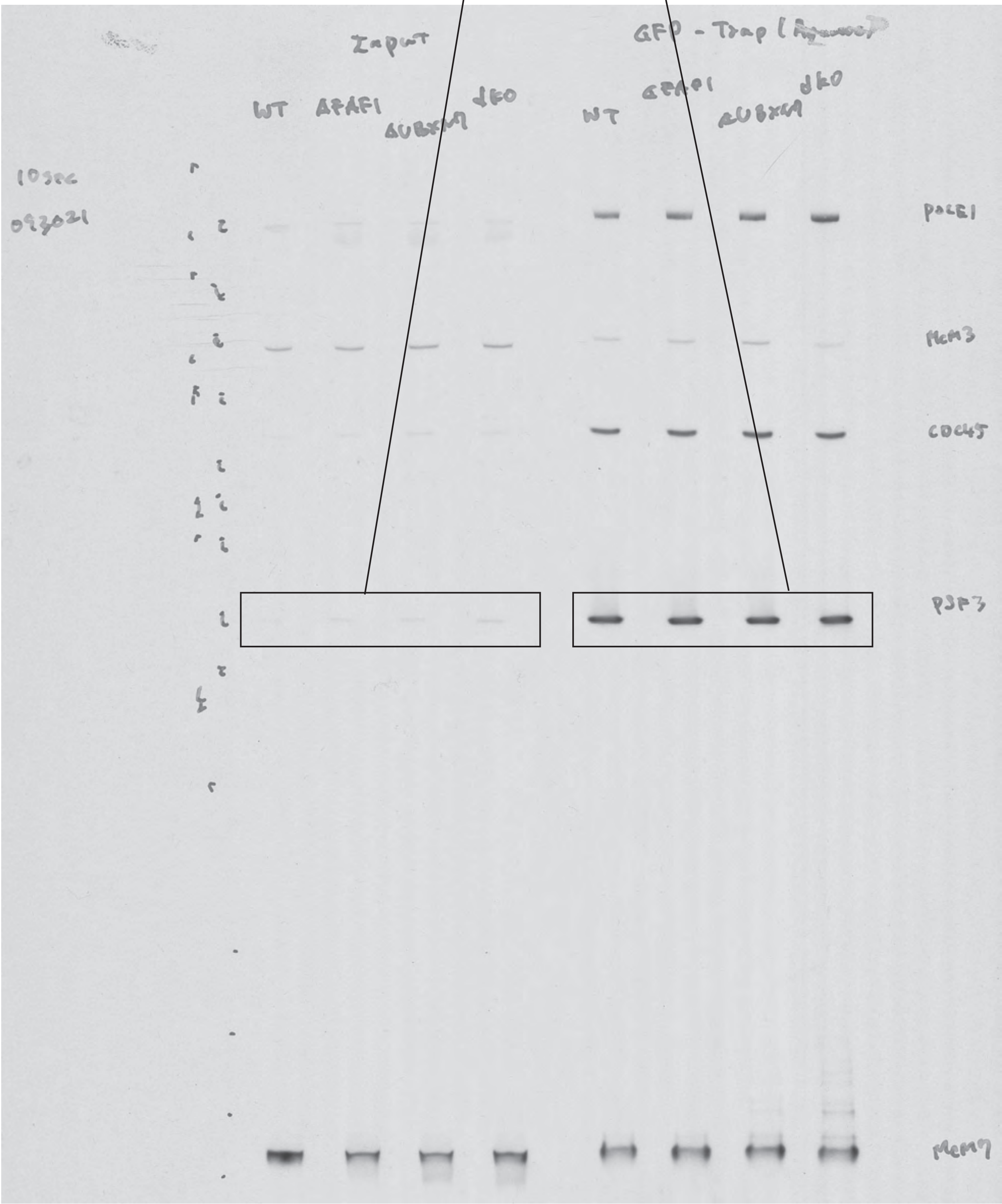

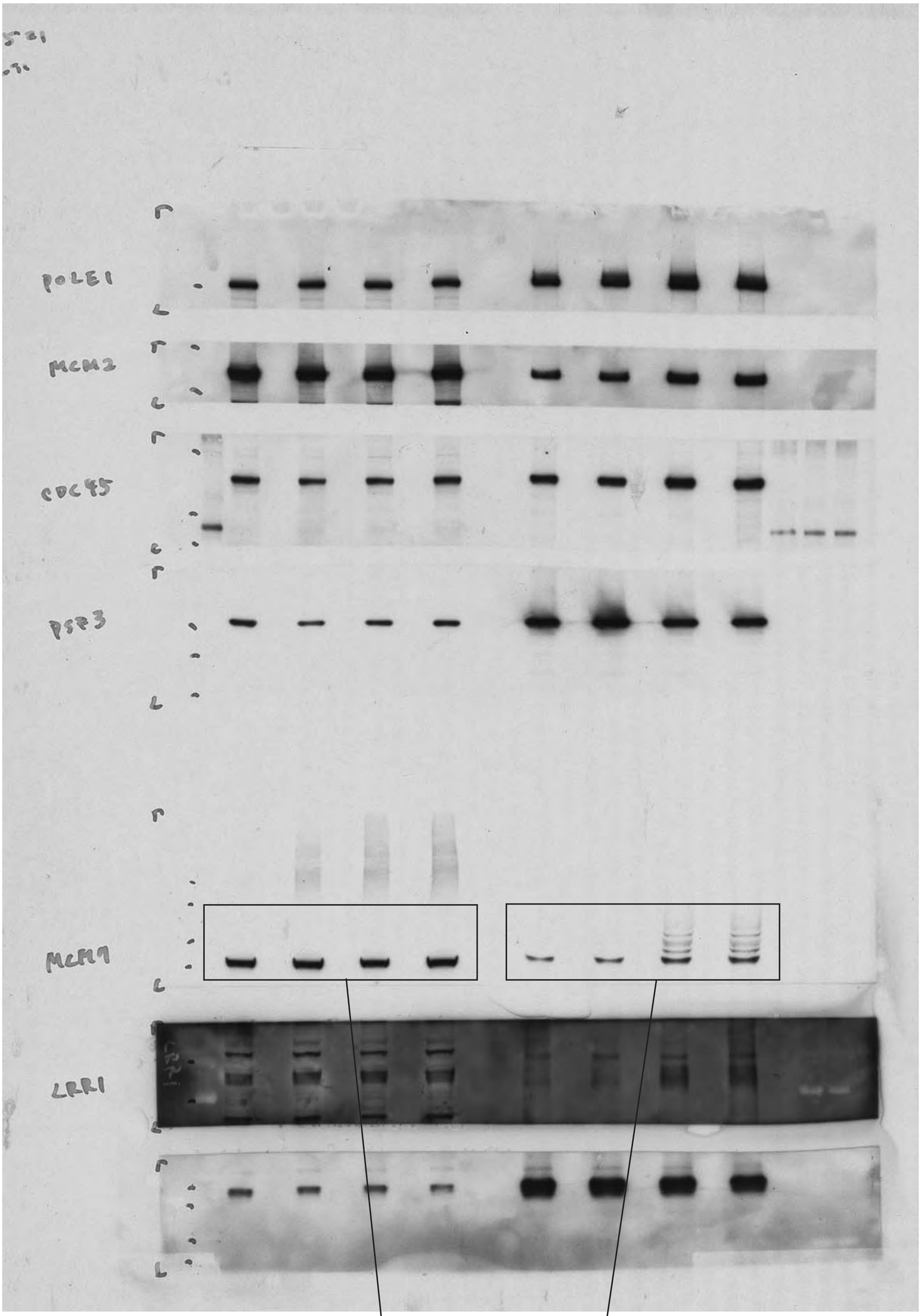

Cropped areas for Figure 6B
MCM7 (short exposure)

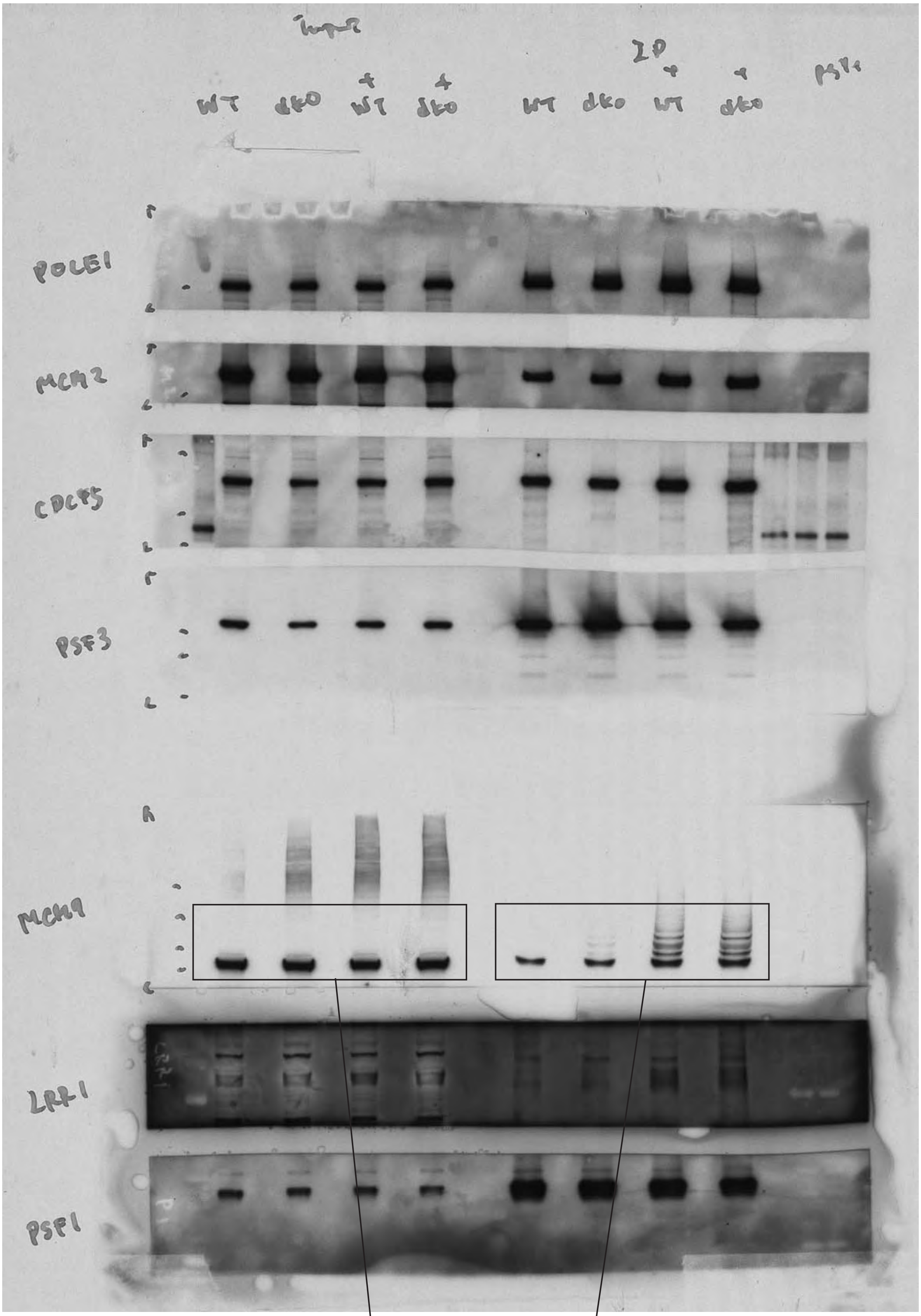

Cropped areas for Figure 6B
MCM7 (long exposure)

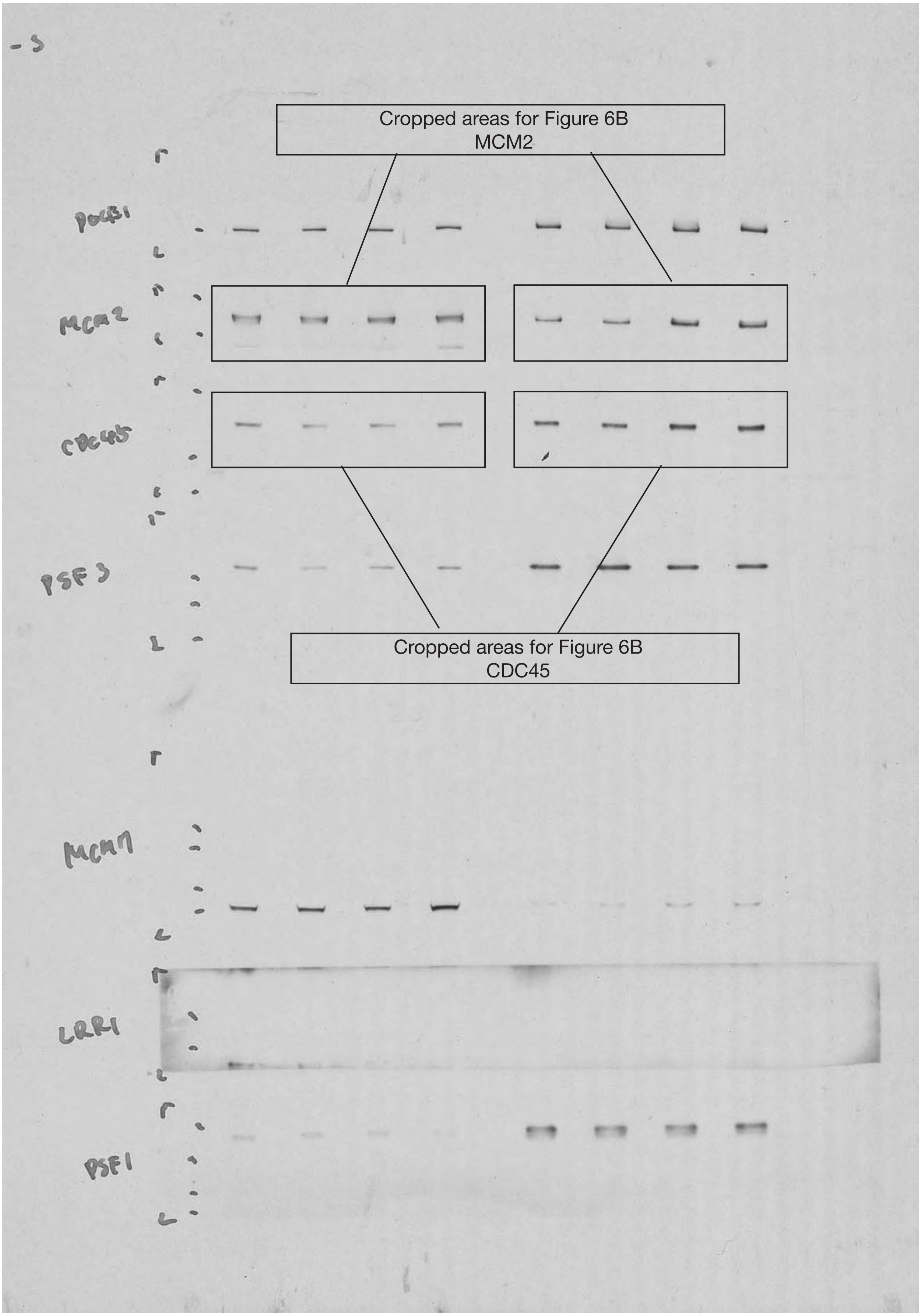
Cropped areas for Figure 6B
MCM2
MCM2
CDC45
PSF3
Cropped areas for Figure 6B
CDC45
MCM7
LRR1
PSF1

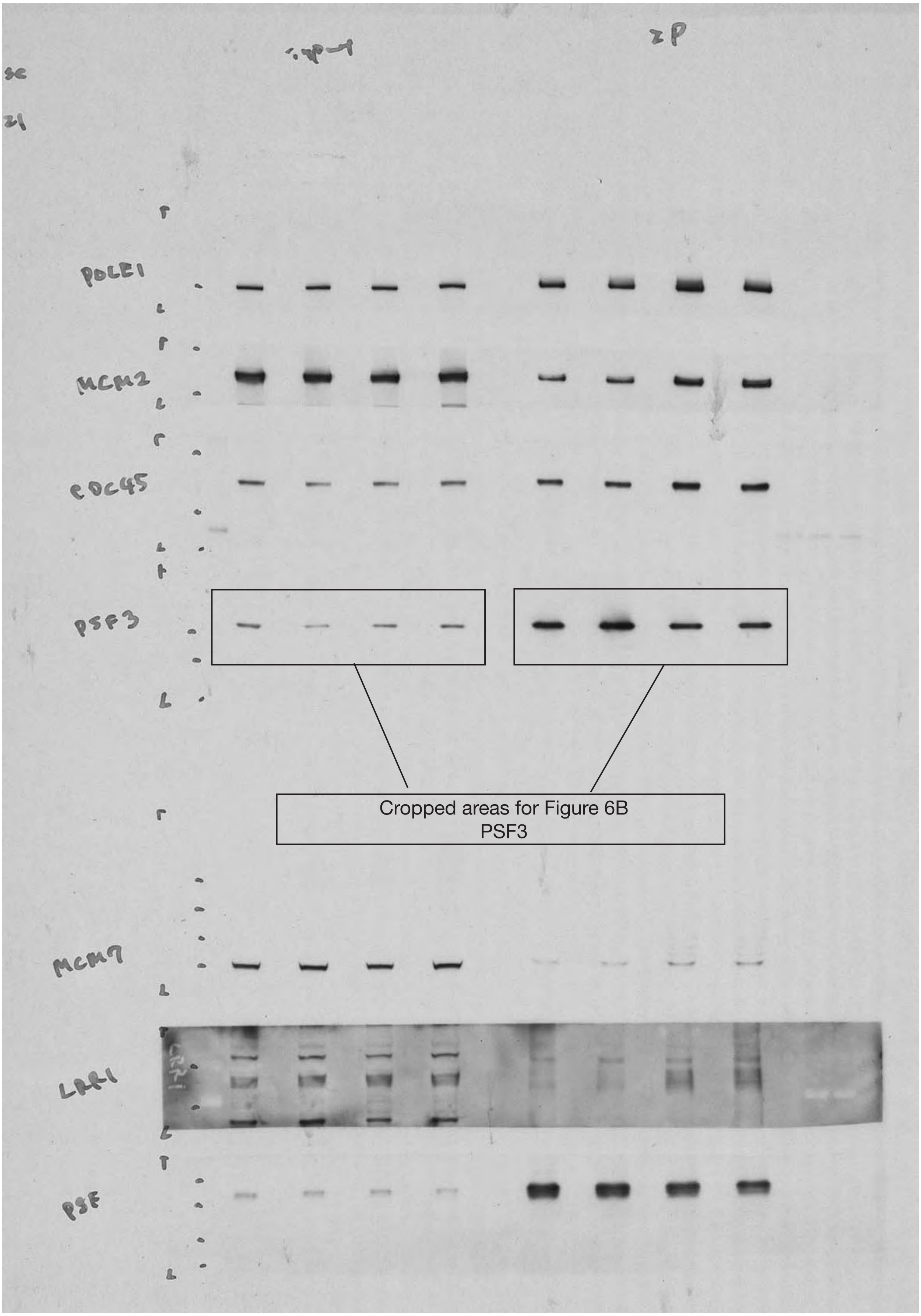

POLE1
MCM2
CDC45
PSF3
Cropped areas for Figure 6B
PSF3
MCM7
