# Supplementary material for: Multiple UBX proteins reduce the ubiquitin threshold of the mammalian p97-UFD1-NPL4 unfoldase
Source: eLife. 2022 Aug 3;11:e76763. doi: 10.7554/eLife.76763 (PMC9377798; doi:10.7554/eLife.76763)
Supplement: Figure 6—figure supplement 3—source data 1. [file elife-76763-fig6-figsupp3-data1.pdf]

Cropped area for Figure 6-figure supplement 3D
FAF1

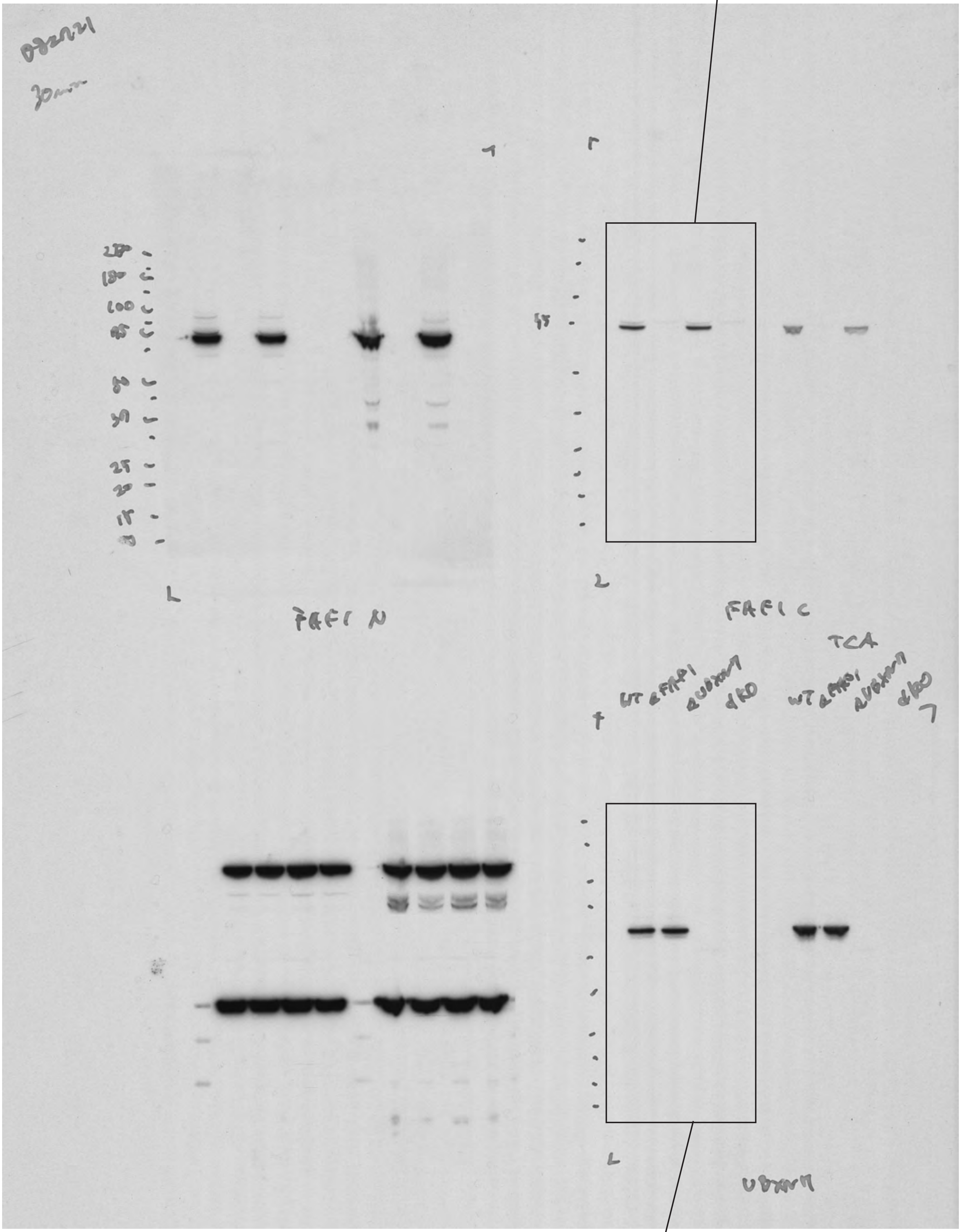

Cropped area for Figure 6-figure supplement 3D
UBXN7

Cropped area for Figure 6-figure supplement 3D
Ponceau S

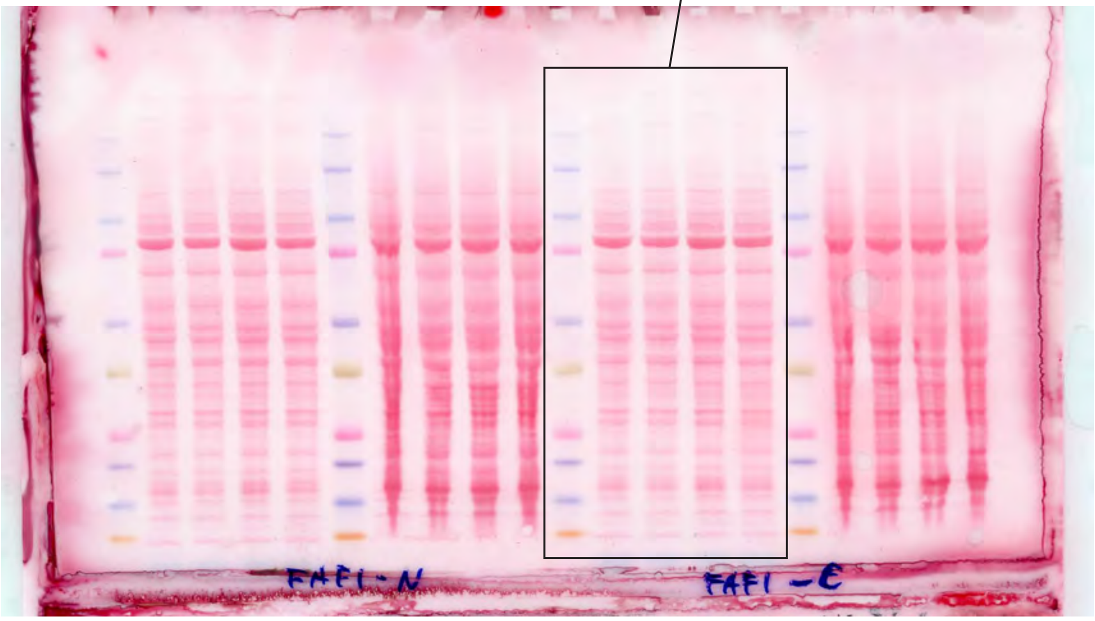

Cropped area for Figure 6-figure supplement 3E
FAF1

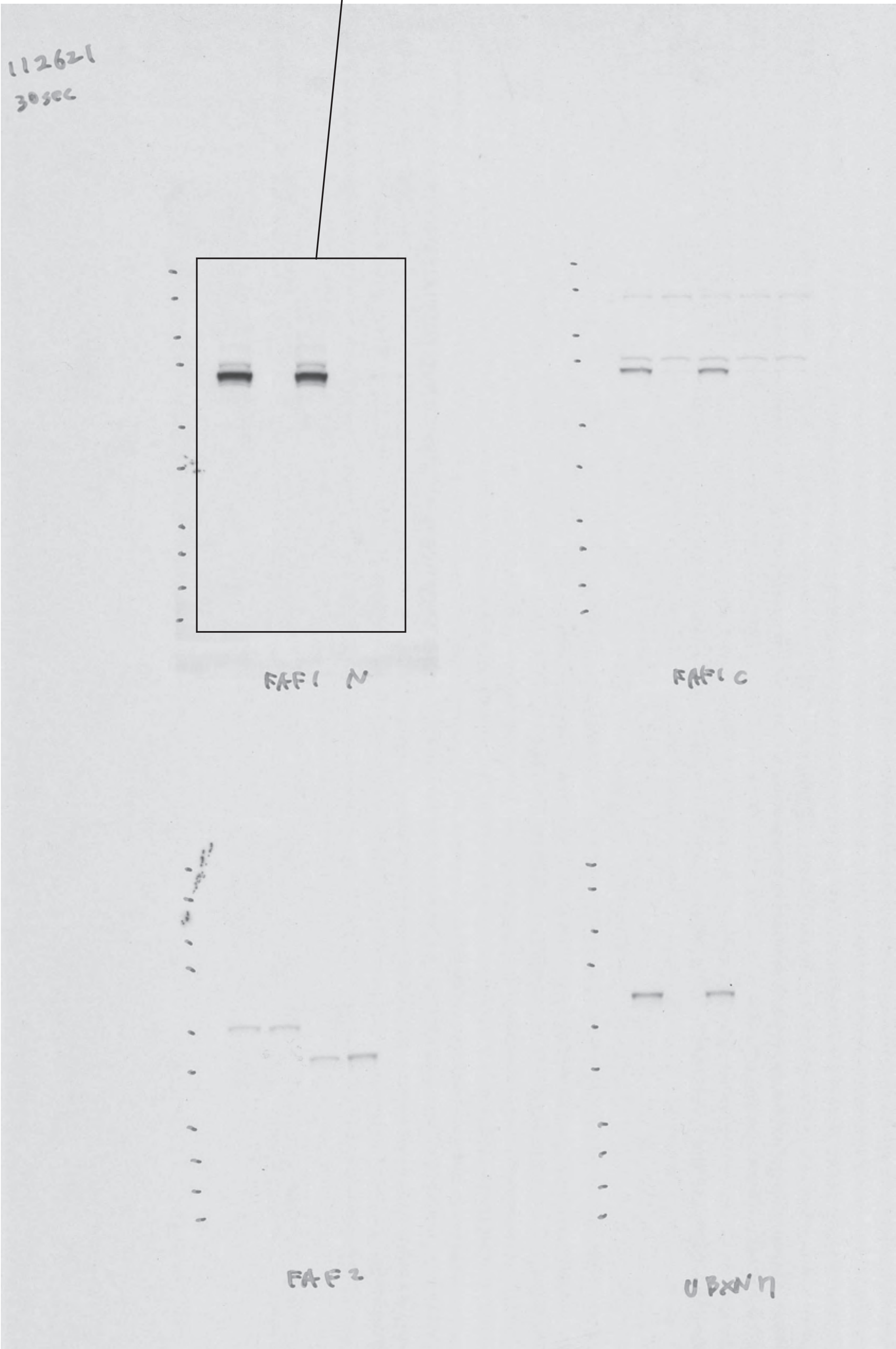

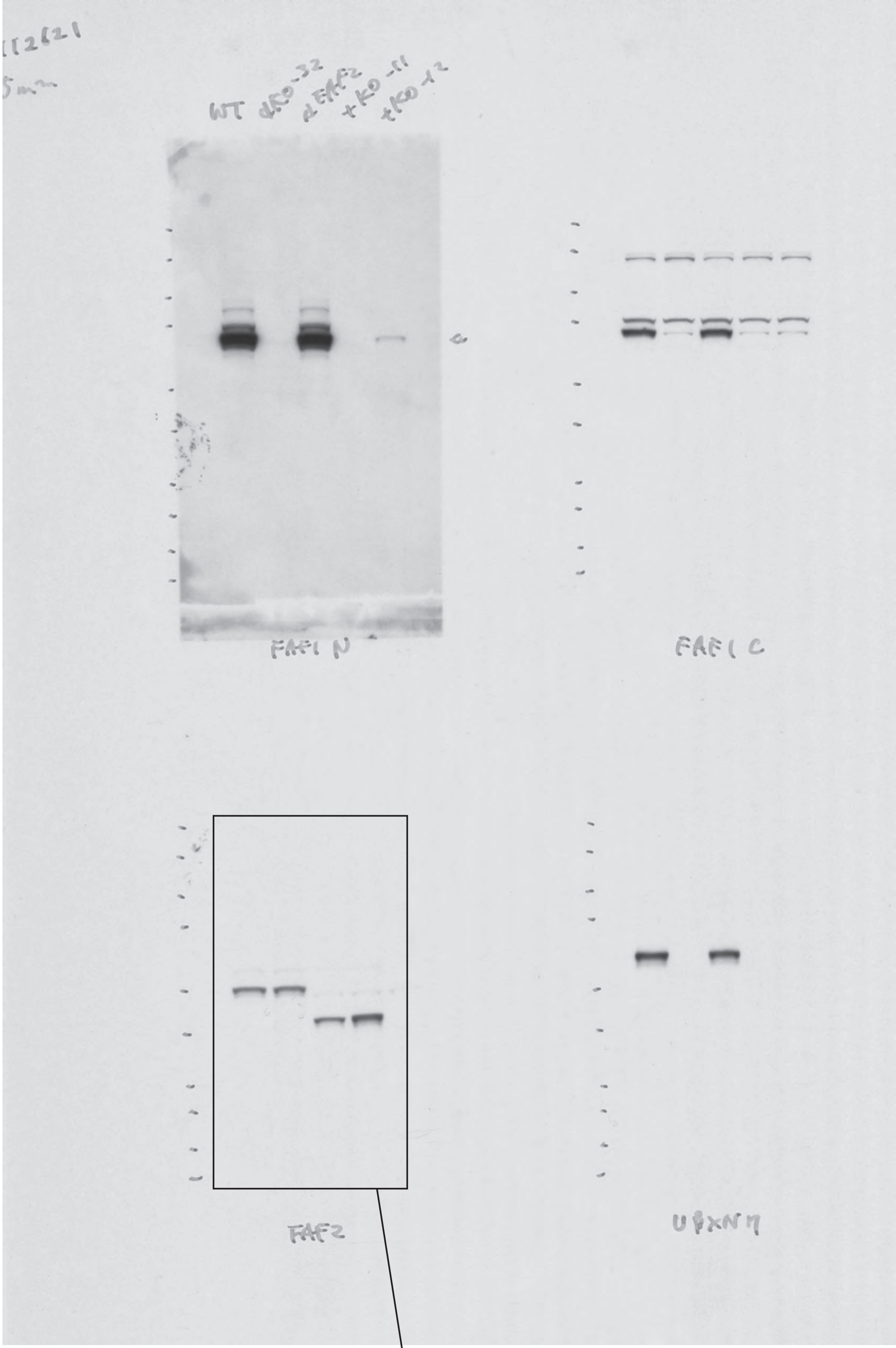

Cropped area for Figure 6-figure supplement 3E
FAF2

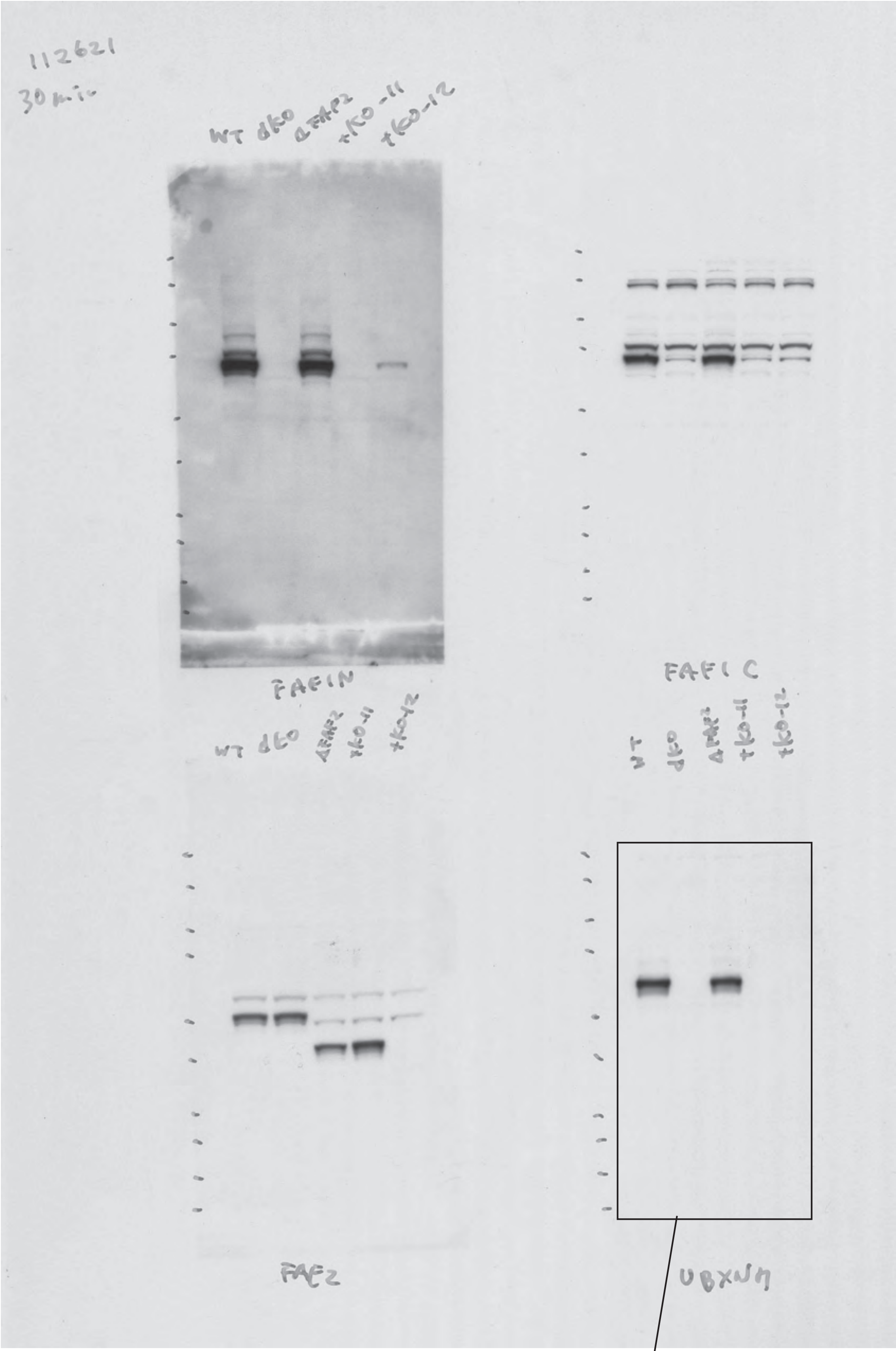

Cropped area for Figure 6-figure supplement 3E
UBXN7

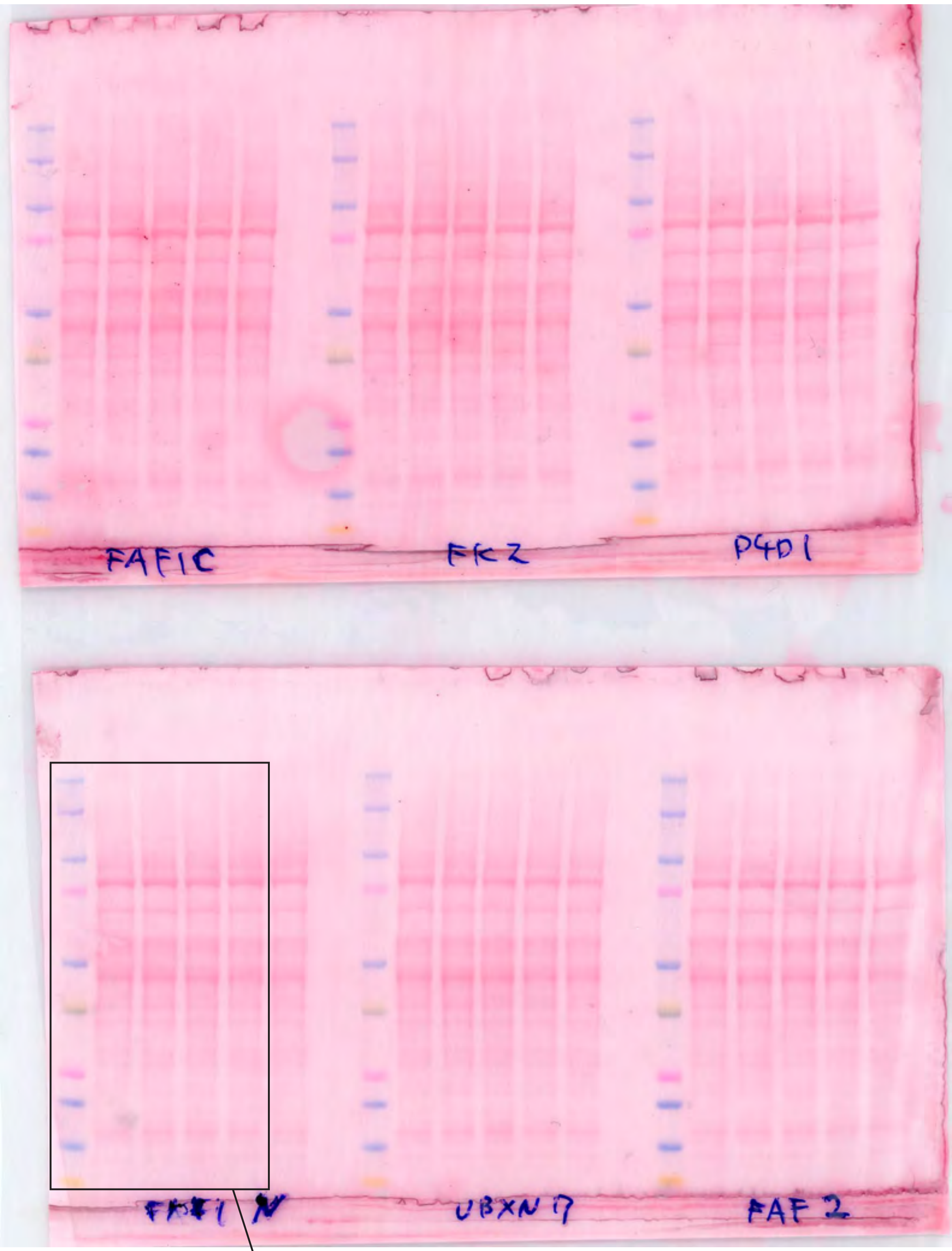

Cropped area for Figure 6-figure supplement 3E
Ponceau S
