# Supplementary material for: Multiple UBX proteins reduce the ubiquitin threshold of the mammalian p97-UFD1-NPL4 unfoldase
Source: eLife. 2022 Aug 3;11:e76763. doi: 10.7554/eLife.76763 (PMC9377798; doi:10.7554/eLife.76763)
Supplement: Supplementary file 1. [file elife-76763-supp1.docx]

**Supplementary File 1**

Reagents and resources used in this study.

| **REAGENT or RESOURCE** | SOURCE | IDENTIFIER |
| --- | --- | --- |
| **Antibodies** |  |  |
| Mouse MCM2 (sheep polyclonal)  Antigen: 1-222 of Mouse MCM2 | MRC PPU Reagents and Services | S883D |
| Mouse MCM3 (sheep polyclonal)  Antigen: 1-222 of Mouse MCM3 | MRC PPU Reagents and Services | SA387 |
| Human MCM7 (mouse monoclonal)  Antigen: Full-length Human MCM7 | Santa Cruz Biotechnology | sc-9966 |
| Mouse PSF3 (sheep polyclonal)  Antigen: Full-length Mouse PSF3 | MRC PPU Reagents and Services | S882D |
| Mouse CDC45 (sheep polyclonal)  Antigen: Mouse CDC45 1-222 | MRC PPU Reagents and Services | S907D |
| Budding yeast Mcm6 (sheep polyclonal)  Antigen: 801-1017 of *S. cerevisiae* Mcm6 | MRC PPU Reagents and Services | DA003 |
| Budding yeast Mcm7 (sheep polyclonal)  Antigen: 1-222 of *S. cerevisiae* Mcm7 | Labib laboratory | [19] |
| Budding yeast Cdc45 (sheep polyclonal)  Antigen: 1-222 of *S. cerevisiae* Cdc45 | Labib laboratory | [29] |
| Budding yeast Sld5 (sheep polyclonal)  Antigen: Full length *S. cerevisiae* Sld5 | Labib laboratory | [32] |
| Budding yeast Psf1 (sheep polyclonal)  Antigen: Full length *S. cerevisiae* Psf1 | Labib laboratory | [58] |
| Worm MCM-2 (sheep polyclonal)  Antigen: 1-222 aa of worm MCM-2 | MRC PPU Reagents and Services | S750D |
| Worm MCM-7 (sheep polyclonal)  Antigen: 1-222 aa of worm MCM-7 | MRC PPU Reagents and Services | S797D |
| Worm SLD-5 (sheep polyclonal)  Antigen: Full-length worm SLD-5 | MRC PPU Reagents and Services | SA419 |
| Mouse FAF1 N-terminus (sheep polyclonal)  Antigen: 1-222 of Mouse FAF1 | MRC PPU Reagents and Services | SA278 |
| Mouse FAF1 C-terminus (sheep polyclonal)  Antigen: 417-595 of Mouse FAF1 | MRC PPU Reagents and Services | SA491 |
| Human FAF2 (sheep polyclonal)  Antigen: Human FAF2 lacking 90-118 (transmembrane domain) | MRC PPU Reagents and Services | DA153 |
| Human UBXN7 (sheep polyclonal)  Antigen: Full-length Human UBXN7 | MRC PPU Reagents and Services | S409D |
| anti-FLAG M2 (mouse monoclonal) | Sigma-Aldrich | F3165 |
| anti-sheep IgG HRP | Sigma-Aldrich | A3415 |
| anti-mouse IgG HRP | Sigma-Aldrich | A4416 |
| **Chemicals, Peptides, and Recombinant Proteins** |  |  |
| Phusion® High-Fidelity DNA Polymerase | New England Biolabs | M0530 |
| Ex Taq DNA polymerase | TaKaRa | RR001 |
| TOPO TA cloning Kit | Invitrogen | K457540 |
| Gibson Assembly Cloning Kit | New England Biolabs | E2611 |
| XpressRef Universal Total human RNA | QIAGEN | 338112 |
| PrimeScript™ RT-PCR Kit | TaKaRa | RR014 |
| T4 polynucleotide kinase | New England Biolabs | M201 |
| T4 DNA ligase | New England Biolabs | M202 |
| Ubiquitin PrG | MRC PPU Reagents and Services | DU49003 |
| Pierce Universal Nuclease | ThermoFisher Scientific | 88702 |
| Gelatin | Sigma-Aldrich | G1890 |
| DMEM | ThermoFisher Scientific | 11960044 |
| no phenol red DMEM | ThermoFisher Scientific | 21063029 |
| OPTI-MEM | ThermoFisher Scientific | 31985062 |
| Knockout serum replacement | ThermoFisher Scientific | 10828028 |
| L-Glutamine | ThermoFisher Scientific | 25030081 |
| Penicillin-Streptomycin | ThermoFisher Scientific | 15140122 |
| Sodium Pyruvate | ThermoFisher Scientific | 11360070 |
| non-essential amino acids | ThermoFisher Scientific | 11140050 |
| β-mercaptoethanol | Sigma-Aldrich | M6520 |
| LIF | MRC PPU Reagents and Services | DU1715 |
| Trypsin-EDTA | ThermoFisher Scientific | 25300054 |
| Lipofectamine™ RNAiMAX Transfection Reagent | ThermoFisher Scientific | 13778075 |
| MLN-4924 (inhibitor of E1 enzyme for protein neddylation) | Activebiochem | A1139 |
| Hoechst 33342 | Sigma-Aldrich | B2261 |
| IRDye-800CW Maleimide | Li-Cor | 929-80020 |
| Anti-FLAG M2 affinity gel | Sigma-Aldrich | A2220 |
| Ni-NTA agarose | Qiagen | 30210 |
| Protein A/G Sepharose beads | Expedeon | AGA1000 |
| GFP-Trap Agarose | Chromotek | gta-20 |
| Calmodulin Sepharose 4B | Cytiva | 17052901 |
| IgG sepharose 6 Fast Flow | Cytiva | 17096901 |
| 3XFLAG peptide | Sigma-Aldrich | F4799 |
| PreScission protease | MRC PPU Reagents and Services | DU34905 |
| TEV protease | MRC PPU Reagents and Services | DU6811 |
| Roche cOmplete EDTA-free protease inhibitor cocktail | Roche | 11873580001 |
| *S. cerevisiae* CMG helicase (expressed in budding yeast) | Deegan et al., 2020, *eLife* | N/A |
| *S. cerevisiae* CMG helicase with Mcm7-KR allele (expressed in budding yeast; Mcm7-KR has K59R, K62R, K69R, K92R, K130R, K135R, K205R, K223R, K306R mutations) | This study | N/A |
| *S. cerevisiae* Uba1 (E1 enzyme for ubiquitylation; expressed in insect cells) | Deegan et al., 2020, *eLife* | N/A |
| *S. cerevisiae* Cdc34 (E2 enzyme for ubiquitylation; expressed in *E. coli*) | Deegan et al., 2020, *eLife* | N/A |
| *S. cerevisiae* SCF^Dia2^ (E3 enzyme for ubiquitylation; expressed in budding yeast) | Deegan et al., 2020, *eLife* | N/A |
| *S. cerevisiae* Ctf4 (replisome factor; expressed in budding yeast) | Yeeles et al., 2015, *Nature* | N/A |
| *S. cerevisiae* Cdc48 (unfoldase; expressed in *E. coli*) | Deegan et al., 2020, *eLife* | N/A |
| *S. cerevisiae* Ufd1-Npl4 (ubiquitin receptors of Cdc48; expressed in *E. coli*) | Deegan et al., 2020, *eLife* | N/A |
| *C. elegans* CDC-48.1 (unfoldase; expressed in *E. coli*) | Xia et al., 2021, *EMBO J.* | N/A |
| *C. elegans* UFD-1_NPL-4.1 (ubiquitin receptors of CDC-48.1; expressed in *E. coli*) | Xia et al., 2021, *EMBO J.* | N/A |
| *C. elegans* UBXN-3 (wt, residues 1-608; *C. elegans* orthologue of mammalian FAF1; expressed in *E. coli*) | Xia et al., 2021, *EMBO J.* | N/A |
| *C. elegans* UBXN-3-∆UBX (residues 1-530; expressed in *E. coli*) | Xia et al., 2021, *EMBO J.* | N/A |
| *C. elegans* UBXN-3-∆435 (residues 436-608; expressed in *E. coli*) | This study | N/A |
| *C. elegans* UBXN-3-∆527 (residues 528-608; expressed in *E. coli*) | This study | N/A |
| *C. elegans* CMG (expressed in budding yeast) | Xia et al., 2021, *EMBO J.* | N/A |
| *C. elegans* UBA-1 (E1 enzyme for ubiquitylation; expressed in insect cells) | Xia et al., 2021, *EMBO J.* | N/A |
| *C. elegans* ULA-1_RFL-1 (E1 enzyme for neddylation; expressed in budding yeast) | Xia et al., 2021, *EMBO J.* | N/A |
| *C. elegans* UBC-12 (E2 for neddylation; expressed in *E. coli*) | Xia et al., 2021, *EMBO J.* | N/A |
| *C. elegans* DCN-1 (E3 for neddylation; expressed in budding yeast) | Xia et al., 2021, *EMBO J.* | N/A |
| *C. elegans* NED-8 (NEDD8; expressed in *E. coli*) | Xia et al., 2021, *EMBO J.* | N/A |
| *C. elegans* LET-70 (priming E2 for ubiquitylation; expressed in *E. coli*) | Xia et al., 2021, *EMBO J.* | N/A |
| *C. elegans* UBC-3 (elongation E2 for ubiquitylation; expressed in *E. coli*) | Xia et al., 2021, *EMBO J.* | N/A |
| *C. elegans* CUL-2^LRR-1^ (E3 for CMG ubiquitylation; expressed in budding yeast) | Xia et al., 2021, *EMBO J.* | N/A |
| *C. elegans* TIM-1_TIPIN-1 (replisome component; expressed in budding yeast) | Xia et al., 2021, *EMBO J.* | N/A |
| *C. elegans* CTF-18_RFC (replisome component; expressed in budding yeast) | Xia et al., 2021, *EMBO J.* | N/A |
| *C. elegans* POLε (replisome component; expressed in budding yeast) | Xia et al., 2021, *EMBO J.* | N/A |
| *C. elegans* CLSP-1 (replisome component; expressed in budding yeast) | Xia et al., 2021, *EMBO J.* | N/A |
| *C. elegans* CTF-4 (replisome component; expressed in budding yeast) | Xia et al., 2021, *EMBO J.* | N/A |
| *C. elegans* MCM-10 (replisome component; expressed in budding yeast) | Xia et al., 2021, *EMBO J.* | N/A |
| Ubiquitin (expressed in *E. coli*) | MRC PPU Reagents and Services | DU20027 |
| Ubiquitin K0 (expressed in *E. coli*) | MRC PPU Reagents and Services | DU24363 |
| Ubiquitin K48R (expressed in *E. coli*) | MRC PPU Reagents and Services | DU20042 |
| 6His-Ulp1 (protease for yeast SUMO Smt3, catalytic domain 403-621; expressed in *E. coli*) | Xia et al., 2021, *EMBO J* | N/A |
| *Human* p97 (unfoldase; expressed in *E. coli*) | This study | N/A |
| *Human* p97 K251A (unfoldase; expressed in *E. coli*) | This study | N/A |
| *Human* p97 E305Q (unfoldase; expressed in *E. coli*) | This study | N/A |
| *Human* p97 K524A (unfoldase; expressed in *E. coli*) | This study | N/A |
| *Human* p97 E578Q (unfoldase; expressed in *E. coli*) | This study | N/A |
| *Human* UFD1-NPL4 (ubiquitin receptors of p97; expressed in *E. coli*) | This study | N/A |
| *Human* UFD1_6His-NPL4 (ubiquitin receptors of p97; expressed in *E. coli*) | This study | N/A |
| *Human* UFD1-NPL4-∆NZF (lacking 581-608aa) (ubiquitin receptors of p97; expressed in *E. coli*) | This study | N/A |
| *Human* UFD1-NPL4-AAE (L238A, W241A, R242E)  (ubiquitin receptors of p97; expressed in *E. coli*) | This study | N/A |
| *Human* UBXN1 (UBX protein; expressed in *E. coli*) | This study | N/A |
| *Human* UBXN7 (wt, residues 1-489; expressed in *E. coli*) | This study | N/A |
| *Human* UBXN7-∆UBX (residues 1-411; expressed in *E. coli*) | This study | N/A |
| *Human* UBXN7-∆UBA (residues 55-489; expressed in *E. coli*) | This study | N/A |
| *Human* UBXN7-∆UIM (residues 1-284 + 305-489; expressed in *E. coli*) | This study | N/A |
| *Human* UBXN7-∆148 (residues 149-489; expressed in *E. coli*) | This study | N/A |
| *Human* FAF1 (wt, residues 1-650; expressed in *E. coli*) | This study | N/A |
| *Human* FAF1-∆UBX (residues 1-570; expressed in *E. coli*) | This study | N/A |
| *Human* FAF1-∆268 (residues 269-650; expressed in *E. coli*) | This study | N/A |
| *Human* FAF1-∆480 (residues 481-650; expressed in *E. coli*) | This study | N/A |
| *Human* FAF1-UBX (residues 571-650; expressed in *E. coli*) | This study | N/A |
| *Human* FAF2-∆M (residues 1-89 + 119-445; expressed in *E. coli*) | This study | N/A |
| *Human* FAF2-∆UBX^∆M^ (residues 1-89 + 119-357 + 442-445; expressed in *E. coli*) | This study | N/A |
| *Human* FAF2-∆137 (residues 138-445; expressed in *E. coli*) | This study | N/A |
| *Human* FAF2-∆297 (residues 298-445; expressed in *E. coli*) | This study | N/A |
| *Human* FAF2-∆CC^∆M^ (residues 1-89 + 119-297 + 351-445; expressed in *E. coli*) | This study | N/A |
| **Experimental Models: Organisms/Strains** |  |  |
| E14tg2A GFP-SLD5 mouse embryonic stem cells | Villa et al., 2021, EMBO R. | N/A |
| E14tg2A GFP-SLD5 FAF1∆ | This study | N/A |
| E14tg2A GFP-SLD5 UBXN7∆ | This study | N/A |
| E14tg2A GFP-SLD5 FAF2-∆UBX | This study | N/A |
| E14tg2A GFP-SLD5 FAF1∆ UBXN7∆ | This study | N/A |
| E14tg2A GFP-SLD5 FAF1∆ UBXN7∆ FAF2-∆UBX | This study | N/A |
| E14tg2A GFP-SLD5 FAF1∆ UBXN7∆ *Rosa26::Human FAF1* | This study | N/A |
| E14tg2A GFP- SLD5 FAF1∆ UBXN7∆ *Rosa26::Human FAF1-∆UBX* | This study | N/A |
| E14tg2A GFP- SLD5 FAF1∆ UBXN7∆ *Rosa26::Human FAF1-∆Coiled coil* | This study | N/A |
| E14tg2A GFP- SLD5 FAF1∆ UBXN7∆ *Rosa26::Human UBXN7* | This study | N/A |
| *yTDK20 (budding yeast strain to purify yeast CMG):*  *MAT***a** */ MATα*  *pep4::kanMX / pep4::kanMX*  *bar1::hph-NT1 / bar1::hph-NT1*  *ade2-1 / ade2-1*  *ura3-1 / ura3-1::pRS306-MCM2-GAL1,10-CBP-TEV-MCM3*  *his3-11::pRS303-CDC45iFLAG2-GAL1,10-GAL4* / *his3-11*  *trp1-1::pRS304-PSF1-GAL1,10-SLD5 / trp1-1::pRS304-MCM5-GAL1,10-MCM4*  *leu2-3::pRS305-PSF2-GAL1,10-PSF3 / leu2-3::pRS305-MCM7-GAL1,10-MCM6*  *ctf4-*I901E / *ctf4-*I901E | Deegan et al., 2020, *eLife* | N/A |
| *yCPR337 (budding yeast strain to purify yeast CMG-Mcm7-KR):*  *MAT***a** */ MATα*  *pep4::kanMX / pep4::kanMX*  *bar1::hph-NT1 / bar1::hph-NT1*  *ade2-1 / ade2-1*  *ura3-1 / ura3-1::pRS306-MCM2-GAL1,10-CBP-TEV-MCM3*  *his3-11::pRS303-CDC45iFLAG2-GAL1,10-GAL4* / *his3-11*  *trp1-1::pRS304-PSF1-GAL1,10-SLD5 / trp1-1::pRS304-MCM5-GAL1,10-MCM4*  *leu2-3::pRS305-PSF2-GAL1,10-PSF3 / leu2-3::pRS305-mcm7-KR-GAL1,10-MCM6*  *ctf4-*I901E / *ctf4-*I901E | This study | N/A |
| *yTDK5 (budding yeast strain to purify yeast SCF^Dia2^):*  *MAT***a** *ade2-1 ura3-1 his3-11,15 trp1-1 leu2-3,112 can1-100 bar1::hphNT pep4::kanMX ura3::pRS306-SKP1+ProteinA-3TEV-DIA2 leu2::pRS305-HRT1+CDC53* | Deegan et al., 2020, *eLife* | N/A |
| *yAE40 (budding yeast strain to purify yeast Ctf4):*  *MAT***a** *ade2-1 ura3-1 his3-11,15 trp1-1 leu2-3,112 can1-100 bar1::hphNT pep4::kanMX his3::pRS303-CBP-TEV-CTF4­+GAL4* | Yeeles et al., 2015, *Nature* | N/A |
| *yYX1 (budding yeast strain to purify worm TIM-1_TIPN-1):*  *MAT***a** *ade2-1 ura3-1 his3-11,15 trp1-1 leu2-3,112 LEU2::pRS305-CBP-PreScissionScission-tipn-1- GAL1,10-tim-1 can1-100 pep4∆::ADE2* | Xia et al., 2021, *EMBO J* | N/A |
| *yYX37 (budding yeast strain to purify worm CMG):*  *MAT***a** */ MATα ade2-1 / ade2-1 ura3-1 URA3::pRS306-mcm-2- GAL1,10-mcm-3 / ura3-1 URA3::pRS306-TAP-psf-1- GAL1,10-sld-5 his3-11,15 / his3-11,15 HIS3::pRS303- GAL1,10-cdc-45 trp1-1 TRP1::pRS304-mcm-6- GAL1,10-mcm-7 / trp1-1 leu2-3,112 LEU2::pRS305- mcm-4-GAL1,10-mcm-5 / leu2-3,112 LEU2::pRS305- psf-3-GAL1,10-psf-2 can1-100 / can1-100 pep4∆::ADE2 / pep4∆::ADE2* | Xia et al., 2021, *EMBO J* | N/A |
| *yYX40 (budding yeast strain to purify worm CLSP-1):*  *MAT***a** *ade2-1 ura3-1 his3-11,15 HIS3::pRS303-GAL1,10-clsp1-TEV-5FLAG trp1-1 leu2-3,112 can1-100 pep4∆::ADE2* | Xia et al., 2021, *EMBO J* | N/A |
| *yYX41(budding yeast strain to purify worm CTF-4):*  *MAT***a** *ade2-1 ura3-1 his3-11,15 HIS-3::pRS303-GAL1,10- 5FLAG-PreScissionScission-ctf-4 trp1-1 leu2-3,112 can1-100 pep4∆::ADE2* | Xia et al., 2021, *EMBO J* | N/A |
| *yYX42 (budding yeast strain to purify worm UBA-1):*  *MAT***a** *ade2-1 ura3-1 his3-11,15 HIS3::pRS303-GAL1,10- ProteinA-TEV-uba-1 trp1-1 leu2-3,112 can1-100 pep4∆::ADE2* | Xia et al., 2021, *EMBO J* | N/A |
| *yYX43 (budding yeast strain to purify worm POLε):*  *MAT***a** *ade2-1 ura3-1 URA3::pRS306-pole-2-GAL1,10-pole-1 his3-11,15 trp1-1 leu2-3,112 LEU2::pRS305-pole-4- TEV-5FLAG-GAL1,10-pole-3 can1-100 pep4∆::ADE2* | Xia et al., 2021, *EMBO J* | N/A |
| *yYX44 (budding yeast strain to purify worm CUL-2^LRR-1^):*  *MAT***a** *ade2-1 ura3-1 URA3::pRS306-cul-2-GAL1,10- rbx-1 his3-11,15 HIS3::pRS303-GAL1,10-lrr-1 trp1-1 leu2-3,112 LEU2::pRS305-TAP-elb-1-GAL1,10-elc-1 can1-100 pep4∆::ADE2* | Xia et al., 2021, *EMBO J* | N/A |
| *yYX48 (budding yeast strain to purify worm CTF-18_RFC):*  *MAT***a** */ MATα ade2-1 / ade2-1 ura3-1 URA3::pRS306- dscc-1-GAL1,10-ctf-8 / ura3-1 his3-11,15 HIS3::pRS303-GAL1,10-ProteinA-3TEV-ctf-18 / his3- 11,15 trp1-1 / trp1-1 TRP1::pRS304-rfc-4-GAL1,10-rfc-5 leu2-3,112 / leu2-3,112 LEU2::pRS305-rfc-2-GAL1,10- rfc-3 can1-100 / can1-100 pep4∆::ADE2 / pep4∆::ADE2* | Xia et al., 2021, *EMBO J* | N/A |
| *yYX58 (budding yeast strain to purify worm ULA-1_RFL-1):*  *MAT***a** *ade2-1 ura3-1 his3-11,15 HIS3::pRS303-rfl-1- GAL1,10-ProteinA-TEV-ula-1a trp1-1 leu2-3,112 can1- 100 pep4∆::ADE2* | Xia et al., 2021, *EMBO J* | N/A |
| **Oligonucleotides (5′-3′)** |  |  |
| Mouse *Lrr1* siRNA  CGACUACAAUGAACCUACA | Horizon Discovery | J-057816-10 |
| Mouse *Faf1* gRNA1_oligo 1: caccgCCGCCGAGCTCCGCGGCTCC | This study | 7288 |
| Mouse *Faf1* gRNA1_oligo 2:  aaacGGAGCCGCGGAGCTCGGCGGc | This study | 7289 |
| Mouse *Faf1* gRNA2_oligo 1: caccgGGACCGGGAGATGATCCTTG | This study | 7290 |
| Mouse *Faf1* gRNA2_oligo 2: aaacCAAGGATCATCTCCCGGTCCc | This study | 7291 |
| Mouse *Ubxn7* gRNA1_oligo 1: caccgACGACGCCGCGGAGCCCCCG | This study | 9214 |
| Mouse *Ubxn7* gRNA1_oligo 2: aaacCGGGGGCTCCGCGGCGTCGTc | This study | 9215 |
| Mouse *Ubxn7* gRNA2_oligo 1: caccgCGGCGTCGTCGGCGCTGAAG | This study | 9216 |
| Mouse *Ubxn7* gRNA2_oligo 2: aaacCTTCAGCGCCGACGACGCCGc | This study | 9217 |
| Mouse *Faf2* gRNA1_oligo 1:  caccgTCTCTCTACTCTGGAATCAT | This study | 9541 |
| Mouse *Faf2* gRNA1_oligo 2: aaacATGATTCCAGAGTAGAGAGAc | This study | 9542 |
| Mouse *Faf2* gRNA2_oligo 1: caccgTCTCACAGTCTCTAACAGTA | This study | 9543 |
| Mouse *Faf2* gRNA2_oligo 2:  aaacTACTGTTAGAGACTGTGAGAc | This study | 9544 |
| Mouse *Faf1* PCR forward primer: GAGTGCATGAGCACCTAGTCTCCCG | This study | 9236 |
| Mouse *Faf1* PCR reverse primer:  CTCAAACAAACGCTGGGAAGAGGCC | This study | 9237 |
| Mouse *Ubxn7* PCR forward primer:  GGAGGGAGCCCCGTGTGTGTCTGTG | This study | 9210 |
| Mouse *Ubxn7* PCR reverse primer:  AGCCCGAGTGAGGAATGCACCAAGG | This study | 9212 |
| Mouse *Faf2* PCR forward primer:  ACCTCCTGAGCTATCCTGTTGGC | This study | 9529 |
| Mouse *Faf2* PCR reverse primer: CCTGAAGGTTCCATCCCAGGGAC | This study | 9530 |
| T3  AATTAACCCTCACTAAAGGG | This study | N/A |
| p97 forward primer for construction of pRF001:  gaacagattggtggcATGGCTTCTGGAGCCGATTCAAAAGGTG | This study | 7908 |
| p97 reverse primer for construction of pRF001:  gtgcggccgcttattaGCCATACAGGTCATCATCATTGTCTTCTG | This study | 7909 |
| p97 (K251A) mutagenesis primer for construction of pRF071: TGGAACAGGAgcGACCCTGATTGCTCGAGCTG | This study | 9196 |
| p97 (K251A) mutagenesis primer for construction of pRF071:  AATCAGGGTCgcTCCTGTTCCAGGAGGTCCG | This study | 9197 |
| p97 (E305Q) mutagenesis primer for construction of pRF072: CCTGGCTGTGGGgcAACTTTGTTGGCCAAAG | This study | 9198 |
| p97 (E305Q) mutagenesis primer for construction of pRF072:  CAACAAAGTTgcCCCACAGCCAGGAGGTCC | This study | 9199 |
| p97 (K524A) mutagenesis primer for construction of pRF073:  CTTCATTGATcAGCTAGATGCCATCGCTCCC | This study | 9200 |
| p97 (K524A) mutagenesis primer for construction of pRF073:  GCATCTAGCTgATCAATGAAGATGATGGCAGG | This study | 9201 |
| p97 (E578Q) mutagenesis primer for construction of pRF074:  ATTCTTTGATcAGCTGGATTCGATTGCCAAGG | This study | 9202 |
| p97 (E578Q) mutagenesis primer for construction of pRF074:  GAATCCAGCTgATCAAAGAATAGCACACAGG | This study | 9203 |
| *Ufd1* forward primer for construction of pRF003:  gaacagattggtggcATGTTCTCTTTCAACATGTTCGACCACC | This study | 7912 |
| *Ufd1* reverse primer for construction of pRF003:  gtgcggccgcttattaGGGCTTTCTTCCCTTTTTACGCAATGAC | This study | 7913 |
| *Npl4* forward primer for construction of pRF004:  gcccatatgATGGCCGAGAGCATCATAATTCGTGTC | This study | 7918 |
| *Npl4* reverse primer for construction of pRF004:  gccctcgagCTAGGTCCTGGGGAGGCTGCACATCTC | This study | 7919 |
| *Npl4* forward primer for construction of pRF016:  gccccATGGCCGAGAGCATCATAATTCGTG | This study | 8529 |
| *Npl4* reverse primer for construction of pRF016:  gccgaattcCTAGGTCCTGGGGAGGCTGCACATCTC | This study | 8195 |
| NPL4-∆NZF forward primer for construction of pRF150:  tttaagaaggagatataccATGGCCGAGAGCATC | This study | 9772 |
| NPL4-∆NZF reverse primer for construction of pRF150:  tggtggtggtggtgctcgagttaGGCCGTGTGTGTGGAGCC | This study | 9773 |
| NPL4-AAE (L238A, W241A, R242E) mutagenesis primer for construction of pRF135:  GCTTTgcTGACTTCgcGgaAAAGACAGGGAACCAGC | This study | 9688 |
| NPL4-AAE (L238A, W241A, R242E) mutagenesis primer for construction of pRF135:  GTCTTTtcCgcGAAGTCAgcAAAGCGGTCAGCGACGG | This study | 9689 |
| *Faf1* forward primer for construction of pRF002:  gaacagattggtggcATGGCGTCCAACATGGACCGGGAGATG | This study | 7904 |
| *Faf1* reverse primer for construction of pRF002, pRF049, pRF051:  gtgcggccgcttattaCTCTTTTGCTTCAAGGAAAAGGGTTTCTTG | This study | 7905 |
| FAF1-∆UBX mutagenesis primer for construction of pRF043:  gccgcTTAAGCATTTTCTTCCTTTGGCTCAGG | This study | 9029 |
| FAF1-∆UBX mutagenesis primer for construction of pRF043:  AAAATGCTTAAgcggccgcacatcaccatc | This study | 9030 |
| FAF1-UBX forward primer for construction of pRF047:  gaacagattggtggcGAGCCTGTGAGCAAACTGCGG | This study | 9043 |
| FAF1-UBX forward primer for construction of pRF049:  gaacagattggtggcTCTTCACCTGCACAGACCCG | This study | 9126 |
| FAF1-∆480 forward primer for construction of pRF051:  gaacagattggtggcATGGCTGCAATGGAGATCTTC | This study | 9128 |
| *Faf2* forward primer for construction of pRF044:  gaacagattggtggcATGGCGGCGCCTGAGGAG | This study | 8895 |
| *Faf2* reverse primer for construction of pRF044, pRF086, pRF087:  gtgcggccgcttattaTCATTCGTCAGTTAGGTCCTG | This study | 8896 |
| FAF2^∆M^ mutagenesis primer for construction of pRF044:  TATAAAACGCCTTGGTTGAGGTCTTGAGAC | This study | 9033 |
| FAF2^∆M^ mutagenesis primer for construction of pRF044:  CAACCAAGGCGTTTTATACGGCCTGACCCTC | This study | 9034 |
| FAF2-∆137 forward primer for construction of pRF086:  gaacagattggtggcTCATTTATGCACTCTTTTG | This study | 9294 |
| FAF2-∆297 forward primer for construction of pRF087:  gaacagattggtggcTTTATCATGGATGCTAACCAG | This study | 9295 |
| FAF2-∆CC^∆M^ mutagenesis primer for construction of pRF088:  tcagggggGTAGGCCTCATCCTGCTGTTG | This study | 9296 |
| FAF2-∆CC^∆M^ mutagenesis primer for construction of pRF088:  aggcctacCCCCCTGAACCTTCCCCTG | This study | 9297 |
| FAF2-∆UBX^∆M^ mutagenesis primer for construction of pRF089:  tcagttagATCAGGGGAAGGTTCAGGG | This study | 9298 |
| FAF2-∆UBX^∆M^ mutagenesis primer for construction of pRF089:  cccctgatCTAACTGACGAATGAtaataag | This study | 9299 |
| *Ubxn1* forward primer for construction of pRF031:  gaacagattggtggcATGGCGGAGCTGACGGCTC | This study | 8901 |
| *Ubxn1* reverse primer for construction of pRF031:  gtgcggccgcttattaTCAGCTGGGACATTTCTTGG | This study | 8902 |
| *Ubxn7* forward primer for construction of pRF017:  gaacagattggtggcATGGCTGCCCACGGGGGCTCCGCGGCGTC | This study | 8530 |
| UBXN7 reverse primer for construction of pRF017, pRF052:  gtgcggccgcttattaTTAATTTCTTTCCTGTACAAAGACAGTC | This study | 8531 |
| UBXN7-∆148 forward primer for construction of pRF052:  gaacagattggtggcATGCATAAAGGCAGCTTTG | This study | 9136 |
| UBXN7-∆UBA mutagenesis primer for construction of pRF116:  CTTCAGCGATGCCACCAATCTGTTCTCTGTG | This study | 9467 |
| UBXN7-∆UBA mutagenesis primer for construction of pRF116:  GATTGGTGGCATCGCTGAAGAGCCCAGTACCAG | This study | 9468 |
| UBXN7-∆UIM mutagenesis primer for construction of pRF117:  GTCTGTGTTGATGCATCTATAAGGCTCTCTG | This study | 9469 |
| UBXN7-∆UIM mutagenesis primer for construction of pRF117:  TTATAGATGCATCAACACAGACAAAACAGG | This study | 9470 |
| UBXN7-∆UBX mutagenesis primer for construction of pRF118:  cggccgcTTATGGTCCATTTACATCTATC | This study | 9471 |
| UBXN7-∆UBX mutagenesis primer for construction of pRF118:  TAAATGGACCATAAgcggccgcacatcacc | This study | 9472 |
| UBXN-3-∆435 forward primer for construction of pRF056:  gaacagattggtggcTTGAGCGGTGTCTCGGAGTAC | This study | 9140 |
| UBXN-3 reverse primer for construction of pRF056, pRF057:  gtgcggccgcttattaGATTTCCTCGACAAAAATCTGCTCCCTGG | This study | 7907 |
| UBXN-3-∆527 forward primer for construction of pRF057:  gaacagattggtggcGCTGAGATTATCAATGTCAAG | This study | 9141 |
| *Faf1* forward primer for construction of pRF138, 139, 142:  ctgcagtcgacggtaccaccATGGCGTCCAACATGGACC | This study | 9690 |
| *Faf1* reverse primer for construction of pRF138, 142:  ggcagagggaaaaagatctTTACTCTTTTGCTTCAAGG | This study | 9691 |
| *Faf1* reverse primer for construction of pRF139:  ggcagagggaaaaagatctAGCATTTTCTTCCTTTGGCTC | This study | 9692 |
| *Ubxn7* forward primer for construction of pRF140:  ctgcagtcgacggtaccaccATGGCTGCCCACGGGGGCTC | This study | 9693 |
| *Ubxn7* reverse primer for construction of pRF140:  ggcagagggaaaaagatctTTAATTTCTTTCCTGTACAAAGACAGTCTC | This study | 9699 |
|  |  |  |
| **Recombinant DNA (Plasmids)** |  |  |
| Yeast Ufd1 in K27SUMO [bacterial expression of yeast Ufd1 with 14His-Smt3 in pK27SUMO vector] | Stein et al., 2014, *Cell* | N/A |
| Yeast Npl4 in pET21b [bacterial expression of yeast Npl4 with pET21b vector] | Stein et al., 2014, *Cell* | N/A |
| Yeast Cdc48 in K27SUMO [bacterial expression of yeast Cdc48 with 14His-Smt3 in pK27SUMO vector] | Stein et al., 2014, *Cell* | N/A |
| pTDK7 [bacterial expression of yeast Cdc34 with pET28c vector] | Deegan et al., 2020, *eLife* | N/A |
| pET15b 6His-TEV-UBC-3 [bacterial expression of 6HIS-TEV-UBC3] | Xia et al., 2021, *EMBO J* | DU62204 |
| pET15b 6His-TEV-LET-70 [bacterial expression of 6His-TEV-LET-70] | Xia et al., 2021, *EMBO J* | DU62217 |
| pYXC72 [bacterial expression of worm UBC-12 with 14His-Smt3 in pK27SUMO vector] | Xia et al., 2021, *EMBO J* | DU70362 |
| pYXC73 [bacterial expression of worm DCN-1 with 14His-Smt3 in pK27SUMO vector] | Xia et al., 2021, *EMBO J* | DU70363 |
| pYXC74 [bacterial expression of worm NED-8 with 14His-Smt3 in pK27SUMO vector] | Xia et al., 2021, *EMBO J* | DU70364 |
| pYXC53 [bacterial expression of worm MCM-10 with 14His-Smt3 in pK27SUMO vector] | Xia et al., 2021, *EMBO J* | DU70357 |
| pRF005 [bacterial expression of worm CDC-48.1 with 14His-Smt3 in pK27SUMO vector] | Xia et al., 2021, *EMBO J* | DU70365 |
| pRF008 [bacterial expression of worm NPL-4.1 in pET28c vector] | Xia et al., 2021, *EMBO J* | DU70368 |
| pRF007 [bacterial expression of worm UFD-1 with 14His-Smt3 in pK27SUMO vector] | Xia et al., 2021, *EMBO J* | DU70367 |
| pRF006 [bacterial expression of worm UBXN-3 with 14His-Smt3 in pK27SUMO vector] | Xia et al., 2021, *EMBO J* | DU70366 |
| pRF099 [bacterial expression of worm UBXN-3-∆UBX with 14His-Smt3 in pK27SUMO vector] | Xia et al., 2021, *EMBO J* | DU70600 |
| pRF056 [bacterial expression of worm UBXN-3-∆435 with 14His-Smt3 in pK27SUMO vector] | This study | DU 73334 |
| pRF057 [bacterial expression of worm UBXN-3-∆527 with 14His-Smt3 in pK27SUMO vector] | This study | DU 73335 |
| pRF001 [bacterial expression of human p97 with 14His-Smt3 in pK27SUMO vector] | This study | DU 73310 |
| pRF071 [bacterial expression of human p97 (K251A) with 14His-Smt3 in pK27SUMO vector] | This study | DU 73311 |
| pRF072 [bacterial expression of human p97 (E305Q) with 14His-Smt3 in pK27SUMO vector] | This study | DU 73312 |
| pRF073 [bacterial expression of human p97 (K524A) with 14His-Smt3 in pK27SUMO vector] | This study | DU 73313 |
| pRF074 [bacterial expression of human p97 (E578Q) with 14His-Smt3 in pK27SUMO vector] | This study | DU 73314 |
| pRF003 [bacterial expression of human UFD1 with 14His-Smt3 in pK27SUMO vector] | This study | DU 73315 |
| pRF004 [bacterial expression of human NPL4 with 6His in pET28c vector] | This study | DU 73316 |
| pRF016 [bacterial expression of human NPL4 in pET28c vector] | This study | DU 73317 |
| pRF150 [bacterial expression of human NPL4-∆NZF in pET28c vector] | This study | DU 75133 |
| pRF135 [bacterial expression of human NPL4-AAE (L238A, W241A, R242E) in pET28c vector] | This study | DU 75134 |
| pRF002 [bacterial expression of human FAF1 with 14His-Smt3 in pK27SUMO vector] | This study | DU 73318 |
| pRF043 [bacterial expression of human FAF1-∆UBX with 14His-Smt3 in pK27SUMO vector] | This study | DU 73319 |
| pRF047 [bacterial expression of human FAF1-UBX with 14His-Smt3 in pK27SUMO vector] | This study | DU 73320 |
| pRF049 [bacterial expression of human FAF1-∆268 with 14His-Smt3 in pK27SUMO vector] | This study | DU 73321 |
| pRF051 [bacterial expression of human FAF1-∆480 with 14His-Smt3 in pK27SUMO vector] | This study | DU 73322 |
| pRF044 [bacterial expression of human FAF2^∆M^ with 14His-Smt3 in pK27SUMO vector] | This study | DU 73323 |
| pRF086 [bacterial expression of human FAF2-∆137 with 14His-Smt3 in pK27SUMO vector] | This study | DU 73324 |
| pRF087 [bacterial expression of human FAF2-∆297 with 14His-Smt3 in pK27SUMO vector] | This study | DU 73325 |
| pRF088 [bacterial expression of human FAF2-∆CC^∆M^ with 14His-Smt3 in pK27SUMO vector] | This study | DU 73326 |
| pRF089 [bacterial expression of human FAF2-∆UBX^∆M^ with 14His-Smt3 in pK27SUMO vector] | This study | DU 73327 |
| pRF031 [bacterial expression of human UBXN1 with 14His-Smt3 in pK27SUMO vector] | This study | DU 73328 |
| pRF017 [bacterial expression of human UBXN7 with 14His-Smt3 in pK27SUMO vector] | This study | DU 73329 |
| pRF052 [bacterial expression of human UBXN7-∆148 with 14His-Smt3 in pK27SUMO vector] | This study | DU 73330 |
| pRF116 [bacterial expression of human UBXN7-∆UBA with 14His-Smt3 in pK27SUMO vector] | This study | DU 73331 |
| pRF117 [bacterial expression of human UBXN7-∆UIM with 14His-Smt3 in pK27SUMO vector] | This study | DU 73332 |
| pRF118 [bacterial expression of human UBXN7-∆UBX with 14His-Smt3 in pK27SUMO vector] | This study | DU 73333 |
| pCPR037 [Yeast expression of yeast Mcm7-KR and Mcm6 in pRS305 vector with *LEU2* marker gene] | This study | DU 75142 |
| pASF007 [gRNA1 mouse FAF1; based on pX335] | This study | DU 73336 |
| pASF008 [gRNA2 mouse FAF1; based on pKN7] | This study | DU 73337 |
| pRF076 [gRNA1 mouse UBXN7; based on pX335] | This study | DU 73338 |
| pRF077 [gRNA2 mouse UBXN7; based on pKN7] | This study | DU 73339 |
| pRF120 [gRNA1 mouse FAF2; based on pX335] | This study | DU 73340 |
| pRF121 [gRNA2 mouse FAF2; based on pKN7] | This study | DU 73341 |
| pKN88 [gRNA ROSA26; based on pKN101] | Villa et al., 2021, *EMBO R* | DU 70256 |
| pRF138 [donor vector for human FAF1] | This study | DU 75047 |
| pRF139 [donor vector for human FAF1-∆UBX] | This study | DU 75048 |
| pRF140 [donor vector for human UBXN7] | This study | DU 75049 |
| pRF142 [donor vector for human FAF1-∆Coiled coil] | This study | DU 75051 |
|  |  |  |
| **Software and Algorithms** |  |  |
| ImageJ | National Institute of Health | https://imagej.nih.gov/ij/ |
| ZEN blue software | Zeiss | https://www.zeiss.co.jp/microscopy/products/microscope-software/zen.html |
| Prism 9 | GraphPad | https://www.graphpad.com/scientific-software/prism/ |
