# Supplementary material for: Multiple UBX proteins reduce the ubiquitin threshold of the mammalian p97-UFD1-NPL4 unfoldase
Source: eLife. 2022 Aug 3;11:e76763. doi: 10.7554/eLife.76763 (PMC9377798; doi:10.7554/eLife.76763)
Supplement: Supplementary file 2. [file elife-76763-supp2.docx]

**Supplementary File 2**

Plasmids generated in this study. The following plasmids are available from MRC PPU Reagents and Services (<https://mrcppureagents.dundee.ac.uk/>) with the indicated identifiers.

| Plasmid | Markers | Parental Vector | Insert | Expression  host | Identifier |
| --- | --- | --- | --- | --- | --- |
| pRF001 | KanR | pK27SUMO | 14His-Smt3-human p97 (PCR & Gibson cloning; oligos 7908+7909) | *E. coli* | DU 73310 |
| pRF071 | KanR | pK27SUMO | 14His-Smt3-human p97 K251A (PCR mutagenesis from pRF001 by oligos 9196+9197) | *E. coli* | DU 73311 |
| pRF072 | KanR | pK27SUMO | 14His-Smt3-human p97 E305Q (PCR mutagenesis from pRF001 by oligos 9198+9199) | *E. coli* | DU 73312 |
| pRF073 | KanR | pK27SUMO | 14His-Smt3-human p97 K524A (PCR mutagenesis from pRF001 by oligos 9200+9201) | *E. coli* | DU 73313 |
| pRF074 | KanR | pK27SUMO | 14His-Smt3-human p97 E578Q (PCR mutagenesis from pRF001 by oligos 9202+9203) | *E. coli* | DU 73314 |
| pRF003 | KanR | pK27SUMO | 14His-Smt3-human UFD1 (PCR & Gibson cloning; oligos 7912+7913) | *E. coli* | DU 73315 |
| pRF004 | KanR | pET28c | 6His-human NPL4 (PCR by oligos 7918+7919; NdeI-XhoI fragment) | *E. coli* | DU 73316 |
| pRF016 | KanR | pET28c | Human NPL4 (PCR by oligos 8529+8195; NcoI-EcoRI fragment) | *E. coli* | DU 73317 |
| pRF150 | KanR | pET28c | Human NPL4-∆NZF (PCR & Gibson cloning; oligos 9772+9773) | *E. coli* | DU 75133 |
| pRF135 | KanR | pET28c | Human NPL4 -AAE (L238A, W241A, R242E) (PCR mutagenesis from pRF016 by oligos 9688+9689) | *E. coli* | DU 75134 |
| pRF002 | KanR | pK27SUMO | 14His-Smt3-human FAF1 (PCR & Gibson cloning; oligos 7904+7905) | *E. coli* | DU 73318 |
| pRF043 | KanR | pK27SUMO | 14His-Smt3-human FAF1-∆UBX (PCR mutagenesis from pRF002 by oligos 9029+9030) | *E. coli* | DU 73319 |
| pRF047 | KanR | pK27SUMO | 14His-Smt3-human FAF1-UBX (PCR & Gibson cloning; oligos 9043+7905) | *E. coli* | DU 73320 |
| pRF049 | KanR | pK27SUMO | 14His-Smt3-human FAF1-∆268 (PCR & Gibson cloning; oligos 9126+7905) | *E. coli* | DU 73321 |
| pRF051 | KanR | pK27SUMO | 14His-Smt3-human FAF1-∆480 (PCR & Gibson cloning; oligos 9128+7905) | *E. coli* | DU 73322 |
| pRF044 | KanR | pK27SUMO | 14His-Smt3-human FAF2^∆M^ (PCR & Gibson cloning; oligos 8895+8896, then PCR mutagenesis by oligos 9029+9030) | *E. coli* | DU 73323 |
| pRF086 | KanR | pK27SUMO | 14His-Smt3-human FAF2-∆137 (PCR & Gibson cloning; from pRF044 by oligos 9294+8896) | *E. coli* | DU 73324 |
| pRF087 | KanR | pK27SUMO | 14His-Smt3-human FAF2-∆297 (PCR & Gibson cloning; from pRF044 by oligos 9295+8896) | *E. coli* | DU 73325 |
| pRF088 | KanR | pK27SUMO | 14His-Smt3-human FAF2-∆CC^∆M^ (PCR mutagenesis from pRF044 by oligos 9296+9297) | *E. coli* | DU 73326 |
| pRF089 | KanR | pK27SUMO | 14His-Smt3-human FAF2-∆UBX^∆M^ (PCR mutagenesis from pRF044 by oligos 9298+9299) | *E. coli* | DU 73327 |
| pRF031 | KanR | pK27SUMO | 14His-Smt3-human UBXN1 (PCR & Gibson cloning; oligos 8901+8902) | *E. coli* | DU 73328 |
| pRF017 | KanR | pK27SUMO | 14His-Smt3-human UBXN7 (PCR & Gibson cloning; oligos 8530+8531) | *E. coli* | DU 73329 |
| pRF052 | KanR | pK27SUMO | 14His-Smt3-human UBXN7-∆148 (PCR & Gibson cloning; from pRF017 by oligos 9136+8531) | *E. coli* | DU 73330 |
| pRF116 | KanR | pK27SUMO | 14His-Smt3-human UBXN7-∆UBA (PCR & Gibson cloning; from pRF017 by oligos 9467+9468) | *E. coli* | DU 73331 |
| pRF117 | KanR | pK27SUMO | 14His-Smt3-human UBXN7-∆UIM (PCR mutagenesis from pRF017 by oligos 9469+9470) | *E. coli* | DU 73332 |
| pRF118 | KanR | pK27SUMO | 14His-Smt3-human UBXN7-∆UBX (PCR mutagenesis from pRF017 by oligos 9471+9472) | *E. coli* | DU 73333 |
| pRF056 | KanR | pK27SUMO | 14His-Smt3-worm UBXN-3-∆435 (PCR & Gibson cloning by oligos 9140+7907) | *E. coli* | DU 73334 |
| pRF057 | KanR | pK27SUMO | 14His-Smt3-worm UBXN-3-∆527 (PCR & Gibson cloning by oligos 9141+7907) | *E. coli* | DU 73335 |
| pCPR037 | AmpR | pRS305 | Mcm6 and Mcm7-KR  (K59R, K62R, K69R, K92R, K130R, K135R, K205R, K223R, K306R mutations in Mcm7) | *Budding yeast* | DU 75142 |
| pASF007 | AmpR | pX335 | gRNA1 FAF1 (oligos 7288+7289 ligated into BbsI-digested pX335) | *Mammalian cells* | DU 73336 |
| pASF008 | KanR | pKN7 | gRNA2 FAF1 (oligos 7290+7291 ligated into BbsI-digested pKN7) | *Mammalian cells* | DU 73337 |
| pRF076 | AmpR | pX335 | gRNA1 UBXN7 (oligos 9214+9215 ligated into BbsI-digested pX335) | *Mammalian cells* | DU 73338 |
| pRF077 | KanR | pKN7 | gRNA2 UBXN7 (oligos 9216+9217 ligated into BbsI-digested pKN7) | *Mammalian cells* | DU 73339 |
| pRF120 | AmpR | pX335 | gRNA1 FAF2 (oligos 9541+9542 ligated into BbsI-digested pX335) | *Mammalian cells* | DU 73340 |
| pRF121 | KanR | pKN7 | gRNA2 FAF2 (oligos 9543+9544 ligated into BbsI-digested pKN7) | *Mammalian cells* | DU 73341 |
| pRF138 | AmpR | pKN18 | donor vector for human FAF1 (PCR & Gibson cloning by oligos 9690+9691 from pRF002 into KpnI-BglII-digested pKN18) | *Mammalian cells* | DU75047 |
| pRF139 | AmpR | pKN18 | donor vector for human FAF1-∆UBX (PCR & Gibson cloning by oligos 9690+9692 from pRF002 into KpnI-BglII-digested pKN18) | *Mammalian cells* | DU75048 |
| pRF140 | AmpR | pKN18 | donor vector for human UBXN7  (PCR & Gibson cloning by oligos 9693+9699 from pRF017 into KpnI-BglII-digested pKN18) | *Mammalian cells* | DU75049 |
| pRF142 | AmpR | pKN18 | donor vector for human FAF1-∆Coiled coil  (PCR & Gibson cloning by oligos 9690+9691 from pRF137 into KpnI-BglII-digested pKN18) | *Mammalian cells* | DU75051 |
